# Synergetic association between coxsackievirus A16 genotype evolution and recombinant form shifts

**DOI:** 10.1093/ve/vead080

**Published:** 2023-12-20

**Authors:** Zhenzhi Han, Fangming Wang, Jinbo Xiao, Hanhaoyu Fu, Yang Song, Mingli Jiang, Huanhuan Lu, Jichen Li, Yanpeng Xu, Runan Zhu, Yong Zhang, Linqing Zhao

**Affiliations:** Laboratory of Virology, Beijing Key Laboratory of Etiology of Viral Diseases in Children, Capital Institute of Pediatrics, Yabao Road, Chaoyang District, Beijing 100020, China; WHO WPRO Regional Polio Reference Laboratory, NHC Key Laboratory for Biosafety, NHC Key Laboratory for Medical Virology, National Institute for Viral Disease Control and Prevention, Chinese Center for Disease Control and Prevention, No.155 Changbai Road, Changping District, Beijing 102206, People’s Republic of China; Laboratory of Virology, Beijing Key Laboratory of Etiology of Viral Diseases in Children, Capital Institute of Pediatrics, Yabao Road, Chaoyang District, Beijing 100020, China; WHO WPRO Regional Polio Reference Laboratory, NHC Key Laboratory for Biosafety, NHC Key Laboratory for Medical Virology, National Institute for Viral Disease Control and Prevention, Chinese Center for Disease Control and Prevention, No.155 Changbai Road, Changping District, Beijing 102206, People’s Republic of China; Laboratory of Virology, Beijing Key Laboratory of Etiology of Viral Diseases in Children, Capital Institute of Pediatrics, Yabao Road, Chaoyang District, Beijing 100020, China; WHO WPRO Regional Polio Reference Laboratory, NHC Key Laboratory for Biosafety, NHC Key Laboratory for Medical Virology, National Institute for Viral Disease Control and Prevention, Chinese Center for Disease Control and Prevention, No.155 Changbai Road, Changping District, Beijing 102206, People’s Republic of China; WHO WPRO Regional Polio Reference Laboratory, NHC Key Laboratory for Biosafety, NHC Key Laboratory for Medical Virology, National Institute for Viral Disease Control and Prevention, Chinese Center for Disease Control and Prevention, No.155 Changbai Road, Changping District, Beijing 102206, People’s Republic of China; Laboratory of Virology, Beijing Key Laboratory of Etiology of Viral Diseases in Children, Capital Institute of Pediatrics, Yabao Road, Chaoyang District, Beijing 100020, China; Laboratory of Virology, Beijing Key Laboratory of Etiology of Viral Diseases in Children, Capital Institute of Pediatrics, Yabao Road, Chaoyang District, Beijing 100020, China; WHO WPRO Regional Polio Reference Laboratory, NHC Key Laboratory for Biosafety, NHC Key Laboratory for Medical Virology, National Institute for Viral Disease Control and Prevention, Chinese Center for Disease Control and Prevention, No.155 Changbai Road, Changping District, Beijing 102206, People’s Republic of China; Laboratory of Virology, Beijing Key Laboratory of Etiology of Viral Diseases in Children, Capital Institute of Pediatrics, Yabao Road, Chaoyang District, Beijing 100020, China; Laboratory of Virology, Beijing Key Laboratory of Etiology of Viral Diseases in Children, Capital Institute of Pediatrics, Yabao Road, Chaoyang District, Beijing 100020, China

**Keywords:** Coxsackievirus A16 (CVA16), hand, foot, and mouth disease (HFMD), recombinant forms (RFs), population dynamics

## Abstract

Coxsackievirus A16 (CVA16) is a major pathogen that causes hand, foot, and mouth disease (HFMD). The recombination form (RF) shifts and global transmission dynamics of CVA16 remain unknown. In this retrospective study, global sequences of CVA16 were retrieved from the GenBank database and analyzed using comprehensive phylogenetic inference, RF surveys, and population structure. A total of 1,663 sequences were collected, forming a 442-sequences dataset for *VP1* coding region analysis and a 345-sequences dataset for RF identification. Based on the *VP1* coding region used for serotyping, three genotypes (A, B, and D), two subgenotypes of genotype B (B1 and B2), and three clusters of subgenotype B1 (B1a, B1b, and B1c) were identified. Cluster B1b has dominated the global epidemics, B2 disappeared in 2000, and D is an emerging genotype dating back to August 2002. Globally, four oscillation phases of CVA16 evolution, with a peak in 2013, and three migration pathways were identified. Europe, China, and Japan have served as the seeds for the global transmission of CVA16. Based on the *3D* coding region of the RFs, five clusters of RFs (RF-A to -E) were identified. The shift in RFs from RF-B and RF-C to RF-D was accompanied by a change in genotype from B2 to B1a and B1c and then to B1b. In conclusion, the evolution and population dynamics of CVA16, especially the coevolution of *3D* and *VP1* genes, revealed that genotype evolution and RF replacement were synergistic rather than stochastic.

## 1. Introduction

Coxsackievirus A16 (CVA16) is a member of the *Enteroviruses* and an important pathogen responsible for hand, foot, and mouth disease (HFMD). CVA16 has caused several HFMD outbreaks in many countries including Vietnam, Australia, Singapore, India, China, France, and Canada, demonstrating a trend as a global epidemic (Robinson, Doane, and Rhodes 1958; Ferson and Bell 1991; Van Tu et al. 2007; Zhang et al. 2010; Ang et al. 2015; Hassel et al. 2017; Han et al. 2020; Tang et al. 2022). Although other enteroviruses (EVs), such as EV-A71, CVA6, CVA10, and CVA4, also play noteworthy roles in HFMD worldwide (Zhang et al. 2010; Song et al. 2017; Yang et al. 2018; Wang et al. 2019), the number of CVA16-related HFMD cases increased from 2017 to 2019, following the introduction of inactivated EV-A71 vaccines in China, which reduced EV-A71-related HFMD cases (Ji et al. 2019; Han et al. 2020). CVA6 has gradually the dominant serotype in mainland China, causing HFMD epidemics in certain areas (Song et al. 2017, 2020; Anh et al. 2018). Recently, a local government hospital in India reported that more than 82 children under the age of five years had a round red skin rash similar to tomatoes, which was defined as an outbreak of ‘tomato flu’ (Chavda, Patel, and Apostolopoulos 2023). Subsequently, CVA16 was found to be the pathogen of the ‘tomato flu’ disease, which is an atypical manifestation of HFMD (Tang et al. 2022; Chavda, Patel, and Apostolopoulos 2023).

CVA16, as with other members of the *Enteroviruses,* has a single positive-strand genome of approximately 7.5 kb and is classified as species A of the genus *Enterovirus* (EV-A) in the family *Picornaviridae* (Zell et al. 2017). Two open reading frames (ORFs) have been identified in the CVA16 genome: a typically long ORF1 and a second small ORF2 (Guo et al. 2019; Lulla et al. 2019). ORF1, flanked by a 5′-untranslated region (UTR) and a 3′-UTR, produces the precursors of mature viral proteins P1, P2, and P3, which are further cleaved into structural proteins VP4, VP2, VP3, and VP1, and nonstructural proteins 2A–2C and 3A–3D (Zhang et al. 2010; Mao et al. 2014; Baggen et al. 2018). ORF2, located upstream of the CVA16 genome, is a determining factor in viral intestinal infections (Guo et al. 2019; Lulla et al. 2019). Fecal–oral transmission is the main route of most enteroviruses, although some serotypes can spread through the respiratory tract, such as enterovirus D68 (EV-D68) (Tan et al. 2016).

The *VP1* coding region, encoding the structural protein containing major antigen-neutralization, has been used as a target in EV molecular serotyping (Oberste et al. 1999a, 1999b) and has become the gold standard for EV typing and detection (Oberste et al. 2000, 2003). In addition, experimental recombinant VP1 vaccines have been developed, although they have not yet been licensed (Yee and Poh 2015). By targeting the full-length or partial *VP1* coding region, novel EV serotypes, including CVA16, have been identified (Oberste, Maher, and Pallansch 2002; Oberste et al. 2004, 2005, 2007; Han et al. 2018, 2021). Many genotypes of CVA16 are spreading globally: a new genotype (D) appeared in France and then spread to Shanghai, China, revealing the continuous evolution of CVA16 and the need to study the evolutionary characteristics of the *VP1* coding region.

Recombinant forms (RFs) are defined based on the hotspot region for enterovirus recombination and the *3D* coding region of the near-full-length genome. The *3D* error-prone RNA-dependent RNA polymerases (RdRps) of EV lead to misincorporations during genome replication. In recent EV studies, different bootstrap-supported clusters in the *3D* phylogeny have been designated as RFs because *3D* represents the noncapsid coding region farthest from *VP1* (Gaunt et al. 2015; Puenpa et al. 2016; Song et al. 2020). With this rapid increase in prevalence, global CVA6 variants have been divided into twenty-three RFs, named RF-A to RF-X (Puenpa et al. 2022). Despite the several clinical and epidemiological descriptions, the RFs and global transmission modes of CVA16 remain unclear.

In this retrospective study, sequences of CVA16 from the GenBank database from around the world were retrieved and analyzed using comprehensive phylogenetic inference, an RFs survey, and population structure based on time, geography, and specimen types.

## 2. Materials and methods

### 2.1 Retrieval and dataset construction of CVA16 sequences

All CVA16 sequences were retrieved from the NCBI GenBank database, which is publicly available worldwide (https://www.ncbi.nlm.nih.gov/genbank/), using keywords ‘Coxsackievirus A16’ or ‘CVA16’. The sequences were first extracted according to the following exclusion criteria: sequences of the *VP1* gene less than 891 nucleotides (the *VP1* coding region) and low-quality or repetitive sequences, as determined using Seqkit software (Fig. S1). Low-quality sequences were defined as sequences from clones and strains with high passage numbers and many undetermined bases, or sequences that were misnamed as other serotypes. Among the first sequences extracted, those labeled with the type of specimen (e.g. feces, throat swabs, or cerebrospinal fluid), collection date, and isolation region were used to construct a primary CVA16 dataset for geographical and evolutionary analyses (the 1663-dataset). Subsequently, a new dataset was constructed from the primary CVA16 dataset for genotyping CVA16 and building a global temporal–spatial transmission pattern (the 442-dataset). To confirm the transmission pattern and construct new datasets, the sequences covering all the clusters, regions, and times shown in the phylogenetic tree were extracted four times randomly based on their comprehensive phylogenetic relationships, time, and geographical distributions to get their common spatial migration pathways, and the most representative one was selected to constitute the 442-dataset. Next, near-full-length sequences of >7,000 nucleotides including *VP1* and *3D* coding regions were collected to construct another new dataset (the 345-dataset), which was used to analyze the replacement of the CVA16 RF and the correlation between RF replacement and genotype evolution.

### 2.2 Phylogenetic analysis

All sequences were edited using MEGA software (version 7.0) and aligned using MAFFT software (version v7.407) with the L-INS-I parameter (Katoh and Standley 2013; Kumar, Stecher, and Tamura 2016). The maximum likelihood tree was constructed using IQ-TREE software (version 1.6.8) with 1,000 bootstrap replicates. The general time-reversible (GTR) nucleotide substitution model and gamma distribution of rate variation (GTR + G4) were chosen for different datasets using the ModelFinder approach (Nguyen et al. 2015; Kalyaanamoorthy et al. 2017). CVA16 sequences were genotyped using the neighbor-joining method in MEGA (version 7.0.26) (Kumar, Stecher, and Tamura 2016).

The MEME method was used to assess the selection pressures acting separately on the *VP1* and *3D* coding regions of CVA16 worldwide (Murrell et al. 2012), with *P* < 0.05, and the ratio of nonsynonymous to synonymous substitutions (dN/dS) in different datasets >1, revealing positive selection sites.

### 2.3 RF analysis of CVA16

All near-full-length sequences of CVA16 were scanned, and potential recombination events were identified. Briefly, the RDP4 (version 4.95) was used to detect the recombinant breakpoints for the 345-dataset. The *3D* and *VP1* coding regions of CVA16 were extracted from the full-length sequences to construct maximum-likelihood phylogenetic trees, respectively, which suggested a different topology of the phylogenetic trees when the prototype of CVA16 was re-rooted. The genotype and RFs of CVA16 were connected based on their tip names, which reveal the correlation between RF and genotype evolution. The names of RFs were identified according to the original date of evolutionary clusters, and were reported before (Zhang et al. 2010; Han et al. 2020). SimPlot software (version 3.5.1) was used for homology and bootscanning inferences with a 200-nucleotide sliding window moving in 20-nucleotide steps (Salminen et al. 1995). The evolutionary and phylodynamic characteristics of the different RFs were assessed using the Bayesian inference method, which has been stated below. To infer the genetic diversity among the RFs, the mean genetic distance was calculated using the Kimura 2-parameter model with 1,000 bootstrap replicates.

### 2.4 Phylogeography transmission of CVA16

The global CVA16 spatial transmission patterns were inferred using the *VP1* sequence dataset. The type of specimen and isolation region were coded as discrete states, whereas the asymmetric substitution model was used to infer asymmetrical transmission situations between any pairwise regional states by running Bayesian Stochastic Search Variable Selection (BSSVS) (Lemey et al. 2009). The Bayes factor (BF) reveals the significance of the migration pathways, which are summarized as BF >3 and posterior probability (PP) >0.5. Markov jump counts were used to determine the counts of migration in and out of each region, whereas the Markov reward values coded and implemented in BEAST software were used to reconstruct the evolution of CVA16 over time in a region (Minin and Suchard 2008). To increase the accuracy of the transmission links among different countries, four datasets were extracted for independent runs, comprehensively covering phylogenetic clusters and chronological and geographical distributions.

To assess the CVA16 demographic dynamics in terms of RFs and genotypes, a coalescence-based Gaussian Markov random field (GMRF) method with a time-aware smoothing parameter was used (Minin, Bloomquist, and Suchard 2008). GMRF skyride plots were summarized and visualized using Tracer software (version 1.7.1). For each dataset, eighteen individual analyses were combined using one nucleotide substitution model, three different clock models, and six different coalescent tree priors. The Yang96 substitution model was used to estimate the transition and transversion rates, which used GTR with gamma site heterogeneity and partitioned the codons into three partitions.

In the Bayesian inference method implemented in BEAST (version 1.10.4), the nucleotide substitution model of GTR + G4 supported by ModelFinder (Kalyaanamoorthy et al. 2017; Suchard et al. 2018), the uncorrelated relaxed clock and six different coalescent tree priors were used. The sequence collection times were used to calibrate the molecular clocks in the datasets. A total of 200,000,000 generations were used for each analysis, with sampling every 20,000 generations. The time signals of the datasets were checked through Bayesian evaluation of the temporal signal (BETS) and the root-to-tip method used in TempEst (version 1.5.3) (Rambaut et al. 2016; Duchene et al. 2020). The convergence and effective sample size (>200) of the parameters were evaluated using Tracer software (version 1.7.1) (Rambaut et al. 2018). The maximum clade credibility (MCC) trees were summarized using Tree Annotator (version 1.10.4), with a burn-in of the first 10 per cent of the sampled trees and calculation of posterior probability values at each node. The ggtree (version 1.16.3) was used to manipulate the phylogenetic tree, and R software (version 4.2.1) was used to determine the posterior probability value, reroot the tree, and color the branches (Yu et al. 2018). Path sampling (PS) and stepping stone sampling (SS) analyses, with 1,000,000 generations and 100 path steps, were used to reveal the marginal likelihood estimation and choose the most appropriate parameters for the Bayesian phylogenetic models (Baele et al. 2012).

### 2.5 Population structure dynamics of CVA16

The population structures of different RFs and CVA16 genotypes were estimated using the discriminant analysis of the principal components (DAPC) method implemented in the adegenet package (Jombart, Devillard, and Balloux 2010; Jombart and Ahmed 2011). The number of clusters of genetically related individuals was inferred, and the CVA16 population structure features were analyzed in terms of RFs and genotypes. To assess the correlation between the types of clinical specimens and genotype evolution, the association index (AI), parsimony score (PS), maximum monophyletic clade statistics, and phylogeny–trait association analysis were computed using BaTS software (version 2.0) (Parker, Rambaut, and Pybus 2008). *P* < 0.05 was considered to indicate a significant difference.

## 3. Results

### 3.1 Construction of CVA16 sequences dataset

**Figure 1.**
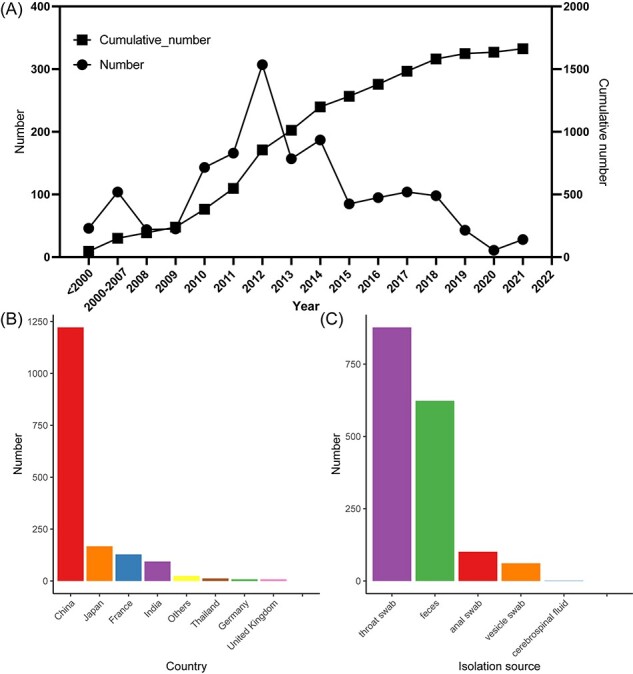
Trend in variation in 1,663 CVA16 sequences dataset in this study. (A) Number of CVA16 sequences during different periods and alternative trends. Circles represent number of CVA16 sequences corresponding to left vertical coordinates. Rectangles represent cumulative number of CVA16 sequences corresponding to right vertical coordinates. (B) Geographic distribution of CVA16 sequences across several countries. (C) Number of CVA16 sequences from different isolation sources.

From the GenBank database, 7,831 CVA16 sequences were collected, and 4,377 sequences with a *VP1* longer than 891 nucleotides were included (Fig. S1). A maximum likelihood tree was constructed, and several evolutionary branches were observed (Fig. S2A). Sequences labeled with the type of specimen, collection date, and isolation region were extracted to create a dataset of 1,663 sequences containing the entire *VP1* coding region (1,663-sequence dataset) (Fig. S1; Fig. S2A; red). These sequences were collected from 18 countries and were mostly from China, followed by Japan, France, India, Thailand, and others (Fig. 1). Most sequences were collected after 2009 (85.6 per cent, 1,424/1,663 from 2010 to 2021), reaching a peak in 2012. Two types of specimens, throat swabs and feces, accounted for 90.2 per cent of these sequences (Fig. 1C).

Then, a new dataset with 442 sequences (442-sequence dataset), covering 18 countries from 1951 to 2021, was subsampled from the 1,663-sequence dataset for genotyping of CVA16 and building the global temporal–spatial transmission pattern (Figs. S1 and S2B; red). To infer the correlation between RF shifts and genotype evolution, 345 near-full-length sequences of CVA16 (345-sequence dataset) with *VP1* and *3D* coding regions were included, of which 84.1 per cent (*n* = 290) were identified after 2009, with the highest number found in 2018 (Figs. S1 and S3).

### 3.2 CVA16 genotype and genetic diversity

**Figure 2.**
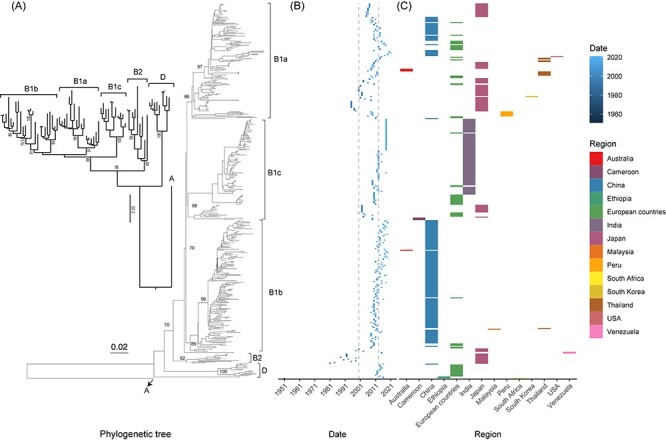
Phylogenetic relationship constructed using genome sequences together with collection date and regions. (A) Neighbor-joining phylogenetic tree based on entire *VP1* gene of 442 sequences. Numbers at major nodes show bootstrap values constructed using neighbor-joining model with 1,000 bootstrap replicates. Inset phylogenetic tree represents genotypes circulating globally. (B) Collection date and (C) geographic distribution of CVA16 along with phylogenetic tree.

The neighbor-joining phylogenetic tree based on the 442-sequence dataset of the *VP1* coding region sequences revealed three genotypes (A, B, and D), two subgenotypes of genotype B (B1 and B2), and three clusters of subgenotype B1 (B1a, B1b, and B1c) (Fig. 2A). The sequences were evenly distributed throughout the phylogenetic tree. Genotype C was not identified based on the *VP1* gene, which is commonly accepted in CVA16 (Mao et al. 2014; Baggen et al. 2018). In total, 92.1 per cent (407/442) of the sequences were collected after 2000, of which 30.3 per cent (134/442) were collected after 2013 (Fig. 2B and Fig. S4). A large proportion of sequences were from China (40.3 per cent, 178/442), covering most evolutionary branches of the phylogenetic tree. Genotype A includes only G10, the prototype strain of CVA16, which was identified in South Africa in 1951. In the phylogenetic tree, the subgenotype B1 of genotype B was the dominant genotype (Fig. 2A). Subgenotype B2, which was prevalent from 1981 to 2000, has a genetic distance of 10.4 per cent–28.9 per cent from other genotypes and has not been sequenced since 2000 (Fig. 2A). The results of further analysis revealed that three clusters (B1a–B1c) of subgenotype B1 with 6.6 per cent–8.0 per cent genetic distance compared with each other circulated globally, and cluster B1b was the leading cluster during the transmission of CVA16. Genotype D is an emerging genotype that formed a novel evolutionary branch with 15.6 per cent–30.6 per cent genetic distance compared with other genotypes.

The association index (AI) and parsimony score (PS) showed that CVA16 *VP1* sequences were more likely to cluster together according to their geographic distribution in the phylogeny tree at a significance level of *P *< 0.001 (Table 1). Although sequences from several countries (e.g. Spain, South Korea, Malaysia, Austria, Ethiopia, Venezuela, and the USA) did not show a strong phylogeny–trait association for the maximum monophyletic clade (MC) due to limited numbers, sequences from Japan, France, China, Cameroon, Thailand, Peru, India, Germany, Australia, and the United Kingdom presented a high association (MC, *P* < 0.05) and clustering.

**Table 1.** Analysis of geographic structure of genetic diversity of CVA16 sequences.

Statistic	Sequence number	Observed mean (95% HPD)	Null mean (95% HPD)	Significance
AI		3.19 (2.62, 3.81)	37.63 (35.34, 39.71)	0^***^
PS		41.34 (41, 42)	236.12 (228.4, 241.96)	0^***^
MC (Japan)	86	16.72 (13, 24)	2.43 (2, 3.22)	0.00999^**^
MC (Spain)	NA	NA	NA	NA
MC (France)	42	12 (12, 12)	1.8 (1.04, 2.7)	0.00999^**^
MC (China)	178	36.81 (35, 51)	3.96 (3.05, 5.11)	0.00999^**^
MC (South Korea)	NA	NA	NA	NA
MC (Cameroon)	3	3 (3, 3)	1 (1, 1)	0.00999^**^
MC (Thailand)	10	4.99 (5, 5)	1.02 (1, 1.08)	0.00999^**^
MC (Peru)	6	5.98 (6, 6)	1.02 (1, 1)	0.00999^**^
MC (Malaysia)	NA	NA	NA	NA
MC (India)	86	15.86 (15, 17)	2.39 (2, 3.09)	0.00999^**^
MC (Austria)	NA	NA	NA	NA
MC (Germany)	8	2 (2, 2)	1.02 (1, 1.01)	0.01999*
MC (Australia)	4	3 (3, 3)	1 (1, 1)	0.00999^**^
MC (Ethiopia)	NA	NA	NA	NA
MC (Venezuela)	NA	NA	NA	NA
MC (United Kingdom)	8	3.7 (3, 5)	1.05 (1, 1.37)	0.00999^**^
MC (USA)	NA	NA	NA	NA
MC (South Africa)	NA	NA	NA	NA

Observed mean, observed value from BaTS analysis; null mean, null (expected) value from BaTS analysis; NA, no data available due to insufficient sample size (*n* < 3).

Significance thresholds.

* 0.01 < *P* < 0.05.

** 0.001 < *P* < 0.01.

*** *P* < 0.001.

### 3.3 Global evolutionary dynamics of CVA16

**Figure 3.**
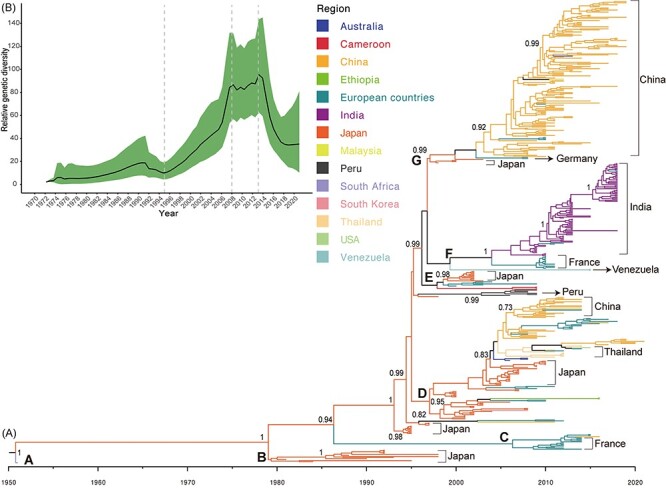
Temporal phylogenies and evolutionary characteristics of CVA16 based on *VP1* coding regions worldwide. (A) MCC phylogenetic tree based on *VP1* coding regions, with different colors representing different countries. Numbers at major nodes indicate posterior support. Clusters A–G are labeled to display evolutionary clades. (B) Four phases are shown in global relative genetic diversity of *VP1* coding regions of CVA16. Y-axis shows the measure of genetic diversity (logarithmic scale of Neτ). The range represents 95 per cent credibility interval; lines indicate median values of CVA16 population size.

On the basis of 442-sequence dataset collected worldwide, seven major evolutionary clusters (A–G) of the *VP1* coding region were observed in the MCC tree along the temporal scale, with the phylogenetic tree showing a typical ladder-like topology of and a gradual evolutionary process for CVA16 (Fig. 3A). Sequences from clusters A and B were absent in more recent sequencing studies. Several sequences collected from several countries such as China, Thailand, and Japan were aggregated within Cluster D. Clusters F and G primarily consisted of numerous sequences identified from India and China, respectively.

According to the sequences of the *VP1* coding region, the global genetic evolution of CVA16 can be divided into four stages (Fig. 3B): the low genetic diversification stage before 1995, the rapid increasing genetic diversification stage from 1995 to 2008, the genetic diversity fluctuation stage from 2008 to 2013, and the decline in genetic diversity stage after 2013. Except for the sequences reported in 2003, the others were closely related to the time scale, and some sequences collected in adjacent years were mixed (Fig. S5 A–B). The results of population clustering revealed a significant division of genotypes (Fig. S5 C), in which there were two high-frequency variable loci (Fig. S5 D). Single-nucleotide polymorphism (SNP) loci 126 and 594 were transferred from nucleotide C to U after 2006, and high-frequency variable loci fluctuated with time (Fig. S6 D–E).

### 3.4 Global spatial transmission patterns of CVA16

**Figure 4.**
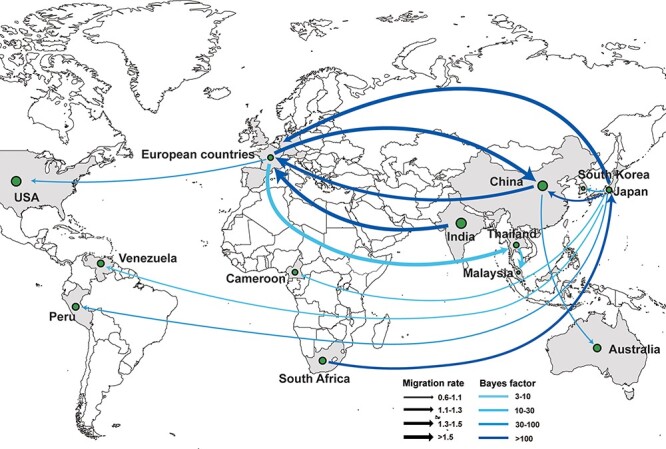
Spatial transmission routes of CVA16 based on Bayesian phylogeographic inference. Different transmission pathways are represented by curves ending in arrows that connect different regions (statistically supported by BF >3 and PP >0.5). Line color indicates supportive value of the BF; line thickness represents the distinct migration rate.

The global spatial transmission dynamics of CVA16, inferred according to the *VP1* coding region, included 20 decisive migration pathways with significant BF and PP support (BF >3 and PP >0.5). Among these, the migration pathways in Europe, Japan, and China played a decisive role in seeding global CVA16 transmission, followed by Thailand and India (Fig. 4 and Fig. S7). In agreement with the seeding status, the Markov reward values in China, France, India, Japan, and Germany were significantly higher than those in the other countries (Fig. S8 A). CVA16 (G-10) was first collected in South Africa in 1951 and was next detected in Japan (BF = 197.82), followed by China (BF = 541.38), Germany (BF = 48,873.63), Cameroon (BF = 27.71), and South Korea (BF = 40.71). Finally, the virus spread to peripheral countries and around the world (Fig. 4 and Fig. S7).

To confirm the accuracy of the transmission links, four independent datasets were extracted from the 1,663-sequence dataset. The results revealed eight common spatial migration pathways with strong supporting evidence (BF >100), supporting the stability of the significant global spatial transmission patterns (Fig. S9). Higher viral migration rates were identified between relatively close countries, such as Germany to France (migration rate = 2.2358) and France to the United Kingdom (migration rate = 1.7334), as well as between two countries (e.g. Japan to Germany, migration rate = 2.078) separated by a relatively large distance (Fig. 4 and Fig. S7). Fitting the nonlinear regression correction, a weak inverse trend was found between geographic distance and mobility or posterior probabilities, although some long-distance movements occurred, which indicated that viral movements preferably occurred at shorter distances (Fig. S8 C–D).

### 3.5 RFs of CVA16

**Figure 5.**
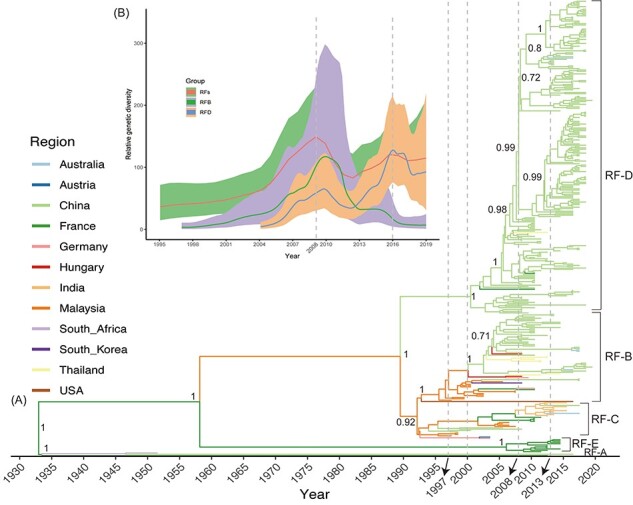
Temporal phylogenies and evolutionary characteristics of CVA16 based on *3D* coding region. (A) MCC phylogenetic tree based on the *3D* coding region, which are colored according to different countries. Numbers at major nodes represent posterior support. (B) Relative genetic diversity of *3D* coding region of CVA16. Y-axis shows the measure of genetic diversity (logarithmic scale of Neτ). Shading in different colors represents the 95 per cent credibility interval; lines indicate median values of CVA16 population size of three datasets (overall data, RF-B, and RF-D).

Before phylogenetic analysis of the RFs, the temporal signals of the 345-sequence dataset were checked (Fig. S10), and 3D sequences containing recombination breakpoints were excluded. Based on the 345-sequence dataset, the MCC tree showed five major evolutionary branches of RFs (RF-A to -E; Fig. 5). Numerous sequences identified in several countries aggregated into RF-B or RF-C, whereas several sequences identified in China clustered into RF-D. Most sequences that gradually evolved from early branches and filled the lineages of the MCC tree in a ladder-like topology showed the typical co-circulation of multiple lineages over time (Fig. 5A). A shift from RF-B to RF-D was observed over time and contributed to the changes in relative genetic diversity (Fig. 5B). In the process of their transmission, the genetic diversity of RFs overall peaked in 2008 and 2016 and reached a nadir in 2012. RF-B played a substantial role in genetic diversity before 2012, then RF-D played the similar role after 2012.

The molecular clock, assessed through Markov chain Monte Carlo (MCMC) analysis on the basis of the 345-sequence dataset containing *VP1* and *3D* coding regions, revealed that the evolutionary rate of the *3D* coding region was 4.09 (95 per cent highest probability density (HPD), (3.77–4.45)× 10^−3^) substitutions per site per year, higher than that of the *VP1* coding region at 3.54 (95 per cent HPD, (3.13–3.99)× 10^−3^) substitutions per site per year (Table 2). The most recent common ancestor (MRCA) of genotype D was dated to August 2002 (95 per cent HPD, September 1996–October 2007), which is earlier than that of RF-E, tracked to April 2005 (95 per cent HPD, January 2002–June 2008), whereas MRCA of genotype A (August 1931 (95 per cent HPD, February 1926—February 1937)) was similar to that of RF-A (September 1932 (95 per cent HPD, June 1927–May 1937)). The finding was similar for MRCA of genotype B1b, dated to June 1998 (95 per cent HPD, May 1993–April 2002), which was earlier than that of RF-D (October 1999 (95 per cent HPD, May 1997–February 2002)). The periodical duration of RF replacement commonly occurred every 6–8 years (Fig. S11).

**Table 2.** Summary of MRCA, substitution rates, and other parameters obtained through MCMC analysis.

			Divergence*^c^*			MCMC (BEAST)*^e^*	
Coding region*^a^*	RFs/Genotypes*^b^*	*n*	Nucleotide	Amino acid	dN/dS*^d^*	Substitution rate (10^−3^) (95% HPD)	TMRCA (95% HPD)
	Genotypes						
VP1	RF-A (A, B1a, and B1b)	345	0.235	0.056	0.00854	3.54 (3.13–3.99)	1931/08 (1926/02–1937/02)
	RF-B (B2, B1a, and B1b)		0.063	0.005	0.0159		1989/01 (1983/08–1993/08)
	RF-C (B1a, B1b, and B1c)		0.065	0.011	0.0291		1989/01 (1984/03–1993/04)
	RF-D (B1b)		0.051	0.007	0.0343		1998/06 (1993/05–2002/04)
	RF-E (D)		0.047	0.004	0.0265		2002/08 (1996/09–2007/10)
	RFs						
3D	RF-A	345	0.241	0.048	0.0102	4.09 (3.77–4.45)	1932/09 (1927/06–1937/05)
	RF-B		0.065	0.018	0.0611		1992/02 (1989/05–1994/08)
	RF-C		0.065	0.02	0.086		1991/11 (1989/03–1994/04)
	RF-D		0.051	0.016	0.0803		1999/10 (1997/05–2002/02)
	RF-E		0.038	0.007	0.0503		2005/04 (2002/01–2008/06)

a Coding region used in overall dataset.

b Dataset dividing criteria based on *VP1* and *3D* coding regions.

c Overall mean distances using Kimura 2-parameter model.

d dN/dS ratios for each dataset using MEME method, with significance value of *P* < 0.05.

e Value is based on MCMC analysis of each dataset.

### 3.6 Association between RF replacement and evolutionary dynamics of CVA16 genotypes

**Figure 6.**
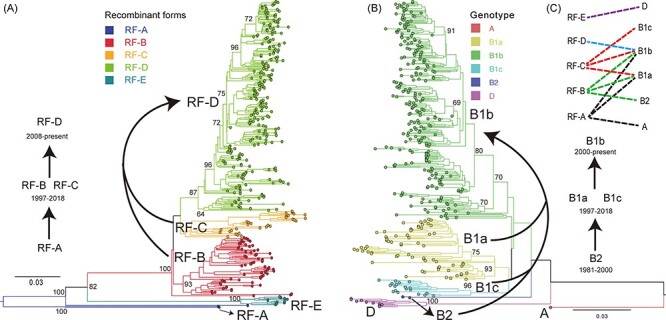
Evolutionary profile of RFs and genotypes of CVA16 worldwide. Neighbor-joining phylogenetic tree based on (A) *3D* coding region of 345 CVA16 sequences and (B) *VP1* coding region of corresponding CVA16 sequences. (C) Correlation between RF replacement and genotype evolution between RFs and genotypes, and dashed lines indicate RF–genotype association, which are also shown in Figure S7B. Numbers at major nodes show bootstrap support values, with 1,000 bootstrap replicates. Arrows indicate evolutionary trends in RFs and genotypes.

When the evolution of *3D* was related to that of *VP1* (Fig. 6 and S12), the sequences belonging to RF-D in the *3D* phylogenetic tree were clustered only with B1b (genotype B) in the *VP1* phylogenetic tree, and the sequences of RF-E clustered only with those of genotype D. However, the sequences of RF-A were clustered with those of genotypes A, B1a, or B1b; the sequences of RF-B clustered with those of genotypes B2, B1a, or B1b; and sequences of RF-C clustered with those of genotypes B1a, B1b, or B1c (Fig. 6C and S12). The prototype sequence of CVA16 from an isolate from 1951 was classified as genotype A and this genotype has not been detected since (Fig. 6). In recent years, the emergence of genotype D was accompanied by the appearance of RF-E (Fig. 6 and Table 2). The complex relationships between RF-B or RF-C with B2, B1a, B1b, and B1c are shown in Fig. 6C and S12. The shifts from the early cocirculation of RF-B and RF-C to the late circulation of RF-D were accompanied by changes owing to the early circulation of B2 to the cocirculation of B1a and B1c and then to B1b (Fig. 6 and S12). Although RF-D was the main RF, cluster B1b dominated in the CVA16 epidemics worldwide.

**Figure 7.**
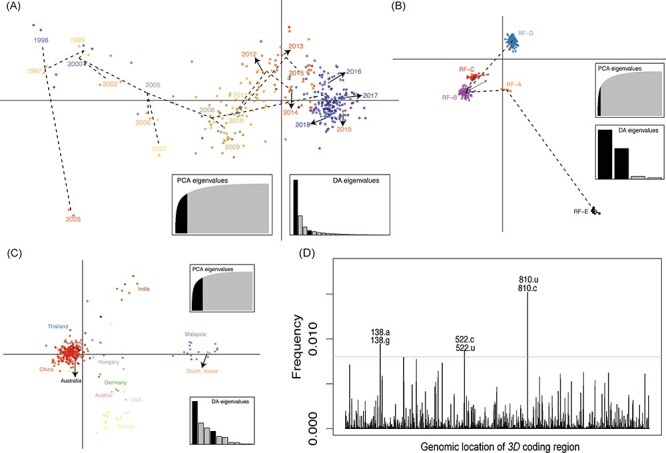
Principal components of CVA16 sequences with geographic region, collection date, and RFs used as prior groups based on *3D* coding region. Different colors represent prior groups, and individual genomes are marked as dots. Results of eigenvalues analysis (PCA and DA) displayed in the inset. Axes represent the first two principles. Scatterplots using (A) collection date, (B) RFs, (C) geographic distribution as prior clusters. (D) High allele frequency and genomic locations in *3D* coding region. PCA, principal components analysis; DA, discriminant analysis.

Except for the sequences of the *3D* coding region detected in 2003, the sequences were uniformly distributed on the first principal component along the time scale, with several members being mixed (Fig. 7A-B). The sequences from Malaysia, South Korea, and India showed mild separation compared with those from other countries. However, the other sequences were mixed, revealing a substantial overlap among the different countries (Fig. 7C). In addition, three high-frequency variable sites were found in the *3D* coding region, and single-nucleotide polymorphism (SNP) sites gradually altered over time, contributing to changes in CVA16 population dynamics (Fig. 7D). Nucleotide A at SNP site 138 dominated in 2003, and then decreased and shifted to G in other years (Fig. S6 A). SNP site 810 shifted from nucleotide U to C after 2008 and fluctuated over time, even though SNP site 522 more frequently transformed (Fig. S6 B-C).

## 4. Discussion

As the major antigenic neutralization site, the *VP1* coding region has been used as a target for EV molecular serotyping, and is currently the gold standard for EV typing (Oberste et al. 1999a, 1999b, 2000). However, the highest EV recombination frequency was observed in the *3D* coding region, which was used to designate RFs and reveal RF replacement (Lukashev 2005; Gaunt et al. 2015; Song et al. 2020). Evolution of the *VP1* coding region and the emergence of RFs in the *3D* coding region of CVA16 may lead to large outbreaks; therefore, further research is warranted. To address this concern, 1,663 sequences from the GenBank database with comprehensive trait information and phylogenetic representation were retrieved in this study to form two major datasets, including a 442-sequence dataset containing the sequences of the *VP1* coding region and a 345-sequence dataset containing the near-full-length sequences of CA16, which were constructed to infer the global phylogeographical transmission dynamics of CVA16 and RF shifts.

The 1,663 sequences covered 18 countries and spanned the years 1951–2021, revealing the large-scale, long-term circulation of CVA16. Genotypes A, B, and D; subgenotypes B1 and B2; and clusters B1a, B1b, and B1c of CVA16 worldwide are typed using the *VP1* coding region, as previously described (Zhang et al. 2010; Hassel et al. 2017). Genotype B1b dominated the global CVA16 epidemic, whereas B2 and A have not been detected since 2000 and 1951, respectively. Although B1a and B1c circulate worldwide, they account for only a low proportion of CVA16 serotypes. The emerging genotype D shows the longest distance from the existing genotypes, forming a new evolutionary branch. The genetic diversity of CVA16 shows four global evolutionary stages, peaking in 2013 (Fig. 3B). The continuous mutations in CVA16 sequences have contributed to its evolution, leading to novel evolutionary branches (Zhang et al. 2010; Hassel et al. 2017; Han et al. 2020). The accumulation of mutations may be a powerful force driving the emergence of new evolutionary branches and even serotypes of EVs (Lukashev 2005; Tan et al. 2016).

Three migration pathways of CVA16 have been observed globally, resulting in the establishment of endemic populations and circulation. Europe, China, and Japan are the main regions from which CVA16 is globally transmitted. Nine migration pathways (BF >100) were identified, particularly for viral activity in Europe. The inverse trend between geographic distance and migration rates suggests that CVA16 easily spreads locally, which is verified by the transmission within Europe. These complicated transmission pathways suggest a variety of migration routes for CVA16, linking the five continents. Increased international travel have promoted the spread of the virus. Most sequences were collected in China after 2000 and covered almost all the evolutionary branches. The inactivated EV-A71vaccine has been available in China since 2016. However, CVA16 remains a frequent cause of HFMD worldwide and requires the rapid development of CVA16 vaccines to control CVA16-related HFMD and extensive vaccine inoculation strategies worldwide, especially in the Asia-Pacific region (Xing et al. 2014).

The sequences were distributed worldwide and collected through diagnostic testing, which provided clues regarding the continuous evolution of CVA16. Geography-driven adaptability is speculated to play an important role in determining the diversity of CVA16. However, we also realize that the clustering of CVA16 global isolates should be cautiously illustrated because of the unbalanced numbers.

The *3D* coding region is an important functional region that produces RNA-dependent RNA polymerase (RdRp) (Baggen et al. 2018). Recombination events frequently occur in the *3D* coding region and are recognized as the main mechanism through which CVA16 evolves (Lukashev 2005; Kyriakopoulou et al. 2015; Tan et al. 2016). Five CVA16 RFs were identified in this study, which revealed five distinct evolutionary groups. Compared with the other RFs, RF-E is genetically distant from RF-A to RF-D. Regarding genetic diversity, a fluctuating transition pattern from RF-B to RF-D was observed. (Fig. 5B). The cocirculation of RF-B and RF-C during the same period implies that a dominant clade was not formed. Genotypes B1a and B1c also cocirculated during a similar period, which resulted in the emergence of the dominant B1b. Based on this logic, the current dominant clade persistently evolved and result in a new clade, which suggest that comprehensive monitoring of EVs around the world is essential.

The dynamic regulation strategy of *3D* and *VP1* observed in CVA16 produced shifts from RF-B or RF-C to RF-D, which was accompanied by the replacement from the early genotype B2 to B1a and B1c and then to B1b, which confirms the synergy between the evolution of the CVA16 genotype and the RF shift. Additional relationships between the coevolution of *3D* and *VP1* genes and the clinical features of CVA16 may be a concern in the future, requiring more comprehensive and global monitoring. By measuring the approximate half-lives for a single RF, the periodical duration of CVA16 RF replacement occurred approximately every 6–8 years, which is similar to that of EV-A71 (5.9–9.4 years) but longer than that of echoviruses 9 and 30 (1.3–3.1 years) (McWilliam Leitch et al. 2010, 2012).

In conclusion, the results obtained from large-scale and long-term CVA16 sequence datasets confirmed four global oscillating phases of CVA16 evolution, with a peak in 2013, and three global migration pathways of CVA16. Europe, China, and Japan have served as the seeds for the global transmission of CVA16. The *3D* coding region harbors a high recombination frequency, which led to shifts from RF-B and RF-C to RF-D, which was accompanied by the replacement from early genotype B2 to B1a and B1c, and then to B1b, indicating a synergetic evolution strategy of RF shifts and the evolution of CVA16 genotypes.

## Supplementary Material

vead080_Supp
