# Supplementary material for: Synergetic association between coxsackievirus A16 genotype evolution and recombinant form shifts
Source: Virus Evol. 2023 Dec 20;10(1):vead080. doi: 10.1093/ve/vead080 (PMC10868544; doi:10.1093/ve/vead080)
Supplement: vead080_Supp [file vead080_supp.zip › suppl_data/Supplementary Material-20231215.pdf]

Supplementary Material

Figure S1. Workflow of sequences filter strategy in this study. Core datasets are marked in red.

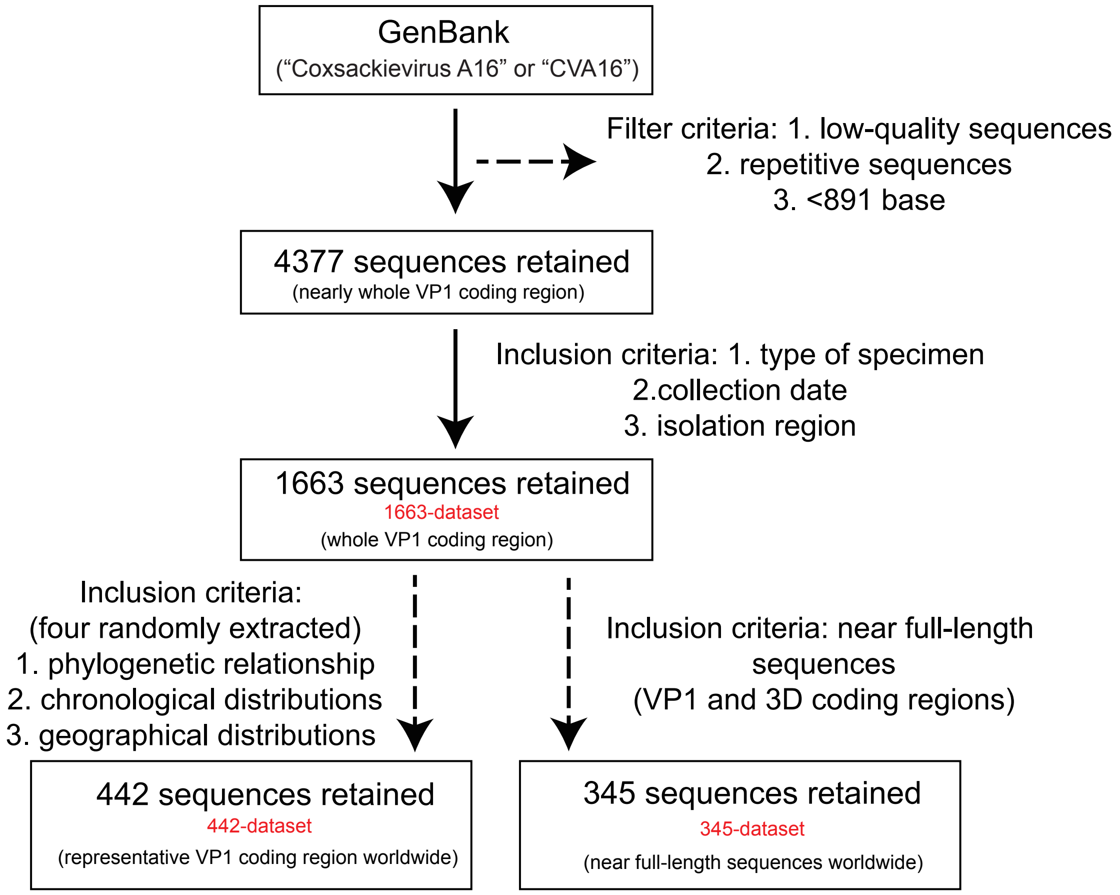

Figure S2. Maximum likelihood phylogenetic tree of CVA16 based on *VP1* coding region. Scale bars indicate substitutions per site per year. Numbers at major nodes indicate bootstrap values, with 1000 bootstrap replicates. Red branches represent genomic sequences selected for the next analysis of phylogenetic inference and RF replacement. Maximum likelihood phylogenetic tree of (A) 4377-sequences and (B) 1663-sequences dataset of CVA16. To reduce possible dataset bias, we performed several random extractions from 1663-sequences dataset, comprehensively covering phylogenetic clusters and chronological and geographical distributions.

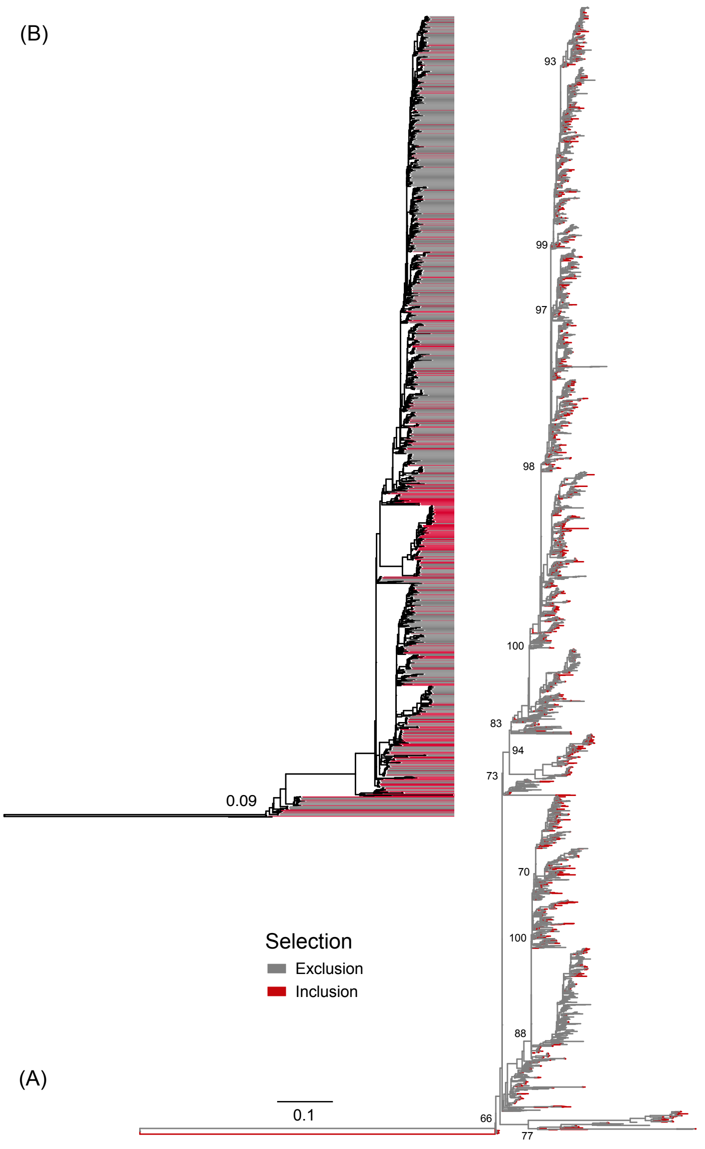

Figure S3. Variation in dataset with 345 near-full-length sequences used to infer RFs and genotypes in this study. (A) Number of CVA16 sequences at different time points. (B) Geographic distribution of CVA16 sequences across several countries. (C) Numbers of several genotypes or subgenotypes of CVA16 sequences. (D) Numbers of different RFs of CVA16 sequences.

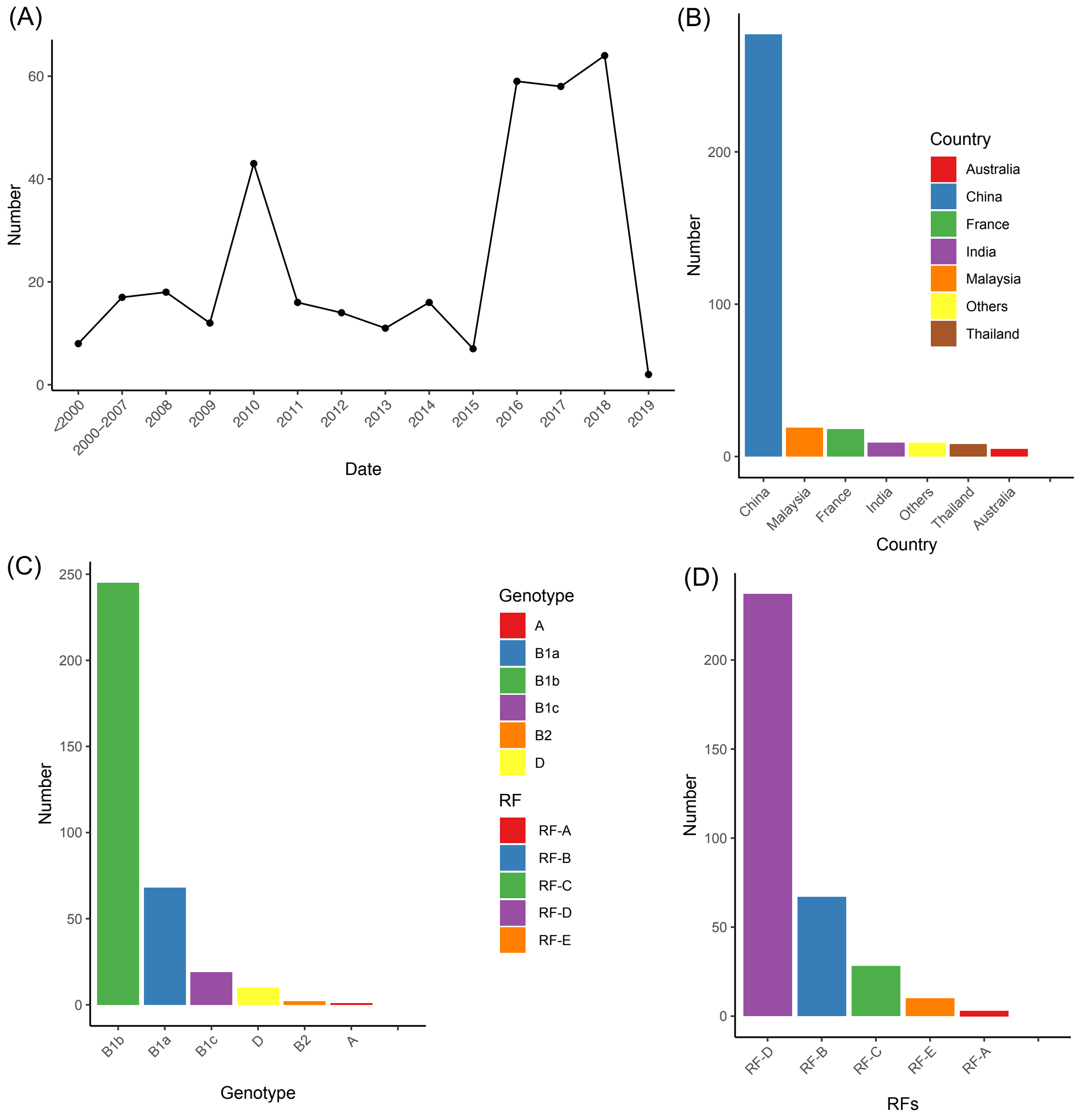

Figure S4. Trend in variation of CVA16 442-dataset in this study. (A) Number of CVA16 sequences during different periods and alternative trends. Circles represent number of CVA16 sequences corresponding to left vertical coordinates. Rectangles represent cumulative number of CVA16 sequences corresponding to right vertical coordinates. (B) Geographic distribution of CVA16 sequences across several countries. (C) Number of CVA16 sequences from different isolation sources.

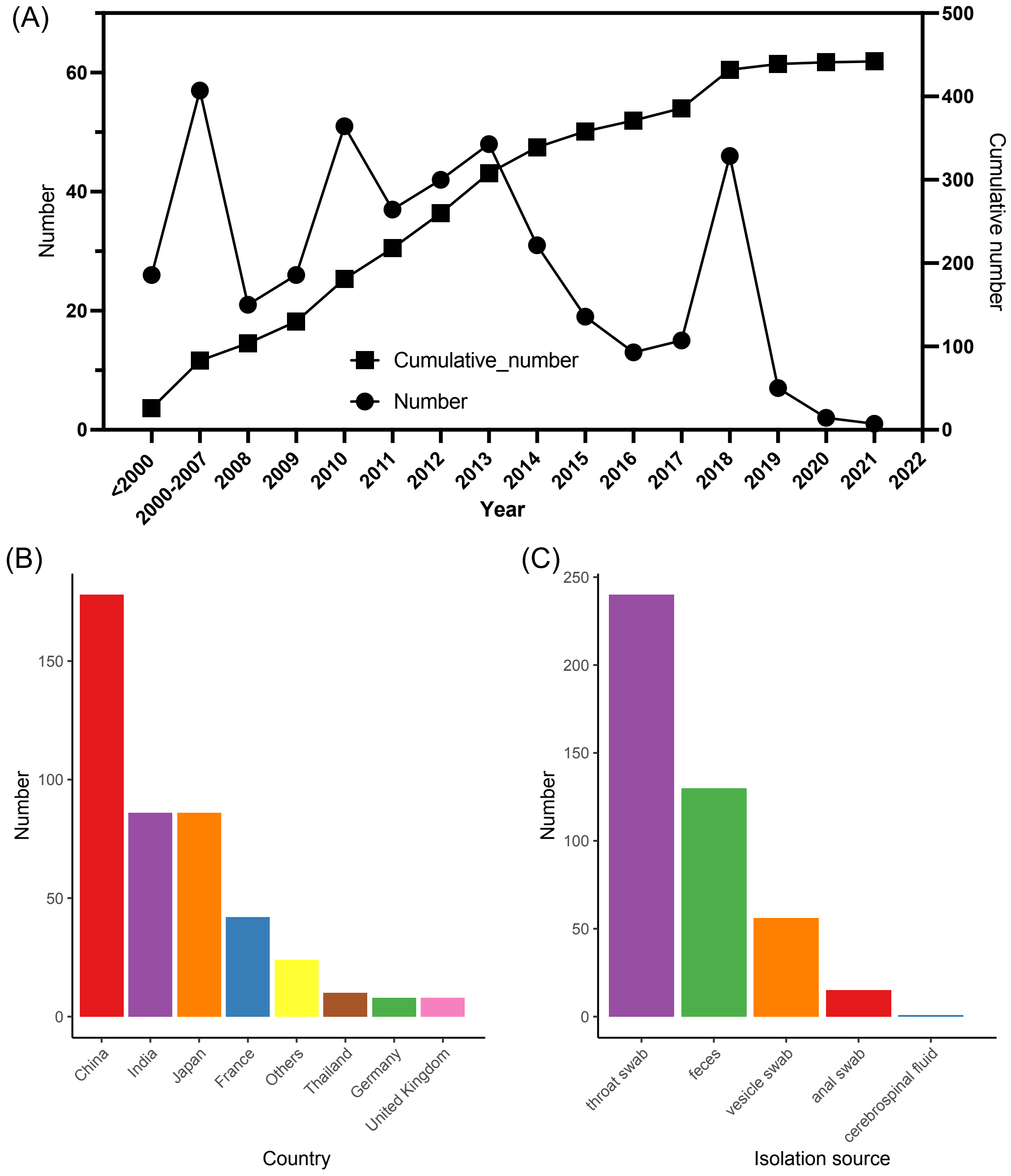

Figure S5. Principal components of CVA16 sequences with collection date and genotypes used as the prior groups based on *VP1* coding region. Different colors represent the prior groups, and individual sequences are marked as dots. Results of eigenvalues analysis (PCA and DA) is displayed in the inset. (A) Scatterplot using collection date as a prior cluster. (B) Subsequent scatterplot of a subset of CVA16 sequences. (C) Scatterplot using the genotypes as a prior cluster. (D) High allele frequency and genomic locations. PCA, principal components analysis; DA, discriminant analysis.

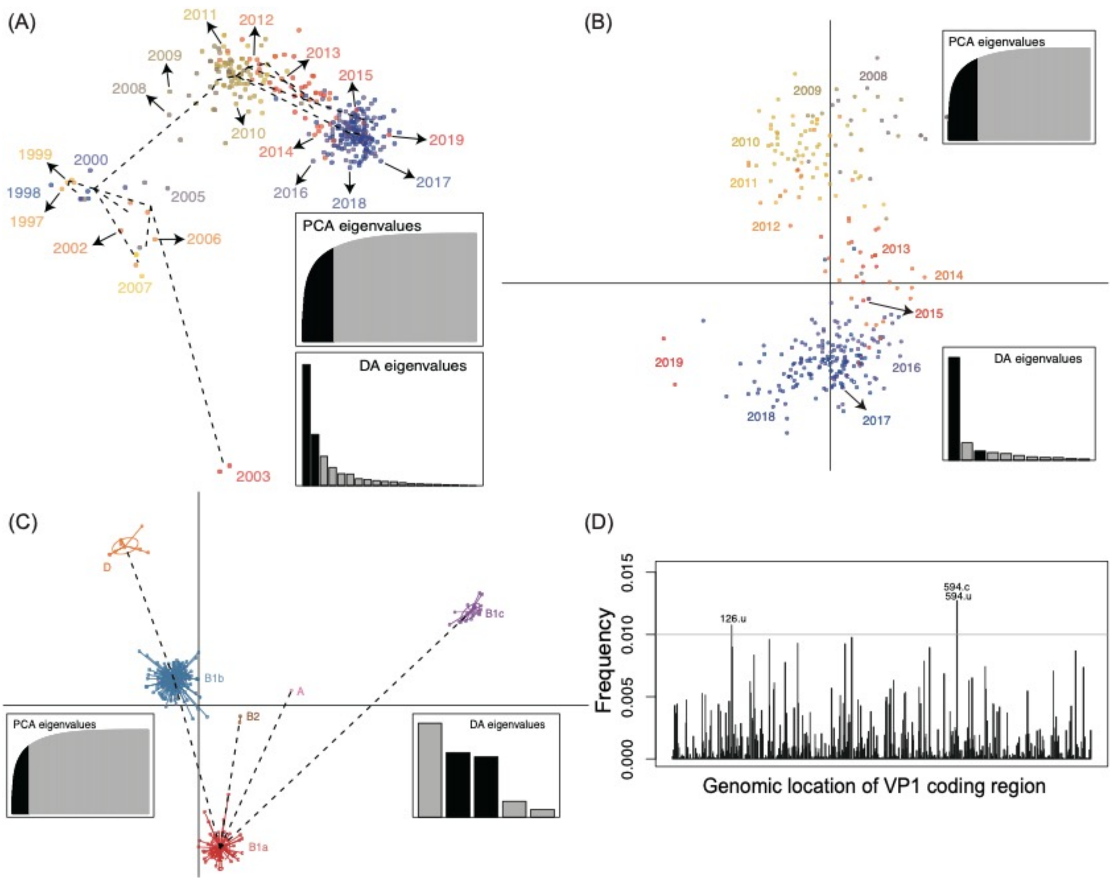

Figure S6. Allele variation frequency in CVA16 over time. Single nucleotide polymorphic (SNP) (A) site 138, (B) site 522, and (C) site 810 in *3D* coding region. SNP (D) site 126 and (E) SNP site 594 in *VP1* coding region.

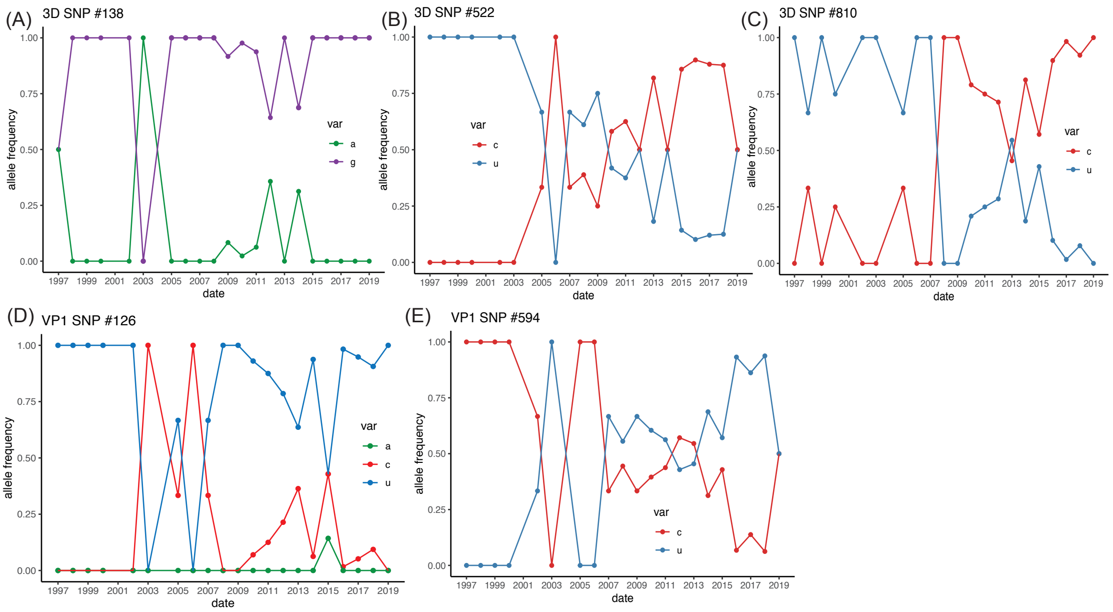

Figure S7. Spatial transmission routes of CVA16 based on Bayesian phylogeographic inference. Different transmission pathways are represented by curves ending in arrows that connect different regions (statistically supported by BF > 3 and PP > 0.5). Line color indicates supportive value of the BF; line thickness represents the distinct migration rate. The transmission routes within the European countries were estimated.

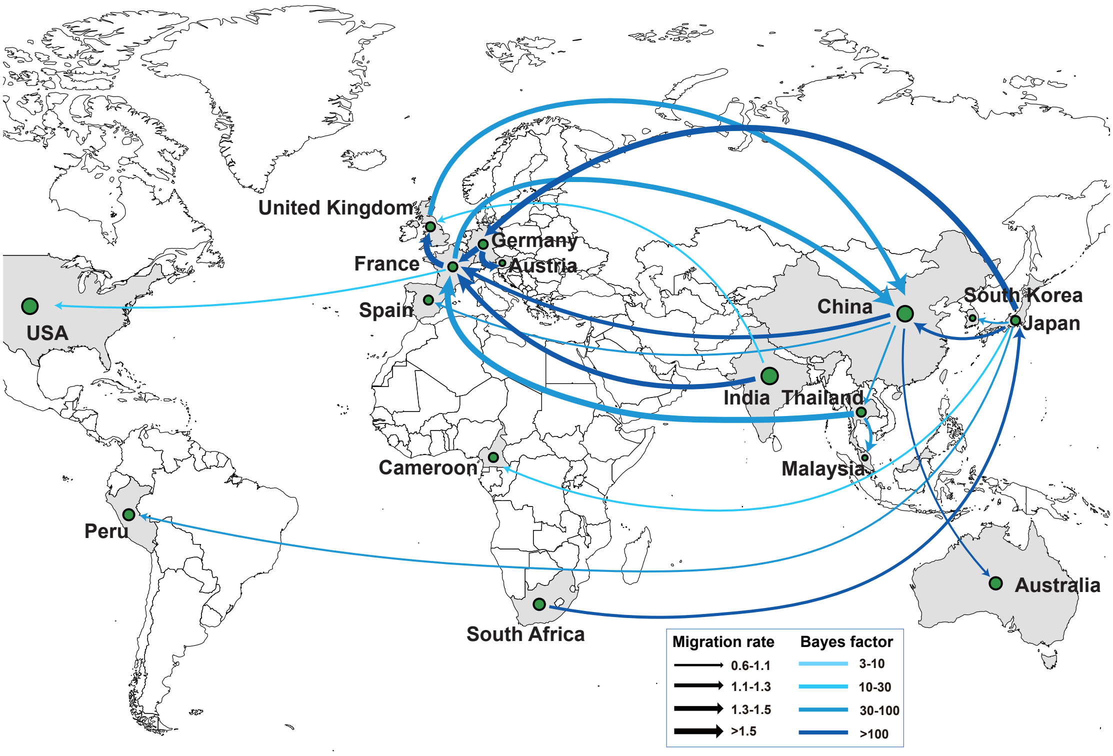

Figure S8. (A) Box plot of Markov reward values; Y axis represents density distribution of total time spent in particular locations. (B) Box plot of Markov reward values; Y-axis represents density distribution of total time spent in different types of clinical specimens. (C) Nonlinear regression correlation among countries' virus transition rates (supported with BF >3 and PP > 0.5, see Materials and Methods) and geodesic distance. (D) Nonlinear regression correlation among countries' posterior probability support (supported with BF >3 and PP > 0.5) and geodesic distance.

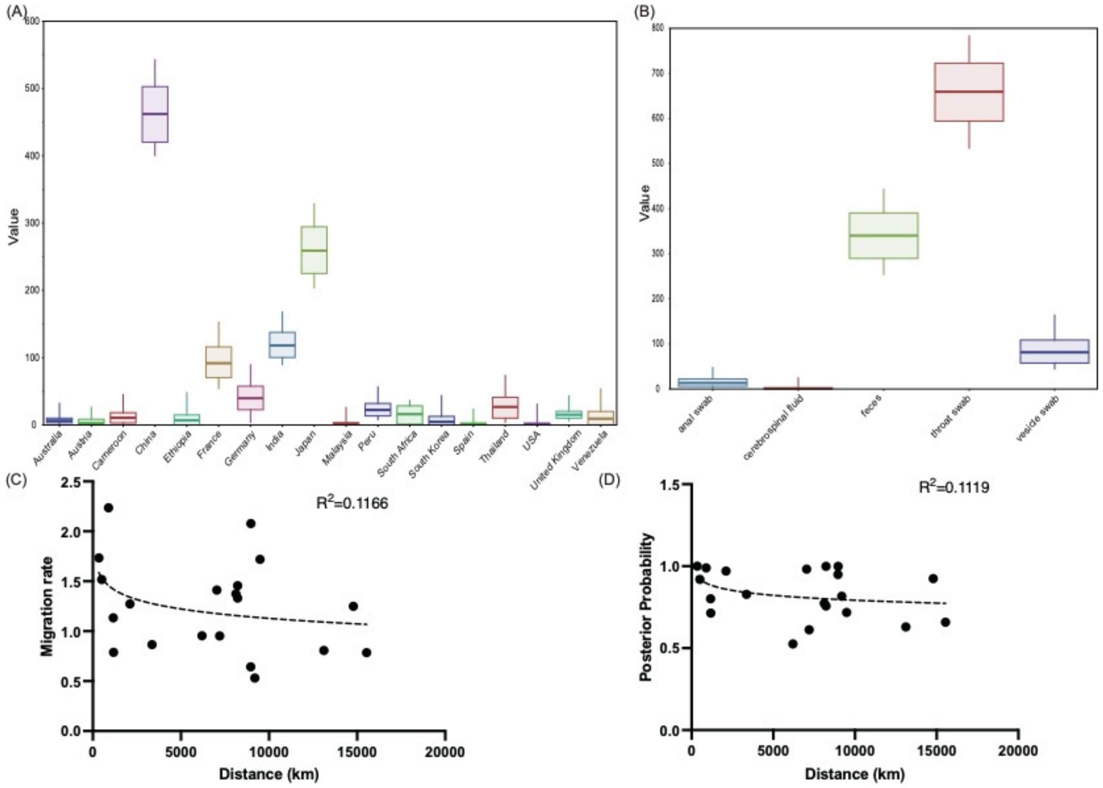

Figure S9. Level of BF support for each transmission route of four independent estimates (A–D). X and Y axes represent origin and destination location, respectively.

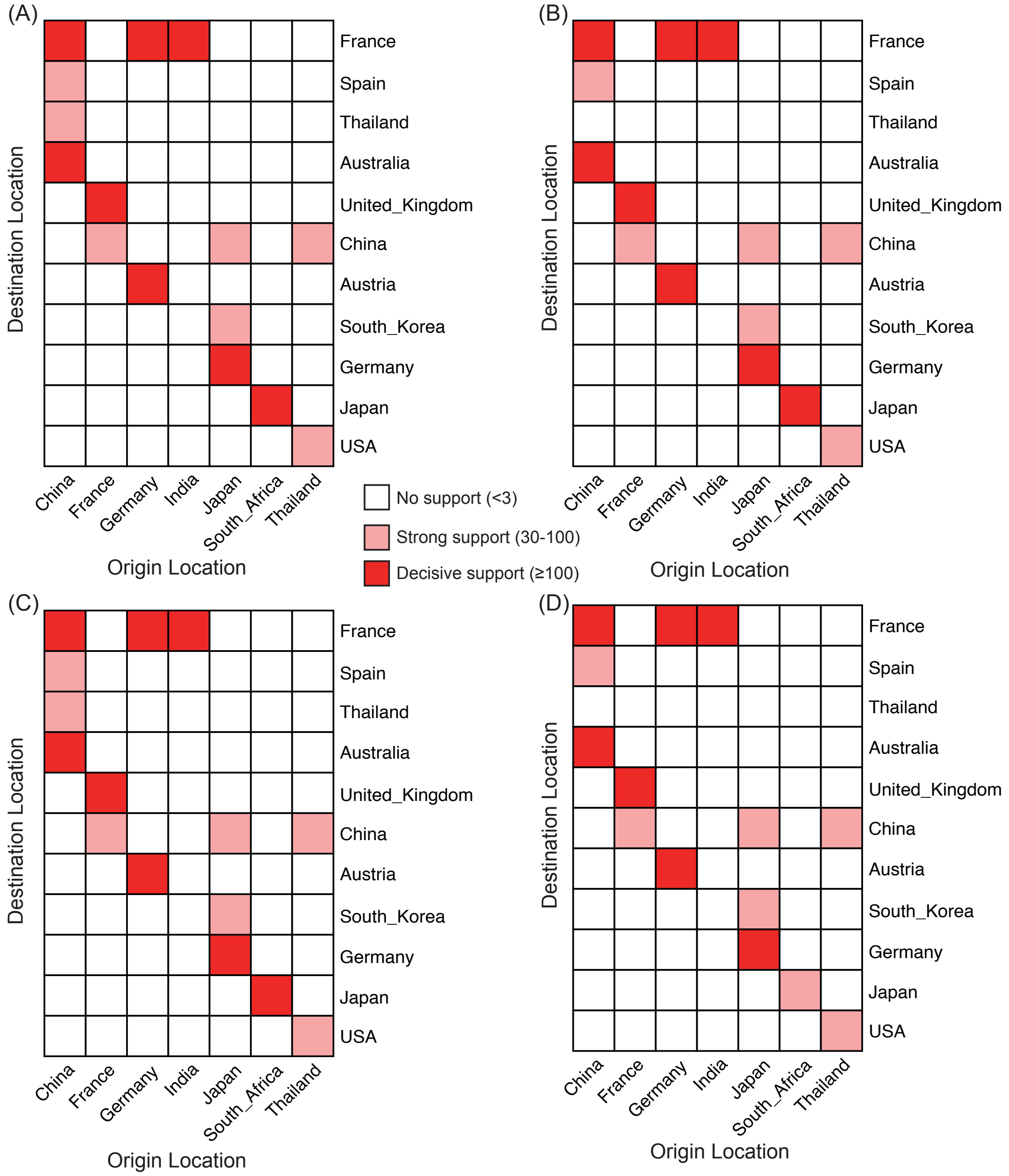

Figure S10. (A) Association between root-to-tip divergence and sampling dates using the *3D* coding region dataset. Results of Bayesian evaluation of temporal signals (BETS) using the (B) *3D* and (C) *VP1* coding region datasets using two methods to estimate for sufficient temporal signals.

(A)

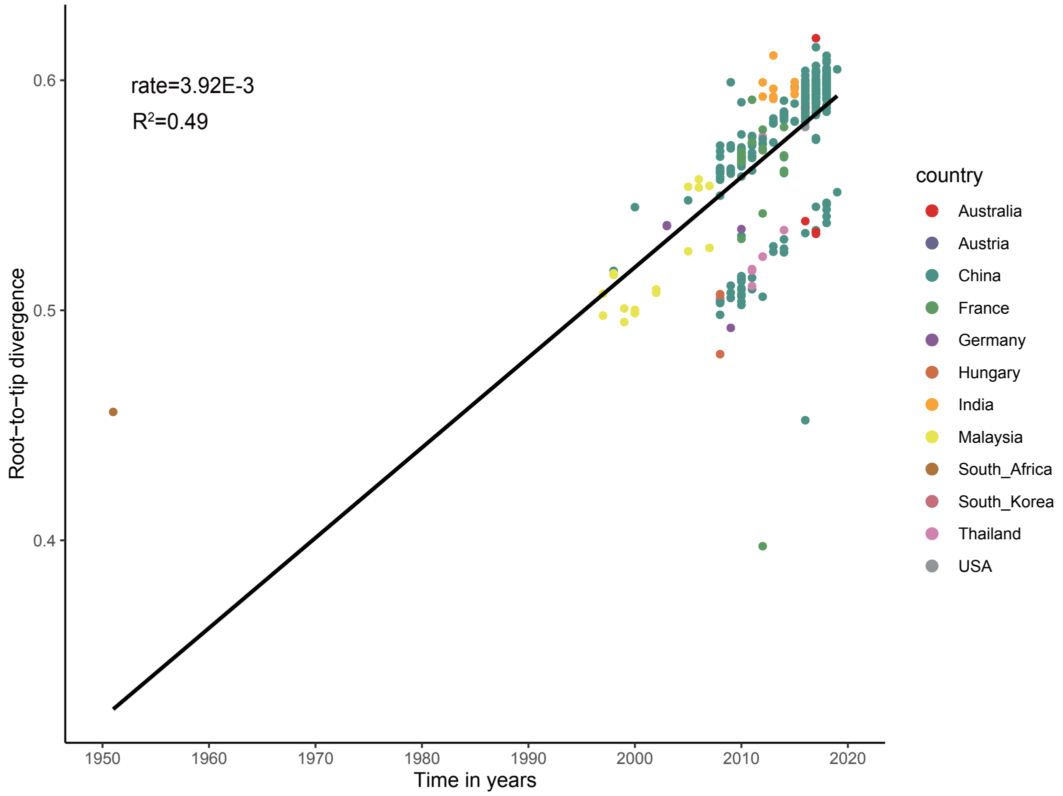

(B)

Data sets name	Molecular clock model	Coalescent tree prior	log marginal likelihood	log BF
3D-345-CTMC.xml	strict clock	constant size	-131045.4	97429
3D-345-tipdate.xml	strict clock	constant size	-33616.02	

(C)

Data sets name	Molecular clock model	Coalescent tree prior	log marginal likelihood	log BF
VP1-442-CTMC.xml	strict clock	constant size	-24941.81	896.27
VP1-442-tipdate.xml	strict clock	constant size	-24045.54	

Figure S11. Kernel density of different RFs based on the estimated MRCA. X axes represent TMRCA distribution. Results of (A) *3D* and (B) *VP1* coding regions.

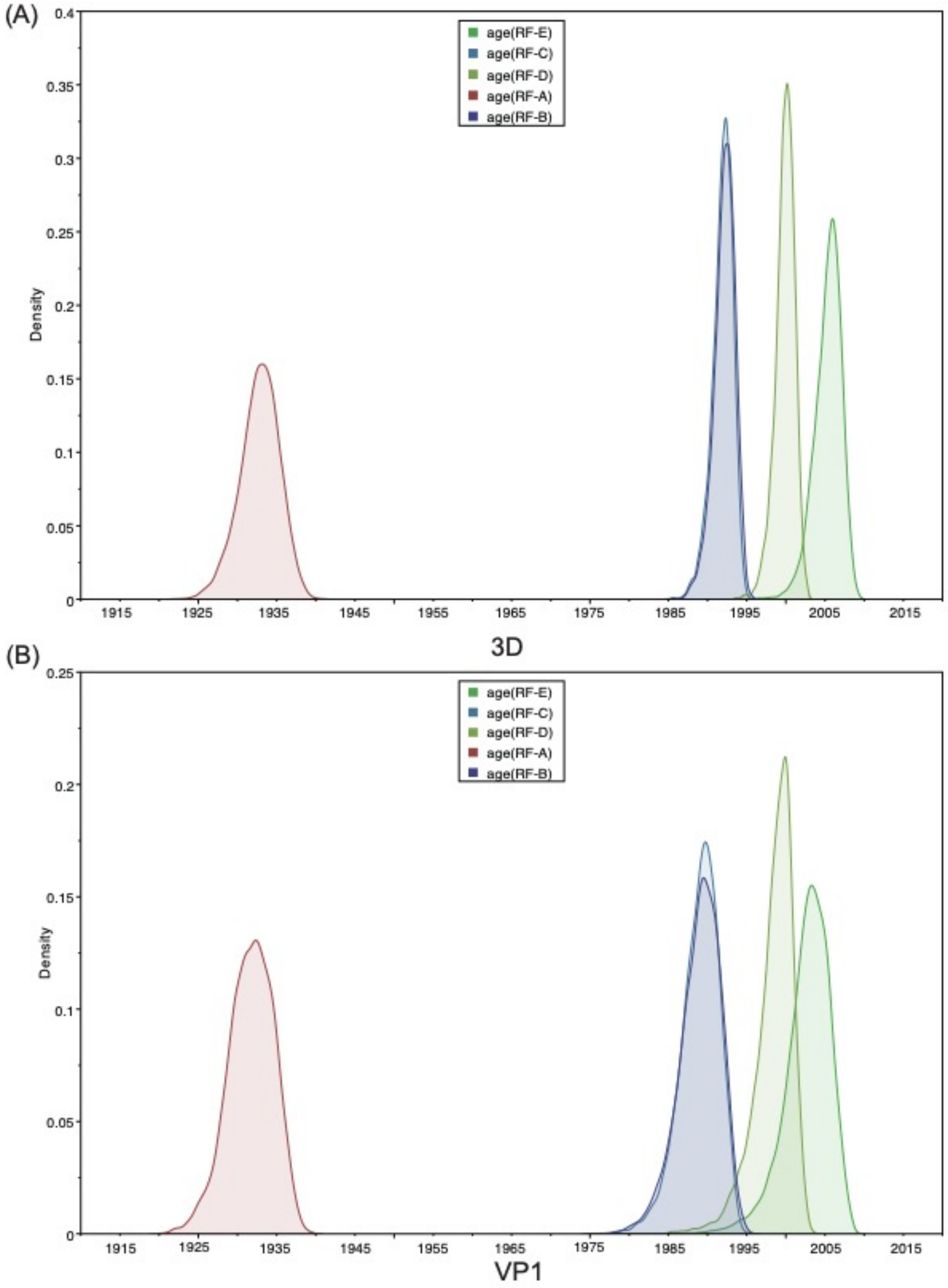

Figure S12. Correlation between RF replacement and genotype evolution based on a subset of 345 sequences of CVA16. Lines indicate RF–genotype association, and different-colored points represent RFs and genotypes.

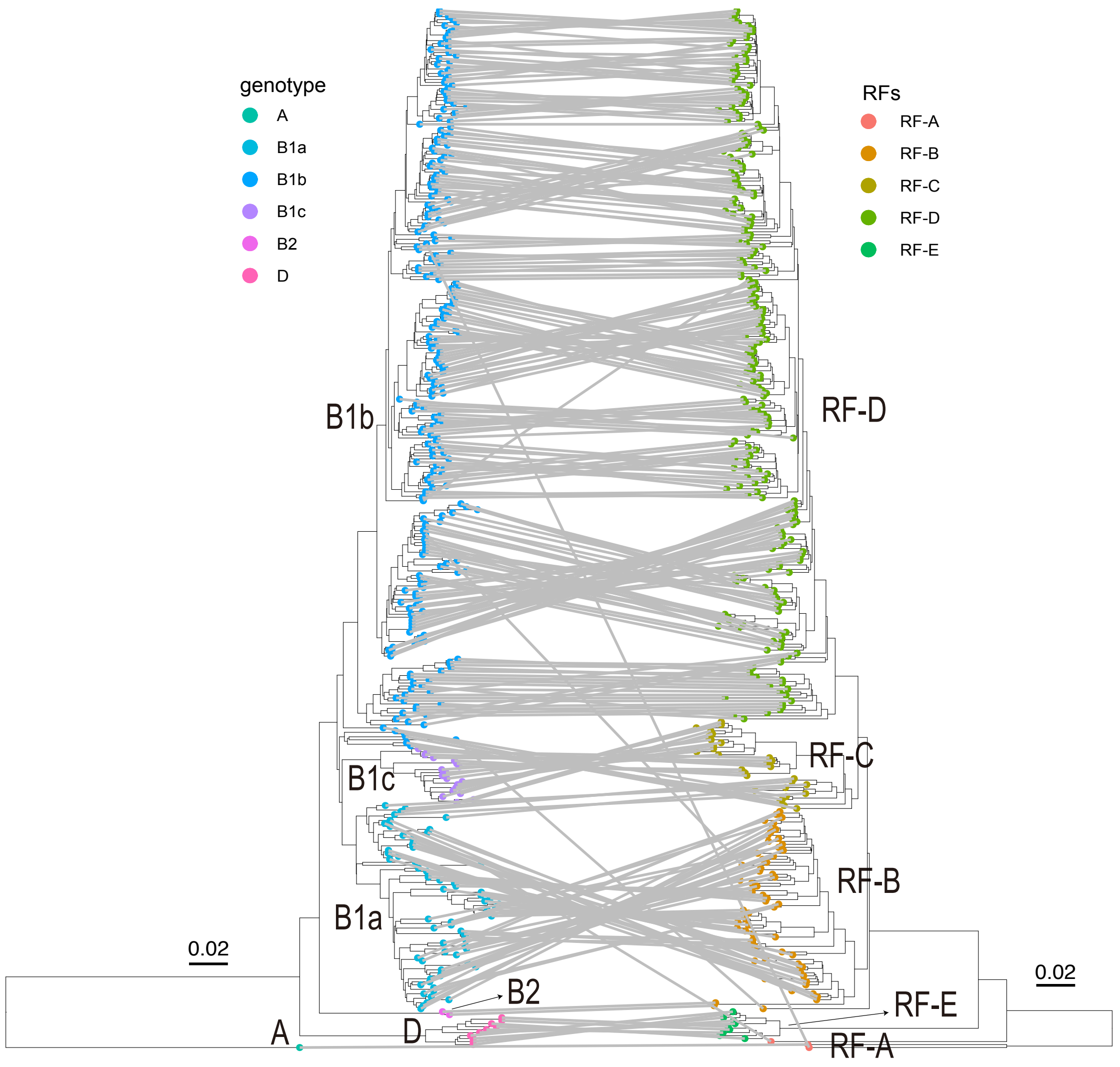

Table S1. Information on 345 coxsackievirus A16 (CVA16) sequences used for recombinant forms analysis in this study.

Strain name	GenBank accession No.	Isolation year	Countries	Origin
CVA16_B_CF310002_FRA_2012	LT617110.1	2012	France	GenBank
Y-22/CQ/CHN/2018	ON646272.1	2018	China	GenBank
HEV217/CHN/2016	MW713444.1	2016	China	GenBank
Tainan-5079-98	AF177911.1	1998	Taiwan	GenBank
shzh00-1	AY790926.1	2000	China	GenBank
shzh05-1	EU262658.1	2005	China	GenBank
FY18	EU812514.1	2008/5/1	China	GenBank
GZ08	FJ198212.1	2008/6/1	China	GenBank
SZ-HK08-3	GQ279368.1	2008/5/1	China	GenBank
SZ-HK08-7	GQ279371.1	2008/5/1	China	GenBank
XM-CA16-3560	HQ269389.1	2009/5/9	China	GenBank
KMM-08	HQ423141.1	2008/5/1	China	GenBank
THA-CA16-069	JF738004.1	2010/7/1	Thailand	GenBank
G20	JN590244.1	2010/5/24	China	GenBank
HN1662-HN-CHN-2010	JN674176.1	2010	China	GenBank
SH-CHN-2009	JQ034149.1	2010	China	GenBank
HQ09011181	JQ316639.1	2011/6/26	China	GenBank
Ningbo.CHN-028-2-2009	JQ354992.1	2009/4/2	China:	GenBank
CV-A16-genotypeA	JQ746659.1	NA	Malaysia	GenBank
PM-00033-07	JQ746660.1	2007	Malaysia	GenBank
PM-12284-99	JQ746661.1	1999	Malaysia	GenBank
PM-12727-99	JQ746662.1	1999	Malaysia	GenBank
PM-13884-97	JQ746663.1	1997	Malaysia	GenBank
PM-13998-00	JQ746664.1	2000	Malaysia	GenBank
PM-14660-97	JQ746665.1	1997	Malaysia	GenBank
PM-15765-00	JQ746666.1	2000	Malaysia	GenBank
PM-15922-00	JQ746667.1	2000	Malaysia	GenBank
PM-1651402-06	JQ746668.1	2006	Malaysia	GenBank
PM-16809-98	JQ746669.1	1998	Malaysia	GenBank
PM-16985-98	JQ746670.1	1998	Malaysia	GenBank
PM-1791021-07	JQ746671.1	2007	Malaysia	GenBank
PM-1795457-07	JQ746672.1	2007	Malaysia	GenBank
PM-22159-02	JQ746673.1	2002	Malaysia	GenBank
PM-22217-02	JQ746674.1	2002	Malaysia	GenBank
PM-23208-02	JQ746675.1	2002	Malaysia	GenBank
PM-31131-05	JQ746676.1	2005	Malaysia	GenBank
PM-31376-05	JQ746677.1	2005	Malaysia	GenBank
PM-35210-06	JQ746678.1	2006	Malaysia	GenBank

TS10-07	JX068827.1	2010/7/9	China	GenBank
BJ11-12	JX068828.1	2011/9/1	China	GenBank
TS10-08	JX068829.1	2010/8/1	China	GenBank
BJ11-03	JX068830.1	2011/3/1	China	GenBank
BJ-11-11	JX068831.1	2011/11/1	China	GenBank
BJ09-06	JX068832.1	2009/6/6	China	GenBank
BJ08-07	JX068833.1	2008/7/1	China	GenBank
BJCA08	JX481738.1	2008/2/11	China	GenBank
BJ1208	JX507808.1	NA	China	GenBank
Kor08-CVA16	JX839965.1	2008/6/1	South_Korea	GenBank
Wuhan0109-HuB-CHN-2011	JX986740.1	2011	China	GenBank
Wuhan0157-HuB-CHN-2011	JX986741.1	2011	China	GenBank
Wuhan0127-HuB-CHN-2011	JX986742.1	2011	China	GenBank
CA16-GD09-24	KC117317.1	2009	China:	GenBank
CA16-GD09-119	KC117318.1	2009	China	GenBank
G08	KC342228.1	NA	China	GenBank
YY157	KC507895.1	2010	China	GenBank
MAV	KC695830.1	NA	China	GenBank
XZ10-D-1	KC755228.1	2010	China	GenBank
ZJ10-73	KC755229.1	2010	China	GenBank
AH10-2	KC755230.1	2010	China	GenBank
AH10-12	KC755231.1	2010	China	GenBank
NJ10-31	KC755232.1	2010	China	GenBank
NJ10-75	KC755233.1	2010	China	GenBank
XZ10-C-1	KC755234.1	2010	China	GenBank
ZJ10-48	KC755235.1	2010	China	GenBank
CC024	KF055238.1	2010	China	GenBank
changchun028	KF055239.1	2010	China	GenBank
changchun029	KF055240.1	2010	China	GenBank
CC045	KF055241.1	2010	China	GenBank
changchun075	KF055242.1	2010	China	GenBank
CC090	KF055243.1	2010	China	GenBank
CC097	KF055244.1	2010	China	GenBank
CC163	KF055245.1	2010	China	GenBank
ZJ08-01	KF193620.1	2008	China	GenBank
SD09-05	KF193621.1	2009	China	GenBank
YN10-02	KF193622.1	2010	China	GenBank
HN11-03	KF193623.1	2011	China	GenBank
HN09-02	KF193624.1	2009	China	GenBank
GX10-01	KF193625.1	2010	China	GenBank
FJ10-03	KF193626.1	2010	China	GenBank
FJ09-02	KF193627.1	2009	China	GenBank
FJ09-01	KF193628.1	2009	China	GenBank
BJ10-03	KF193629.1	2010	China	GenBank

BJ10-01	KF193630.1	2010	China	GenBank
BJ10-02	KF193631.1	2010	China	GenBank
AH08-06	KF193632.1	2008	China	GenBank
CVA16-WIBP-P4-731	KF924762.1	2010/5/27	China	GenBank
DL16	KF991007.1	2012/5/18	China	GenBank
L23	KJ746492.1	2010	China	GenBank
CVA16-SZ29-CHN-2014	KM215267.1	2014/5/1	China	GenBank
Wh16	KM516102.1	2010/6/1	China	GenBank
2008-43-7	KP266573.1	2008/12/2	China	GenBank
CV-A16-P10-2013-China	KP289411.1	2013	China	GenBank
CV-A16-P1014-2013-China	KP289412.1	2013	China	GenBank
CV-A16-P187-2013-China	KP289413.1	2013	China	GenBank
CV-A16-P255-2013-China	KP289414.1	2013	China	GenBank
CV-A16-P301-2013-China	KP289415.1	2013	China	GenBank
CV-A16-P83-2013-China	KP289416.1	2013	China	GenBank
GDV126	KU163608.1	2010	China	GenBank
BJ14-3	KU254597.1	2014/7/9	China	GenBank
BJ14-4	KU254598.1	2014/7/9	China	GenBank
CA16-193	KU854873.1	2008	China	GenBank
CA16-194	KX056216.1	2008	China	GenBank
ensh01-CHN-12	KX058533.1	2012	China	GenBank
SiICRC04-TH-2011	KX372333.1	2011/8/10	Thailand	GenBank
SiICRC05-TH-2011	KX372334.1	2011/8/15	Thailand	GenBank
SiICRC06-TH-2011	KX372335.1	2011/8/16	Thailand	GenBank
SiICRC01-TH-2012	KX372336.1	2012/8/8	Thailand	GenBank
SiICRC02-TH-2012	KX372337.1	2012/12/1	Thailand	GenBank
SiICRC03-TH-2012	KX372338.1	2012/12/1	Thailand	GenBank
SiICRC01-TH-2014	KX372339.1	2014/7/8	Thailand	GenBank
CA16-196	KX580041.1	2008/8/7	China	GenBank
CVA16-Shenzhen36-CHN-2014	KX595291.1	2014/5/1	China	GenBank
CVA16-Shenzhen73-CHN-2014	KX595292.1	2014/6/1	China	GenBank
CVA16-Shenzhen74-CHN-2014	KX595293.1	2014/6/1	China	GenBank
CVA16-Shenzhen79-CHN-2014	KX595294.1	2014/7/1	China	GenBank
CVA16-Shenzhen179-CHN-2014	KX595295.1	2014/4/1	China	GenBank
393	KY014077.1	2008/8/28	China	GenBank
K168-8	KY088084.1	2010/8/6	China	GenBank
K11-YN-CHN-2011	KY425528.1	2011/6/6	China	GenBank
V1-YN-CHN-2015	KY425529.1	2015/6/13	China	GenBank
R5-YN-CHN-2011	KY425530.1	2011/6/2	China	GenBank
K34-YN-CHN-2011	KY425531.1	2011/5/1	China	GenBank
R68-YN-CHN-2009	KY425532.1	2009/5/24	China	GenBank
R35-YN-CHN-2012	KY425533.1	2012/6/23	China	GenBank
R34-YN-CHN-2012	KY425534.1	2012/6/23	China	GenBank
R37-YN-CHN-2013	KY425535.1	2013/10/14	China	GenBank

V37-YN-CHN-2014	KY425536.1	2014/6/24	China	GenBank
R141-YN-CHN-2009	KY425537.1	2009/6/24	China	GenBank
R254-YN-CHN-2010	KY425538.1	2010/5/18	China	GenBank
R255-YN-CHN-2010	KY425539.1	2010/5/18	China	GenBank
V86-YN-CHN-2015	KY425540.1	2015/6/19	China	GenBank
CV-A16-A01-BLR-IN	KY792576.1	2012/10/1	India	GenBank
CV-A16-A02-BLR-IN	KY792577.1	2012/10/1	India	GenBank
CV-A16-A06-BLR-IN	KY792578.1	2013/7/1	India	GenBank
CV-A16-A10-BLR-IN	KY792579.1	2013/7/1	India	GenBank
CV-A16-A13-BLR-IN	KY792580.1	2013/7/1	India	GenBank
CV-A16-A122-BLR-IN	KY792581.1	2015/6/1	India	GenBank
CV-A16-A128-BLR-IN	KY792582.1	2015/6/1	India	GenBank
CV-A16-M02-BLR-IN	KY792583.1	2013/4/1	India	GenBank
CV-A16-M69-BLR-IN	KY792584.1	2015/10/1	India	GenBank
CVA16_B_RP80_AUT_2003	LT617091.1	2003	Austria	GenBank
CVA16_B_BER53-2_DEU_2003	LT617092.1	2003	Germany	GenBank
CVA16_B_BUD22_HUN_2008	LT617093.1	2008	Hungary	GenBank
CVA16_B_BUD25_HUN_2008	LT617094.1	2008	Hungary	GenBank
CVA16_B_STU7_DEU_2009	LT617096.1	2009	Germany	GenBank
CVA16_B_STU4_DEU_2010	LT617097.1	2010	Germany	GenBank
CVA16_B_CF145057_FRA_2010	LT617098.1	2010	France	GenBank
CVA16_B_CF160074_FRA_2010	LT617099.1	2010	France	GenBank
CVA16_B_CF166109_FRA_2010	LT617100.1	2010	France	GenBank
CVA16_B_CF187036_FRA_2010	LT617101.1	2010	France	GenBank
CVA16_B_CF279014_FRA_2010	LT617102.1	2010	France	GenBank
CVA16_B_CF312044_FRA_2010	LT617103.1	2010	France	GenBank
CVA16_B_CF223065_FRA_2011	LT617104.1	2011	France	GenBank
CVA16_C_CF350028_FRA_2011	LT617105.1	2011	France	GenBank
CVA16_C_CF172083_FRA_2012	LT617106.1	2012	France	GenBank
CVA16_C_CF178025_FRA_2012	LT617107.1	2012	France	GenBank
CVA16_C_CF178036_FRA_2012	LT617108.1	2012	France	GenBank
CVA16_C_CF193053_FRA_2012	LT617109.1	2012	France	GenBank
CVA16_C_PAR155055_FRA_2014	LT617111.1	2014	France	GenBank
CVA16_C_MET171023_FRA_2014	LT617112.1	2014	France	GenBank
CVA16_C_LYO171046_FRA_2014	LT617113.1	2014	France	GenBank
CVA16_C_PAR181046_FRA_2014	LT617114.1	2014	France	GenBank
CVA16_C_PAR190033_FRA_2014	LT617115.1	2014	France	GenBank
USA-CT-2016-19518	MF189180.1	2016/11/16	USA	GenBank
ZJ6	MF434051.1	2008/8/28	China	GenBank
HF146-SD-CHN-2008	MG450666.1	2008	China	GenBank
TA271-Shandong-China-2015	MG674827.1	2015/7/18	China	GenBank
CVA16-Shenzhen500-CHN-2014	MH010198.1	2014/12/1	China	GenBank
CVA16-Shenzhen469-CHN-2015	MH010199.1	2015/10/1	China	GenBank
CVA16-Shenzhen87-CHN-2016	MH010200.1	2016/4/1	China	GenBank

CVA16-Shenzhen289-CHN-2016	MH010201.1	2016/6/1	China	GenBank
CVA16-Shenzhen76-CHN-2017	MH010202.1	2017/3/1	China	GenBank
CVA16-Shenzhen169-CHN-2017	MH010203.1	2017/5/1	China	GenBank
CVA16-Shenzhen174-CHN-2017	MH010204.1	2017/5/1	China	GenBank
CVA16-Shenzhen189-CHN-2017	MH010205.1	2017/5/1	China	GenBank
CVA16-Shenzhen220-CHN-2017	MH010206.1	2017/5/1	China	GenBank
C028-CHW-AUS-2016	MH111067.1	2016/4/22	Australia	GenBank
C105-CHW-AUS-2016	MH111068.1	2017/1/18	Australia	GenBank
C123-CHW-AUS-2016	MH111070.1	2017/2/17	Australia	GenBank
C138-CHW-AUS-2016	MH111071.1	2016/12/14	Australia	GenBank
C151-CHW-AUS-2016	MH111072.1	2017/4/16	Australia	GenBank
G-10	U05876.1	1951	South_Africa	GenBank
AH17-18/AH/East/CHN/2017-02-12	MT211988	2017/2/12	China	GenBank
AH18-25/AH/East/CHN/2018-04-26	MT211989	2018/4/26	China	GenBank
BJ16-68/BJ/Central/CHN/2016-08-01	MT211990	2016/8/1	China	GenBank
BJ17-14/BJ/Central/CHN/2017-02-15	MT211991	2017/2/15	China	GenBank
BJ18-58/BJ/Central/CHN/2018-08-20	MT211992	2018/8/20	China	GenBank
CQ16-104/CQ/West/CHN/2016-01-13	MT211993	2016/1/13	China	GenBank
CQ17-38/CQ/West/CHN/2017-04-12	MT211994	2017/4/12	China	GenBank
CQ18-3/CQ/West/CHN/2018-06-28	MT211995	2018/6/28	China	GenBank
GD16-105/GD/South/CHN/2016-06-17	MT211996	2016/6/17	China	GenBank
GD17-81/GD/South/CHN/2017-06-01	MT211997	2017/6/1	China	GenBank
GD18-104/GD/South/CHN/2018-08-14	MT211998	2018/8/14	China	GenBank
GS16-110/GS/West/CHN/2016-05-30	MT211999	2016/5/30	China	GenBank
GS17-636/GS/West/CHN/2017-06-15	MT212000	2017/6/15	China	GenBank
GS17-658/GS/West/CHN/2017-06-15	MT212001	2017/6/15	China	GenBank
GS18-179/GS/West/CHN/2018-05-14	MT212002	2018/5/14	China	GenBank
GZ16-QN035/GZ/South/CHN/2016-06-30	MT212003	2016/6/30	China	GenBank
HAN17-66/HaN/South/CHN/2017-	MT212004	2017/12/11	China	GenBank

12-11				
HAN18-8/HaN/South/CHN/2018-02-28	MT212005	2018/2/28	China	GenBank
HB16-54015/HB/Central/CHN/2016-01-15	MT212006	2016/1/15	China	GenBank
HB17-54371/HB/Central/CHN/2017-07-07	MT212007	2017/7/7	China	GenBank
HB18-20/HB/Central/CHN/2018-05-16	MT212008	2018/5/16	China	GenBank
HEN17-108/HeN/Central/CHN/2017-06-30	MT212009	2017/6/30	China	GenBank
HeN18-400/HeN/Central/CHN/2018-07-11	MT212010	2018/7/11	China	GenBank
HLJ16-HH2016033/HLJ/North/CHN/2016-09-19	MT212011	2016/9/19	China	GenBank
HLJ18-16/HLJ/North/CHN/2018-08-05	MT212012	2018/8/5	China	GenBank
HuN16-75/HuN/South/CHN/2016-05-09	MT212013	2016/5/9	China	GenBank
HUN17-33/HuN/South/CHN/2017-08-07	MT212014	2017/8/7	China	GenBank
HuN18-5/HuN/South/CHN/2018-02-26	MT212015	2018/2/26	China	GenBank
JL18-97/JL/North/CHN/2018-08-05	MT212016	2018/8/5	China	GenBank
JX16-126/JX/East/CHN/2016-12-27	MT212017	2016/12/27	China	GenBank
JX18-30/JX/East/CHN/2018-03-26	MT212018	2018/3/26	China	GenBank
LN16-23-12/LN/North/CHN/2016-07-11	MT212019	2016/7/11	China	GenBank
QH16-8/QH/West/CHN/2016-07-07	MT212020	2016/7/7	China	GenBank
QH17-104/QH/West/CHN/2017-10-13	MT212021	2017/10/13	China	GenBank
QH18-5/QH/West/CHN/2018-05-12	MT212022	2018/5/12	China	GenBank
SAX17-50/SaX/Central/CHN/2017-09-16	MT212023	2017/9/16	China	GenBank
SD16-101/SD/East/CHN/2016-07-22	MT212024	2016/7/22	China	GenBank
SX17-286/SX/Central/CHN/2017-06-16	MT212025	2017/6/16	China	GenBank
TJ16-7/TJ/Central/CHN/2016-02-29	MT212026	2016/2/29	China	GenBank
TJ17-36/TJ/Central/CHN/2017-03-13	MT212027	2017/3/13	China	GenBank

TJ18-63/TJ/Central/CHN/2018-05-28	MT212028	2018/5/28	China	GenBank
XJ17-212/XJ/West/CHN/2017-10-04	MT212029	2017/10/4	China	GenBank
XJ18-019/XJ/West/CHN/2018-04-09	MT212030	2018/4/9	China	GenBank
YN16-253/YN/South/CHN/2016-05-17	MT212031	2016/5/17	China	GenBank
YN17-J29/YN/South/CHN/2017-02-07	MT212032	2017/2/7	China	GenBank
YN18-A67/YN/South/CHN/2018-03-14	MT212033	2018/3/14	China	GenBank
ZJ16-14/ZJ/East/CHN/2016-03-30	MT212034	2016/3/30	China	GenBank
ZJ17-91/ZJ/East/CHN/2017-07-02	MT212035	2017/7/2	China	GenBank
ZJ18-24/ZJ/East/CHN/2018-01-04	MT212036	2018/1/4	China	GenBank

Table S2. Model selection using BEAST based on the path sampling (PS) and stepping stone sampling (SS) values.

Data sets name	Molecular clock model	Coalescent tree prior	ESS	PS	SS
Strict-GMRF	Strict clock	GMRF	>200	-24564.53	-24582.06
Strict-CONSTANT	Strict clock	constant size	>200	-24462.68	-24475.84
Strict-EXPONENTIAL	Strict clock	exponential growth	>200	-24455.31	-24469.39
Strict-SKYLINE	Strict clock	BSP	<200	-24410.41	-24430.42
Strict-EBSP	Strict clock	EBSP	N/A	N/A	N/A
Strict-SKYGRID	Strict clock	SkyGrid	>200	-24433.25	-24456.45
E-GMRF	Uncorrelated exponential relaxed clock	GMRF	<200	-24378.51	-24397.76
E-CONSTANT	Uncorrelated exponential relaxed clock	constant size	<200	-24397.2	-24415.79
E-EXPONENTIAL	Uncorrelated exponential relaxed clock	exponential growth	<200	-24360.12	-24377.6
E-SKYLINE	Uncorrelated exponential relaxed clock	BSP	<200	-24301.02	-24327.24
E-EBSP	Uncorrelated exponential relaxed clock	EBSP	N/A	N/A	N/A
E-SKYGRID	Uncorrelated exponential relaxed clock	SkyGrid	<200	-24335.22	-24356.47

L-GMRF	Uncorrelated lognormal relaxed clock	GMRF	>200	-24368.62	-24397.29
L-CONSTANT	Uncorrelated lognormal relaxed clock	constant size	<200	-24405.68	-24421.54
L-EXPONENTIAL	Uncorrelated lognormal relaxed clock	exponential growth	<200	-24356	-24375.24
L-SKYLINE	Uncorrelated lognormal relaxed clock	BSP	<200	-24302.83	-24328.22
L-EBSP	Uncorrelated lognormal relaxed clock	EBSP	N/A	N/A	N/A
L-SKYGRID	Uncorrelated lognormal relaxed clock	SkyGrid	<200	-24338.14	-24357.96

Table S3. Information on 1663-sequence dataset used in this study.

GenBank	isolate	country	Collection date	Isolation source
AB465366.1	24/Toyama/1981	Japan	1981	throat swab
AB465367.1	379/Toyama/1984	Japan	1984	vesicle swab
AB465368.1	576/Toyama/1988	Japan	1988	feces
AB465369.1	107/Toyama/1990	Japan	1990	vesicle swab
AB465370.1	392/Toyama/1995	Japan	1995	throat swab
AB465371.1	927/Toyama/1998	Japan	1998	feces
AB465372.1	188/Toyama/2000	Japan	2000	throat swab
AB465373.1	223/Toyama/2000	Japan	2000	vesicle swab
AB465374.1	124/Toyama/2002	Japan	2002	feces
AB465375.1	227/Toyama/2002	Japan	2002	feces
AB465376.1	228/Toyama/2002	Japan	2002	throat swab
AB465377.1	246/Toyama/2002	Japan	2002	feces
AB465378.1	247/Toyama/2002	Japan	2002	feces
AB465379.1	248/Toyama/2002	Japan	2002	feces
AB465380.1	249/Toyama/2002	Japan	2002	feces
AB465385.1	255/Toyama/2002	Japan	2002	feces
AB465386.1	256/Toyama/2002	Japan	2002	feces
AB465387.1	258/Toyama/2002	Japan	2002	feces
AB465388.1	259/Toyama/2002	Japan	2002	feces
AB465389.1	260/Toyama/2002	Japan	2002	feces
AB465392.1	263/Toyama/2002	Japan	2002	feces
AB465394.1	265/Toyama/2002	Japan	2002	feces
AB465396.1	283/Toyama/2002	Japan	2002	throat swab
AB465397.1	295/Toyama/2002	Japan	2002	feces

AB465398.1	298/Toyama/2002	Japan	2002	throat swab
AB465399.1	120/Toyama/2003	Japan	2003	throat swab
AB465400.1	290/Toyama/2003	Japan	2003	throat swab
AB465401.1	355/Toyama/2005	Japan	2005	throat swab
AB465402.1	418/Toyama/2006	Japan	2006	throat swab
AB465403.1	419/Toyama/2006	Japan	2006	feces
AB465404.1	459/Toyama/2007	Japan	2007	feces
AB465405.1	460/Toyama/2007	Japan	2007	throat swab
AB634286.1	Y88-5375	Japan	1998	throat swab
AB634287.1	Y92-2389	Japan	1992	throat swab
AB634288.1	Y92-2773	Japan	1992	throat swab
AB634289.1	Y92-2710	Japan	1992	throat swab
AB634290.1	Y92-2855	Japan	1992	throat swab
AB634291.1	Y92-2861	Japan	1992	throat swab
AB634292.1	Y92-2882	Japan	1992	throat swab
AB634293.1	Y92-2998	Japan	1992	throat swab
AB634294.1	Y93-2054	Japan	1993	throat swab
AB634295.1	Y95-2096	Japan	1995	throat swab
AB634296.1	Y95-2447	Japan	1995	throat swab
AB634297.1	Y95-2135	Japan	1995	throat swab
AB634298.1	Y95-2142	Japan	1995	throat swab
AB634299.1	Y95-2164	Japan	1995	throat swab
AB634300.1	Y95-2200	Japan	1995	throat swab
AB634301.1	Y95-2259	Japan	1995	throat swab
AB634302.1	Y95-2260	Japan	1995	throat swab
AB634303.1	Y95-2313	Japan	1995	throat swab
AB634304.1	Y95-2318	Japan	1995	throat swab
AB634305.1	Y95-2345	Japan	1995	throat swab
AB634306.1	Y95-2386	Japan	1995	throat swab
AB634307.1	Y95-2390	Japan	1995	throat swab
AB634308.1	Y95-2403	Japan	1995	throat swab
AB634309.1	Y95-2854	Japan	1995	throat swab
AB634310.1	Y95-2874	Japan	1995	throat swab
AB634311.1	Y95-2932	Japan	1995	throat swab
AB634312.1	Y97-861	Japan	1997	throat swab
AB634313.1	Y97-1087	Japan	1997	throat swab
AB634314.1	Y97-1135	Japan	1997	throat swab
AB634315.1	Y97-1174	Japan	1997	throat swab
AB634316.1	Y97-1448	Japan	1997	throat swab
AB634317.1	451-Yamagata-1997	Japan	1997	throat swab
AB634318.1	494-Yamagata-1997	Japan	1997	throat swab
AB634319.1	496-Yamagata-1997	Japan	1997	throat swab
AB634320.1	Y98-891	Japan	1998	throat swab

AB634321.1	Y98-1159	Japan	1998	throat swab
AB634322.1	721-Yamagata-1998	Japan	1998	throat swab
AB634323.1	737-Yamagata-1998	Japan	1998	throat swab
AB634324.1	808-Yamagata-1998	Japan	1998	throat swab
AB634325.1	624-Yamagata-2000	Japan	2000	throat swab
AB634326.1	648-Yamagata-2000	Japan	2000	throat swab
AB634338.1	787-Yamagata-2000	Japan	2000	throat swab
AB634339.1	820-Yamagata-2000	Japan	2000	throat swab
AB634340.1	835-Yamagata-2000	Japan	2000	throat swab
AB634341.1	836-Yamagata-2000	Japan	2000	throat swab
AB634342.1	904-Yamagata-2000	Japan	2000	throat swab
AB634343.1	905-Yamagata-2000	Japan	2000	throat swab
AB634349.1	933-Yamagata-2000	Japan	2000	throat swab
AB634350.1	939-Yamagata-2000	Japan	2000	throat swab
AB634351.1	1007-Yamagata-2000	Japan	2000	throat swab
AB634352.1	995-Yamagata-2000	Japan	2000	throat swab
AB634353.1	1026-Yamagata-2000	Japan	2000	throat swab
AB634354.1	1027-Yamagata-2000	Japan	2000	throat swab
AB634355.1	1049-Yamagata-2000	Japan	2000	throat swab
AB634356.1	1071-Yamagata-2000	Japan	2000	throat swab
AB634357.1	1060-Yamagata-2000	Japan	2000	throat swab
AB634358.1	1085-Yamagata-2000	Japan	2000	throat swab
AB634359.1	1090-Yamagata-2000	Japan	2000	throat swab
AB634360.1	1111-Yamagata-2000	Japan	2000	throat swab
AB634361.1	1129-Yamagata-2000	Japan	2000	throat swab
AB634362.1	1203-Yamagata-2000	Japan	2000	throat swab
AB634363.1	1113-Yamagata-2001	Japan	2001	throat swab
AB634364.1	1120-Yamagata-2001	Japan	2001	throat swab
AB634369.1	1250-Yamagata-2001	Japan	2001	throat swab
AB634370.1	1393-Yamagata-2001	Japan	2001	throat swab
AB634371.1	1562-Yamagata-2001	Japan	2001	throat swab
AB634372.1	1581-Yamagata-2001	Japan	2001	throat swab
AB634373.1	1649-Yamagata-2001	Japan	2001	throat swab
AB634374.1	2011-Yamagata-2001	Japan	2001	throat swab
AB634375.1	995-Yamagata-2002	Japan	2002	throat swab
AB634376.1	1687-Yamagata-2002	Japan	2002	throat swab
AB634377.1	1872-Yamagata-2002	Japan	2002	throat swab
AB634380.1	1747-Yamagata-2002	Japan	2002	throat swab
AB634381.1	2527-Yamagata-2002	Japan	2002	throat swab
AB634382.1	2067-Yamagata-2002	Japan	2002	throat swab
AB634383.1	2183-Yamagata-2002	Japan	2002	throat swab
AB634384.1	2319-Yamagata-2002	Japan	2002	throat swab
AB634385.1	2679-Yamagata-2002	Japan	2002	throat swab

AB634386.1	2724-Yamagata-2002	Japan	2002	throat swab
AB634387.1	3184-Yamagata-2002	Japan	2002	throat swab
AB634388.1	2633-Yamagata-2003	Japan	2003	throat swab
AB634389.1	2870-Yamagata-2003	Japan	2003	throat swab
AB634390.1	2929-Yamagata-2003	Japan	2003	throat swab
AB634391.1	2065-Yamagata-2004	Japan	2004	throat swab
AB634392.1	2000-Yamagata-2005	Japan	2005	throat swab
AB634393.1	2188-Yamagata-2005	Japan	2005	throat swab
AB634394.1	2222-Yamagata-2005	Japan	2005	throat swab
AB634395.1	2437-Yamagata-2005	Japan	2005	throat swab
AB634396.1	2443-Yamagata-2005	Japan	2005	throat swab
AB634397.1	2441-Yamagata-2005	Japan	2005	throat swab
AB634398.1	2295-Yamagata-2006	Japan	2006	throat swab
AB634399.1	2511-Yamagata-2006	Japan	2006	throat swab
AB634400.1	2402-Yamagata-2006	Japan	2006	throat swab
AB634401.1	2421-Yamagata-2006	Japan	2006	throat swab
AB634402.1	2620-Yamagata-2006	Japan	2006	throat swab
AB634403.1	2681-Yamagata-2006	Japan	2006	throat swab
AB634404.1	584-Yamagata-2007	Japan	2007	throat swab
AB634405.1	2686-Yamagata-2006	Japan	2006	throat swab
AB634406.1	3162-Yamagata-2006	Japan	2006	throat swab
AB634407.1	3195-Yamagata-2006	Japan	2006	throat swab
AB634408.1	3242-Yamagata-2006	Japan	2006	throat swab
AB634409.1	512-Yamagata-2007	Japan	2007	throat swab
AB634410.1	1122-Yamagata-2007	Japan	2007	throat swab
AB634412.1	1098-Yamagata-2007	Japan	2007	throat swab
AB634413.1	1618-Yamagata-2007	Japan	2007	throat swab
AB634414.1	1246-Yamagata-2007	Japan	2007	throat swab
AB634415.1	1249-Yamagata-2007	Japan	2007	throat swab
AB634417.1	1293-Yamagata-2007	Japan	2007	throat swab
AB634418.1	1430-Yamagata-2007	Japan	2007	throat swab
AB634419.1	1600-Yamagata-2007	Japan	2007	throat swab
AB634420.1	1872-Yamagata-2007	Japan	2007	throat swab
AB634421.1	1172-Yamagata-2008	Japan	2008	throat swab
AB634422.1	1316-Yamagata-2008	Japan	2008	throat swab
AB634423.1	1386-Yamagata-2008	Japan	2008	throat swab
AB634424.1	1613-Yamagata-2008	Japan	2008	throat swab
AB634425.1	1694-Yamagata-2008	Japan	2008	throat swab
AB634426.1	1768-Yamagata-2008	Japan	2008	throat swab
AB634427.1	1817-Yamagata-2008	Japan	2008	throat swab
AB634428.1	1892-Yamagata-2008	Japan	2008	throat swab
AB634429.1	2077-Yamagata-2009	Japan	2009	throat swab
AB634430.1	3350-Yamagata-2009	Japan	2009	throat swab

AB634431.1	3351-Yamagata-2009	Japan	2009	throat swab
AB634437.1	173-Yamagata-2010	Japan	2010	throat swab
AB634438.1	1142-Yamagata-2010	Japan	2010	throat swab
AB634440.1	577-Yamagata-2010	Japan	2010	throat swab
AB634441.1	820-Yamagata-2010	Japan	2010	throat swab
AB634442.1	1160-Yamagata-2010	Japan	2010	throat swab
AB634443.1	1289-Yamagata-2010	Japan	2010	throat swab
AB634446.1	1327-Yamagata-2010	Japan	2010	throat swab
AB634447.1	1375-Yamagata-2010	Japan	2010	throat swab
AB634448.1	1575-Yamagata-2010	Japan	2010	throat swab
AB634449.1	1700-Yamagata-2010	Japan	2010	throat swab
AB634450.1	1876-Yamagata-2010	Japan	2010	throat swab
AB634451.1	1973-Yamagata-2010	Japan	2010	throat swab
AB634452.1	1931-Yamagata-2010	Japan	2010	throat swab
FR798001.1	ESP08/54682	Spain	2008	feces
HE572993.1	CF160074_FRA10	France	2010	throat swab
HE572994.1	CF279014_FRA10	France	2010	throat swab
HE572995.1	CF281008_FRA10	France	2010	throat swab
HE572996.1	CF301047_FRA10	France	2010	throat swab
HE572997.1	CF312044_FRA10	France	2010	throat swab
HE572998.1	CF323006_FRA10	France	2010	throat swab
HE573000.1	CF335030_FRA10	France	2010	throat swab
HE573002.1	CF341014_FRA10	France	2010	throat swab
HE573003.1	CF341020_FRA10	France	2010	throat swab
HE573004.1	CF348018_FRA10	France	2010	throat swab
HE573005.1	CF355014_FRA10	France	2010	throat swab
HE573006.1	CF355015_FRA10	France	2010	throat swab
HE573007.1	CF361090_FRA10	France	2010	throat swab
JF695002.1	CA16/HN1120/CHN/2010	China	2010	feces
JF695003.1	CA16/HN1129/CHN/2010	China	2010	feces
JF695004.1	CA16/HN1131/CHN/2010	China	2010	feces
JF695005.1	CA16/HN1514/CHN/2010	China	2010	feces
JF695006.1	CA16/HN1516/CHN/2010	China	2010	feces
JF695007.1	CA16/HN1539/CHN/2010	China	2010	feces
JF695008.1	CA16/HN1661/CHN/2010	China	2010	feces
JF695009.1	CA16/HN1662/CHN/2010	China	2010	feces
JF695010.1	CA16/HN1668/CHN/2010	China	2010	feces
JF695011.1	CA16/HN1726/CHN/2010	China	2010	feces
JQ315095.1	2009szk028	China	2009	feces
JQ315096.1	2009szk039	China	2009	feces
JQ315097.1	2010szk102	China	2010	feces
JQ315098.1	2010szk113	China	2010	feces
JQ315099.1	2010szk017	China	2010	feces

JQ315100.1	2008szk290	China	2008	feces
JQ315101.1	YYS10-044	China	2010	throat swab
JQ315102.1	YYS10-062	China	2010	throat swab
JQ315103.1	YYS10-067	China	2010	feces
JQ315104.1	2010Changdao-166	China	2010	feces
JQ315105.1	2010Changdao-185	China	2010	feces
JQ315106.1	2010Changdao-191	China	2010	feces
JQ315107.1	2010Changdao-201	China	2010	feces
JQ315108.1	2010Changdao-234	China	2010	feces
JQ315109.1	2010Changdao-412	China	2010	feces
JQ315110.1	2010Changdao-439	China	2010	feces
JQ315111.1	2010Changdao-450	China	2010	feces
JQ315112.1	2010Changdao-470	China	2010	feces
JQ315113.1	2010Changdao-475	China	2010	feces
JQ315114.1	2010Changdao-477	China	2010	feces
JQ315115.1	2010Changdao-488	China	2010	feces
JQ316639.1	HQ09011181	China	2011	feces
JQ409496.1	MAS04/AH/CHN/2010	China	2010	throat swab
JQ409497.1	MAS03/AH/CHN/2010	China	2010	throat swab
JQ409498.1	MAS02/AH/CHN/2010	China	2010	anal swab
JQ409499.1	MAS01/AH/CHN/2010	China	2010	anal swab
JX455098.1	TJ2010-CA16-08	China	2010	feces
JX455099.1	TJ2010-CA16-21	China	2010	feces
JX455100.1	TJ2010-CA16-22	China	2010	feces
JX455101.1	TJ2010-CA16-38	China	2010	feces
JX473412.1	CVA16-SHZH2011-0501	China	2011	feces
JX473414.1	CVA16-SHZH2011-0503	China	2011	feces
JX473415.1	CVA16-SHZH2011-0504	China	2011	feces
JX473416.1	CVA16-SHZH2011-0505	China	2011	feces
JX473417.1	CVA16-SHZH2011-0506	China	2011	feces
JX473418.1	CVA16-SHZH2011-0601	China	2011	feces
JX473419.1	CVA16-SHZH2011-0701	China	2011	feces
JX473425.1	CVA16-SHZH2011-0901	China	2011	feces
JX473426.1	CVA16-SHZH2011-0902	China	2011	feces
JX473427.1	CVA16-SHZH2011-0903	China	2011	feces
JX473430.1	CVA16-SHZH2011-1001	China	2011	feces
JX473432.1	CVA16-SHZH2011-1003	China	2011	feces
JX473435.1	CVA16-SHZH2011-1101	China	2011	feces
JX473436.1	CVA16-SHZH2011-1102	China	2011	feces
JX473438.1	CVA16-SHZH2011-1104	China	2011	feces
JX481738.1	BJCA08	China	2008	throat swab
JX839965.1	Kor08-CVA16	South Korea	2008	feces

JX975764.1	Wuhan0321/HuB/CHN/2012	China	2012	throat swab
JX975765.1	Wuhan0351/HuB/CHN/2012	China	2012	throat swab
JX975766.1	Wuhan0334/HuB/CHN/2012	China	2012	throat swab
JX975767.1	Ezhou0114/HuB/CHN/2011	China	2011	throat swab
JX975768.1	Wuhan0171/HuB/CHN/2011	China	2011	throat swab
JX975769.1	Wuhan0263/HuB/CHN/2011	China	2011	throat swab
JX975770.1	Wuhan0161/HuB/CHN/2011	China	2011	throat swab
JX975771.1	Wuhan0184/HuB/CHN/2011	China	2011	throat swab
JX975772.1	Wuhan0267/HuB/CHN/2011	China	2011	throat swab
JX975773.1	Wuhan0239/HuB/CHN/2011	China	2011	throat swab
JX975774.1	Wuhan0255/HuB/CHN/2011	China	2011	throat swab
JX975775.1	Wuhan0156/HuB/CHN/2011	China	2011	throat swab
JX975776.1	Wuhan0169/HuB/CHN/2011	China	2011	throat swab
JX975777.1	Wuhan0236/HuB/CHN/2011	China	2011	throat swab
JX975778.1	Wuhan0272/HuB/CHN/2011	China	2011	throat swab
JX975779.1	Wuhan0147/HuB/CHN/2011	China	2011	throat swab
JX975780.1	Wuhan0152/HuB/CHN/2011	China	2011	throat swab
JX975781.1	Wuhan0112/HuB/CHN/2011	China	2011	throat swab
JX975782.1	Wuhan0226/HuB/CHN/2011	China	2011	throat swab
JX975783.1	Wuhan0138/HuB/CHN/2011	China	2011	throat swab
JX975784.1	Wuhan0100/HuB/CHN/2011	China	2011	throat swab
JX975785.1	Wuhan0294/HuB/CHN/2011	China	2011	throat swab
JX975786.1	Wuhan0143/HuB/CHN/2011	China	2011	throat swab
JX975787.1	Wuhan0237/HuB/CHN/2011	China	2011	throat swab
JX975788.1	Wuhan0176/HuB/CHN/2011	China	2011	throat swab
JX975789.1	Wuhan0291/HuB/CHN/2011	China	2011	throat swab
JX975790.1	Wuhan0257/HuB/CHN/2011	China	2011	throat swab
JX975791.1	Wuhan0286/HuB/CHN/2011	China	2011	throat swab
JX975792.1	Wuhan0297/HuB/CHN/2011	China	2011	throat swab
JX975793.1	Wuhan0127/HuB/CHN/2011	China	2011	throat swab
JX975794.1	Wuhan0203/HuB/CHN/2011	China	2011	throat swab
JX975795.1	Wuhan0149/HuB/CHN/2011	China	2011	throat swab
JX975796.1	Wuhan0210/HuB/CHN/2011	China	2011	throat swab
JX975797.1	Wuhan0232/HuB/CHN/2011	China	2011	throat swab
JX975798.1	Wuhan0289/HuB/CHN/2011	China	2011	throat swab
JX975799.1	Wuhan0158/HuB/CHN/2011	China	2011	throat swab
JX975800.1	Wuhan0136/HuB/CHN/2011	China	2011	throat swab
JX975801.1	Wuhan0109/HuB/CHN/2011	China	2011	throat swab
JX975802.1	Macheng0117/HuB/CHN/2011	China	2011	throat swab
JX975803.1	Xiaogan0172/HuB/CHN/2011	China	2011	throat swab
JX975804.1	Wuhan0109/HuB/CHN/20111	China	2011	throat swab
JX986740.1	Wuhan0109/HuB/CHN/2011	China	2011	throat swab
JX986741.1	Wuhan0157/HuB/CHN/2011	China	2011	throat swab

JX986742.1	Wuhan0127/HuB/CHN/2011	China	2011	throat swab
KC117317.1	CA16/GD09/24	China	2009	throat swab
KC117318.1	CA16/GD09/119	China	2009	throat swab
KC787157.2	09C38CMR	Cameroon	2009	feces
KC787164.2	11C42CMR	Cameroon	2009	feces
KC787165.2	11C51CMR	Cameroon	2009	feces
KC866816.1	JB14080005	China	2008	feces
KC866818.1	JB14080026	China	2008	feces
KC866819.1	JB14080086	China	2008	feces
KC866820.1	JB14080087	China	2008	feces
KC866821.1	JB14080092	China	2008	feces
KC866822.1	JB14080101	China	2008	feces
KC866823.1	JB14080102	China	2008	feces
KC866824.1	JB14080118	China	2008	feces
KC866825.1	JB14080119	China	2008	feces
KC866826.1	JB14080122	China	2008	feces
KC866827.1	JB14080126	China	2008	feces
KC866828.1	JB14080128	China	2008	feces
KC866830.1	JB14080241	China	2008	feces
KC866831.1	JB14080257	China	2008	feces
KC866832.1	JB14080265	China	2008	feces
KC866833.1	JB14080408	China	2008	feces
KC866835.1	JB14080447	China	2008	feces
KC866836.1	JB14080477	China	2008	feces
KC866837.1	JB141090082	China	2009	feces
KC866838.1	JB141090092	China	2009	feces
KC866839.1	JB141090101	China	2009	feces
KC866840.1	JB143090004	China	2009	feces
KC866841.1	JB143090010	China	2009	feces
KC866843.1	JB143090017	China	2009	feces
KC866844.1	JB143090022	China	2009	feces
KC866845.1	JB143090023	China	2009	feces
KC866846.1	JB143090024	China	2009	feces
KC866847.1	JB143090037	China	2009	feces
KC866848.1	JB143090051	China	2009	feces
KC866849.1	JB143090053	China	2009	feces
KC866850.1	JB143090055	China	2009	feces
KC866851.1	JB143090056	China	2009	feces
KC866852.1	JB143090061	China	2009	feces
KC866853.1	JB143090067	China	2009	feces
KC866854.1	JB143090073	China	2009	feces
KC866855.1	JB143090082	China	2009	feces
KC866856.1	JB141030026	China	2010	feces

KC866857.1	JB141030028	China	2010	feces
KC866858.1	JB141030100	China	2010	feces
KC866859.1	JB141030106	China	2010	feces
KC866860.1	JB141030203	China	2010	feces
KC866861.1	JB141030204	China	2010	feces
KC866862.1	JB141030229	China	2010	feces
KC866863.1	JB141030230	China	2010	feces
KC866864.1	JB141030268	China	2010	feces
KC866865.1	JB141230001	China	2012	feces
KC866866.1	JB141230018	China	2012	feces
KC866867.1	JB141230038	China	2012	feces
KC866868.1	JB141230041	China	2012	feces
KC866869.1	JB141230043	China	2012	feces
KC866875.1	JB141230056	China	2012	feces
KC866876.1	JB141230057	China	2012	feces
KC866877.1	JB141230058	China	2012	feces
KC866878.1	JB141230100	China	2012	feces
KC866879.1	JB141230101	China	2012	feces
KC866881.1	JB141230107	China	2012	feces
KC866884.1	JB141230135	China	2012	feces
KC866885.1	JB141230144	China	2012	feces
KC866886.1	JB141230148	China	2012	feces
KC866887.1	JB141230160	China	2012	feces
KC866888.1	JB141230185	China	2012	feces
KC866889.1	JB141230194	China	2012	feces
KC866891.1	JB141210001	China	2012	feces
KC866893.1	JB141210013	China	2012	feces
KC866894.1	JB141230210	China	2012	feces
KC866895.1	JB141230213	China	2012	feces
KC866896.1	JB141230214	China	2012	feces
KC866897.1	JB141230241	China	2012	feces
KC866898.1	JB141230249	China	2012	feces
KC866899.1	JB141230365	China	2012	feces
KF055238.1	CC024	China	2010	throat swab
KF055239.1	changchun028	China	2010	throat swab
KF055240.1	changchun029	China	2010	throat swab
KF055241.1	CC045	China	2010	throat swab
KF055242.1	changchun075	China	2010	throat swab
KF055243.1	CC090	China	2010	throat swab
KF055244.1	CC097	China	2010	throat swab
KF055245.1	CC163	China	2010	throat swab
KF150150.1	048/LY/CHN/AM/10/CA16	China	2010	throat swab
KF193620.1	ZJ08-01	China	2008	throat swab

KF193621.1	SD09-05	China	2009	throat swab
KF193622.1	YN10-02	China	2010	throat swab
KF193623.1	HN11-03	China	2011	throat swab
KF193624.1	HN09-02	China	2009	throat swab
KF193625.1	GX10-01	China	2010	throat swab
KF193626.1	FJ10-03	China	2010	throat swab
KF193627.1	FJ09-02	China	2009	throat swab
KF193628.1	FJ09-01	China	2009	throat swab
KF193629.1	BJ10-03	China	2010	throat swab
KF193630.1	BJ10-01	China	2010	throat swab
KF193631.1	BJ10-02	China	2010	throat swab
KF193632.1	AH08-06	China	2008	throat swab
KF748142.1	SiICRC01/TH/2011	Thailand	2011	throat swab
KF748143.1	SiICRC02/TH/2011	Thailand	2011	feces
KF748144.1	SiICRC03/TH/2011	Thailand	2011	feces
KF748146.1	SiICRC05/TH/2011	Thailand	2011	feces
KF748147.1	SiICRC06/TH/2011	Thailand	2011	feces
KF956714.1	FLA6014	Peru	2009	throat swab
KF956715.1	Piura	Peru	2008	throat swab
KF956716.1	FLA6784	Peru	2009	throat swab
KF956717.1	Iquitos	Peru	2006	throat swab
KF956718.1	FLA8485	Peru	2009	throat swab
KF956719.1	Piura	Peru	2008	throat swab
KJ156344.1	CS161/HN/CHN/12	China	2012	throat swab
KJ156345.1	CS722/HN/CHN/12	China	2012	throat swab
KJ156346.1	CS534/HN/CHN/12	China	2012	throat swab
KJ156347.1	CS425/HN/CHN/12	China	2012	throat swab
KJ156348.1	CS397/HN/CHN/12	China	2012	throat swab
KJ746492.1	L23	China	2010	throat swab
KJ784501.1	A16 CS43/HN/CHN/12	China	2012	throat swab
KJ784502.1	A16 CS49/HN/CHN/12	China	2012	throat swab
KJ784503.1	A16 CS50/HN/CHN/12	China	2012	throat swab
KJ784504.1	A16 CS51/HN/CHN/12	China	2012	throat swab
KJ784505.1	A16 CS54/HN/CHN/12	China	2012	throat swab
KJ784506.1	A16 CS69/HN/CHN/12	China	2012	throat swab
KJ784507.1	A16 CS89/HN/CHN/12	China	2012	throat swab
KJ865493.1	101896/GZ/2010/A16	China	2010	feces
KJ865494.1	104567/GZ/2010/A16	China	2010	feces
KJ865495.1	105444/GZ/2010/A16	China	2010	feces
KJ865496.1	106988/GZ/2010/A16	China	2010	feces
KJ865497.1	109643/GZ/2010/A16	China	2010	feces
KJ865498.1	109831/GZ/2010/A16	China	2010	feces
KJ865499.1	112591/GZ/2011/A16	China	2011	feces

KJ865500.1	113925/GZ/2011/A16	China	2011	feces
KJ865501.1	115559/GZ/2011/A16	China	2011	feces
KJ865502.1	117552/GZ/2011/A16	China	2011	feces
KJ865503.1	118569/GZ/2011/A16	China	2011	feces
KJ865504.1	119809/GZ/2011/A16	China	2011	feces
KJ865505.1	123318/GZ/2012/A16	China	2012	feces
KJ865506.1	129177/GZ/2012/A16	China	2012	feces
KJ865507.1	135870/GZ/2013/A16	China	2013	feces
KJ865508.1	1010849/GZ/2010/A16	China	2010	feces
KJ865509.1	1012669/GZ/2010/A16	China	2010	feces
KJ865510.1	1310922/GZ/2013/A16	China	2013	feces
KJ865511.1	1325392/GZ/2013/A16	China	2013	feces
KJ865512.1	1329652/GZ/2013/A16	China	2013	feces
KJ865513.1	1334530/GZ/2013/A16	China	2013	feces
KM215267.1	CVA16/SZ29/CHN/2014	China	2014	feces
KM675923.1	SiICRC02/TH/2012	Thailand	2012	feces
KM675924.1	SiICRC03/TH/2012	Thailand	2012	feces
KM675925.1	SiICRC01/TH/2014	Thailand	2014	throat swab
KP005836.1	JB141330149-CA16	China	2013	anal swab
KP005840.1	JB141330230-CA16	China	2013	feces
KP289411.1	CV-A16/P10/2013/China	China	2013	feces
KP289412.1	CV-A16/P1014/2013/China	China	2013	feces
KP289413.1	CV-A16/P187/2013/China	China	2013	feces
KP289414.1	CV-A16/P255/2013/China	China	2013	feces
KP289415.1	CV-A16/P301/2013/China	China	2013	feces
KP289416.1	CV-A16/P83/2013/China	China	2013	feces
KP751450.1	BX13-006/NJ/CHN/2013	China	2013	throat swab
KP751451.1	BX13-011/NJ/CHN/2013	China	2013	throat swab
KP751452.1	BX13-029/NJ/CHN/2013	China	2013	throat swab
KP751453.1	DC13-012/NJ/CHN/2013	China	2013	throat swab
KP751454.1	DC13-035/NJ/CHN/2013	China	2013	throat swab
KP751455.1	GC13-003/NJ/CHN/2013	China	2013	throat swab
KP751456.1	GC13-005/NJ/CHN/2013	China	2013	throat swab
KP751457.1	JN13-007/NJ/CHN/2013	China	2013	throat swab
KP751458.1	JN13-009/NJ/CHN/2013	China	2013	throat swab
KP751459.1	JN13-016/NJ/CHN/2013	China	2013	throat swab
KP751460.1	JY13-029/NJ/CHN/2013	China	2013	throat swab
KP751461.1	LS13-014/NJ/CHN/2013	China	2013	throat swab
KP751462.1	PK13-026/NJ/CHN/2013	China	2013	throat swab
KP751463.1	QH13-015/NJ/CHN/2013	China	2013	throat swab
KP751464.1	QX13-031/NJ/CHN/2013	China	2013	throat swab
KP751465.1	XW13-005/NJ/CHN/2013	China	2013	throat swab
KP751466.1	XW13-011/NJ/CHN/2013	China	2013	throat swab

KP751467.1	XW13-021/NJ/CHN/2013	China	2013	throat swab
KP751468.1	YH13-008/NJ/CHN/2013	China	2013	throat swab
KP751469.1	YH13-035/NJ/CHN/2013	China	2013	throat swab
KP751470.1	XW13-038/NJ/CHN/2013	China	2013	throat swab
KP751471.1	JN13-036/NJ/CHN/2013	China	2013	throat swab
KP751472.1	JY13-035/NJ/CHN/2013	China	2013	throat swab
KP751473.1	GC13-049/NJ/CHN/2013	China	2013	throat swab
KP751474.1	GC13-053/NJ/CHN/2013	China	2013	throat swab
KP751475.1	GC13-054/NJ/CHN/2013	China	2013	throat swab
KP751476.1	XW13-044/NJ/CHN/2013	China	2013	throat swab
KP751477.1	QH13-056/NJ/CHN/2013	China	2013	throat swab
KP751478.1	QH13-057/NJ/CHN/2013	China	2013	throat swab
KP751479.1	QH13-059/NJ/CHN/2013	China	2013	throat swab
KP751480.1	JY13-036/NJ/CHN/2013	China	2013	throat swab
KP751481.1	DC13-067/NJ/CHN/2013	China	2013	throat swab
KP751482.1	NJ12-018/NJ/CHN/2012	China	2012	throat swab
KP751483.1	NJ12-089/NJ/CHN/2012	China	2012	throat swab
KP751484.1	NJ12-111/NJ/CHN/2012	China	2012	throat swab
KP751485.1	NJ12-112/NJ/CHN/2012	China	2012	throat swab
KP751486.1	NJ12-113/NJ/CHN/2012	China	2012	throat swab
KP751487.1	NJ12-124/NJ/CHN/2012	China	2012	throat swab
KP751488.1	NJ12-135/NJ/CHN/2012	China	2012	throat swab
KP751489.1	NJ12-154/NJ/CHN/2012	China	2012	throat swab
KP751490.1	NJ12-166/NJ/CHN/2012	China	2012	throat swab
KP751491.1	NJ12-383/NJ/CHN/2012	China	2012	throat swab
KP751492.1	NJ12-384/NJ/CHN/2012	China	2012	throat swab
KP751493.1	NJ12-389/NJ/CHN/2012	China	2012	throat swab
KP751494.1	NJ12-390/NJ/CHN/2012	China	2012	throat swab
KP751496.1	NJ12-395/NJ/CHN/2012	China	2012	throat swab
KP751497.1	NJ12-396/NJ/CHN/2012	China	2012	throat swab
KP751499.1	NJ12-398/NJ/CHN/2012	China	2012	throat swab
KP751501.1	XW12-001/NJ/CHN/2012	China	2012	throat swab
KP751502.1	BX12-001/NJ/CHN/2012	China	2012	throat swab
KP751503.1	QH12-005/NJ/CHN/2012	China	2012	throat swab
KP751504.1	JY12-005/NJ/CHN/2012	China	2012	throat swab
KP751505.1	JN12-004/NJ/CHN/2012	China	2012	throat swab
KP751506.1	LH12-004/NJ/CHN/2012	China	2012	throat swab
KP751507.1	XW12-006/NJ/CHN/2012	China	2012	throat swab
KP751508.1	BX12-008/NJ/CHN/2012	China	2012	throat swab
KP751509.1	GL12-008/NJ/CHN/2012	China	2012	throat swab
KP751510.1	JY13-010/NJ/CHN/2013	China	2013	throat swab
KP751511.1	JY13-013/NJ/CHN/2013	China	2013	throat swab
KP751512.1	QX13-034/NJ/CHN/2013	China	2013	throat swab

KP751513.1	JN13-035/NJ/CHN/2013	China	2013	throat swab
KP751514.1	PK13-005/NJ/CHN/2013	China	2013	throat swab
KP751516.1	NJ12-114/NJ/CHN/2012	China	2012	throat swab
KP751518.1	LS12-005/NJ/CHN/2012	China	2012	throat swab
KP751519.1	PK12-006/NJ/CHN/2012	China	2012	throat swab
KP751520.1	DC12-007/NJ/CHN/2012	China	2012	throat swab
KP751521.1	LH12-009/NJ/CHN/2012	China	2012	throat swab
KP751522.1	JY12-011/NJ/CHN/2012	China	2012	throat swab
KP751523.1	PK12-014/NJ/CHN/2012	China	2012	throat swab
KP751524.1	QX12-012/NJ/CHN/2012	China	2012	throat swab
KP751525.1	YH12-008/NJ/CHN/2012	China	2012	throat swab
KP751526.1	JN12-014/NJ/CHN/2012	China	2012	throat swab
KP751527.1	LH12-015/NJ/CHN/2012	China	2012	throat swab
KP751530.1	XW12-009/NJ/CHN/2012	China	2012	throat swab
KP751531.1	BX12-012/NJ/CHN/2012	China	2012	throat swab
KP751532.1	JY12-016/NJ/CHN/2012	China	2012	throat swab
KP751533.1	GL12-022/NJ/CHN/2012	China	2012	throat swab
KP751535.1	DC12-017/NJ/CHN/2012	China	2012	throat swab
KP751536.1	QX12-019/NJ/CHN/2012	China	2012	throat swab
KP751538.1	JN12-020/NJ/CHN/2012	China	2012	throat swab
KP751540.1	XW12-018/NJ/CHN/2012	China	2012	throat swab
KP751541.1	BX12-022/NJ/CHN/2012	China	2012	throat swab
KP751542.1	QH12-021/NJ/CHN/2012	China	2012	throat swab
KP751543.1	JY12-023/NJ/CHN/2012	China	2012	throat swab
KP751544.1	GL12-028/NJ/CHN/2012	China	2012	throat swab
KP751545.1	QX12-025/NJ/CHN/2012	China	2012	throat swab
KP751546.1	YH12-020/NJ/CHN/2012	China	2012	throat swab
KP751547.1	JN12-023/NJ/CHN/2012	China	2012	throat swab
KP751548.1	LH12-021/NJ/CHN/2012	China	2012	throat swab
KP751549.1	LS12-025/NJ/CHN/2012	China	2012	throat swab
KP751550.1	XW12-031/NJ/CHN/2012	China	2012	throat swab
KP751551.1	BX12-032/NJ/CHN/2012	China	2012	throat swab
KP751553.1	QH12-035/NJ/CHN/2012	China	2012	throat swab
KP751554.1	GL12-037/NJ/CHN/2012	China	2012	throat swab
KP751555.1	GL12-039/NJ/CHN/2012	China	2012	throat swab
KP751556.1	DC12-034/NJ/CHN/2012	China	2012	throat swab
KP751557.1	QX12-031/NJ/CHN/2012	China	2012	throat swab
KP751558.1	YH12-030/NJ/CHN/2012	China	2012	throat swab
KP751559.1	JN12-031/NJ/CHN/2012	China	2012	throat swab
KP751560.1	LS12-034/NJ/CHN/2012	China	2012	throat swab
KP751561.1	GC12-032/NJ/CHN/2012	China	2012	throat swab
KP751562.1	BX12-045/NJ/CHN/2012	China	2012	throat swab
KP751563.1	JY12-043/NJ/CHN/2012	China	2012	throat swab

KP751564.1	DC12-045/NJ/CHN/2012	China	2012	throat swab
KP751565.1	YH12-035/NJ/CHN/2012	China	2012	throat swab
KP751566.1	JN12-044/NJ/CHN/2012	China	2012	throat swab
KP751567.1	DC12-052/NJ/CHN/2012	China	2012	throat swab
KP751568.1	LH12-046/NJ/CHN/2012	China	2012	throat swab
KP751569.1	XW12-036/NJ/CHN/2012	China	2012	throat swab
KP751570.1	GL12-060/NJ/CHN/2012	China	2012	throat swab
KP751571.1	QX12-051/NJ/CHN/2012	China	2012	throat swab
KP751572.1	QX12-057/NJ/CHN/2012	China	2012	throat swab
KP751573.1	YH12-046/NJ/CHN/2012	China	2012	throat swab
KP751574.1	LS12-050/NJ/CHN/2012	China	2012	throat swab
KP751575.1	GC12-056/NJ/CHN/2012	China	2012	throat swab
KP751576.1	YH12-052/NJ/CHN/2012	China	2012	throat swab
KP751578.1	NJ11-747/NJ/CHN/2011	China	2011	throat swab
KP751579.1	LS11-001/NJ/CHN/2011	China	2011	throat swab
KP751581.1	BX11-008/NJ/CHN/2011	China	2011	throat swab
KP751582.1	DC11-011/NJ/CHN/2011	China	2011	throat swab
KP751583.1	GC11-013/NJ/CHN/2011	China	2011	throat swab
KP751584.1	JN11-017/NJ/CHN/2011	China	2011	throat swab
KP751585.1	GC11-022/NJ/CHN/2011	China	2011	throat swab
KP751586.1	GC11-027/NJ/CHN/2011	China	2011	throat swab
KP751587.1	GC11-029/NJ/CHN/2011	China	2011	throat swab
KP751588.1	PK11-001/NJ/CHN/2011	China	2011	throat swab
KP751589.1	QX11-013/NJ/CHN/2011	China	2011	throat swab
KP751590.1	BX11-018/NJ/CHN/2011	China	2011	throat swab
KP751591.1	YH11-016/NJ/CHN/2011	China	2011	throat swab
KP751592.1	BX11-033/NJ/CHN/2011	China	2011	throat swab
KP751593.1	JY11-027/NJ/CHN/2011	China	2011	throat swab
KP751594.1	GL11-052/NJ/CHN/2011	China	2011	throat swab
KP751595.1	DC11-033/NJ/CHN/2011	China	2011	throat swab
KP751596.1	QX11-030/NJ/CHN/2011	China	2011	throat swab
KP751597.1	YH11-027/NJ/CHN/2011	China	2011	throat swab
KP751598.1	JN11-036/NJ/CHN/2011	China	2011	throat swab
KP751599.1	XW11-019/NJ/CHN/2011	China	2011	throat swab
KP751600.1	XW11-023/NJ/CHN/2011	China	2011	throat swab
KP751601.1	PK11-023/NJ/CHN/2011	China	2011	throat swab
KP751602.1	JN11-042/NJ/CHN/2011	China	2011	throat swab
KP751605.1	QH11-048/NJ/CHN/2011	China	2011	throat swab
KP751606.1	QH11-050/NJ/CHN/2011	China	2011	throat swab
KP751607.1	JY11-034/NJ/CHN/2011	China	2011	throat swab
KP751608.1	GL11-062/NJ/CHN/2011	China	2011	throat swab
KP751610.1	LH11-045/NJ/CHN/2011	China	2011	throat swab
KP751611.1	PK11-036/NJ/CHN/2011	China	2011	throat swab

KP751612.1	QX11-050/NJ/CHN/2011	China	2011	throat swab
KP751613.1	LH11-052/NJ/CHN/2011	China	2011	throat swab
KP751614.1	XW11-036/NJ/CHN/2011	China	2011	throat swab
KP751615.1	JY11-051/NJ/CHN/2011	China	2011	throat swab
KP751616.1	PK11-040/NJ/CHN/2011	China	2011	throat swab
KP751617.1	JN11-058/NJ/CHN/2011	China	2011	throat swab
KP751619.1	DC11-020/NJ/CHN/2011	China	2011	throat swab
KP751620.1	DC13-005/NJ/CHN/2013	China	2013	throat swab
KP751621.1	GL11-057/NJ/CHN/2011	China	2011	throat swab
KP751622.1	JY12-008/NJ/CHN/2012	China	2012	throat swab
KP751623.1	LH11-023/NJ/CHN/2011	China	2011	throat swab
KP751624.1	QX11-040/NJ/CHN/2011	China	2011	throat swab
KR138313.1	lianyungang-1-2011	China	2011	throat swab
KR138314.1	lianyungang-2-2011	China	2011	throat swab
KR138315.1	lianyungang-3-2011	China	2011	throat swab
KR138316.1	lianyungang-4-2011	China	2011	throat swab
KR138317.1	zhenjiang-2-2011	China	2011	throat swab
KR138318.1	changzhou-1-2012	China	2012	throat swab
KR138319.1	huaian-1-2012	China	2012	throat swab
KR138320.1	lianyungang-1-2012	China	2012	throat swab
KR138321.1	lianyungang-2-2012	China	2012	throat swab
KR138322.1	nanjing-1-2012	China	2012	throat swab
KR138323.1	nanjing-2-2012	China	2012	throat swab
KR138324.1	nantong-1-2012	China	2012	throat swab
KR138325.1	nantong-2-2012	China	2012	throat swab
KR138326.1	suzhou-1-2012	China	2012	throat swab
KR138327.1	wuxi-1-2012	China	2012	throat swab
KR138328.1	wuxi-2-2012	China	2012	throat swab
KR138329.1	wuxi-3-2012	China	2012	throat swab
KR138330.1	wuxi-4-2012	China	2012	throat swab
KR138331.1	xuzhou-1-2012	China	2012	throat swab
KR138332.1	xuzhou-2-2012	China	2012	throat swab
KR138333.1	xuzhou-3-2012	China	2012	throat swab
KR138334.1	xuzhou-4-2012	China	2012	throat swab
KR138335.1	yancheng-1-2012	China	2012	throat swab
KR138336.1	yancheng-2-2012	China	2012	throat swab
KR138337.1	yancheng-3-2012	China	2012	throat swab
KR138338.1	yancheng-4-2012	China	2012	throat swab
KR138339.1	yangchou-1-2012	China	2012	throat swab
KR138340.1	yangchou-2-2012	China	2012	throat swab
KR138341.1	changzhou-1-2013	China	2013	throat swab
KR138342.1	changzhou-2-2013	China	2013	throat swab
KR138343.1	huaian-2-2013	China	2013	throat swab

KR138344.1	nanjing-1-2013	China	2013	throat swab
KR138346.1	suzhou-1-2013	China	2013	throat swab
KR138347.1	suzhou-2-2013	China	2013	throat swab
KR138348.1	wuxi-1-2013	China	2013	throat swab
KR138349.1	wuxi-2-2013	China	2013	throat swab
KR138350.1	wuxi-3-2013	China	2013	throat swab
KR138351.1	wuxi-4-2013	China	2013	throat swab
KR138352.1	yancheng-1-2013	China	2013	throat swab
KR138353.1	yancheng-2-2013	China	2013	throat swab
KR138354.1	yangzhou-1-2013	China	2013	throat swab
KR138355.1	yangzhou-2-2013	China	2013	throat swab
KR138356.1	yangzhou-3-2013	China	2013	throat swab
KR138357.1	zhenjiang-1-2013	China	2013	throat swab
KR138358.1	changzhou-1-2014	China	2014	throat swab
KR138359.1	changzhou-2-2014	China	2014	throat swab
KR138360.1	lianyungang-1-2014	China	2014	throat swab
KR138361.1	nanjing-1-2014	China	2014	throat swab
KR138362.1	nanjing-2-2014	China	2014	throat swab
KR138363.1	suzhou-1-2014	China	2014	throat swab
KR138364.1	suzhou-2-2014	China	2014	throat swab
KR138365.1	taizhou-1-2014	China	2014	throat swab
KR138366.1	taizhou-2-2014	China	2014	throat swab
KR138367.1	wuxi-1-2014	China	2014	throat swab
KR138368.1	wuxi-2-2014	China	2014	throat swab
KR138369.1	wuxi-3-2014	China	2014	throat swab
KR138370.1	yancheng-1-2014	China	2014	throat swab
KR138371.1	yancheng-2-2014	China	2014	throat swab
KR138372.1	yancheng-3-2014	China	2014	throat swab
KR138373.1	yancheng-4-2014	China	2014	throat swab
KR138374.1	yancheng-5-2014	China	2014	throat swab
KR138376.1	yancheng-7-2014	China	2014	throat swab
KR138377.1	zhenjiang-1-2014	China	2014	throat swab
KT908038.1	MY-2235-12	Malaysia	2012	throat swab
KU854873.1	CA16-193	China	2008	vesicle swab
KX056216.1	CA16-194	China	2008	vesicle swab
KX058533.1	ensh01-CHN-12	China	2012	throat swab
KX058534.1	Wh127-CHN-11	China	2011	throat swab
KX058535.1	Wh119-CHN-11	China	2011	throat swab
KX058536.1	Wh97-CHN-11	China	2011	throat swab
KX058537.1	Wh91-CHN-11	China	2011	throat swab
KX058538.1	Wh39-CHN-10	China	2010	throat swab
KX058539.1	ensh30-CHN-11	China	2011	throat swab
KX058540.1	ensh14-CHN-10	China	2010	throat swab

KX372336.1	SiICRC01/TH/2012	Thailand	2012	throat swab
KX372337.1	SiICRC02/TH/2012	Thailand	2012	feces
KX372338.1	SiICRC03/TH/2012	Thailand	2012	feces
KX372339.1	SiICRC01/TH/2014	Thailand	2014	throat swab
KX529822.1	YT072F	China	2015	throat swab
KX529823.1	YT244F	China	2015	throat swab
KX529824.1	YT245F	China	2015	throat swab
KX529825.1	YT267F	China	2015	throat swab
KX529826.1	YT268F	China	2015	throat swab
KX529827.1	YT277F	China	2015	throat swab
KX529828.1	YT280F	China	2015	throat swab
KX529829.1	YT283F	China	2015	throat swab
KX529830.1	YT286F	China	2015	throat swab
KX529831.1	YT424F	China	2015	throat swab
KX580041.1	CA16-196	China	2008	vesicle swab
KX586331.1	2012-R129-YT0629-CVA16(B1b)	China	2012	throat swab
KX586332.1	2012-R131-YT0628-CVA16(B1b)	China	2012	throat swab
KX586333.1	2012-R132-YT0622-CVA16(B1b)	China	2012	throat swab
KX586334.1	2012-R133-YT0637-CVA16(B1a)	China	2012	throat swab
KX586335.1	2012-R135-YT0645-CVA16(B1b)	China	2012	throat swab
KX586336.1	2012-R137-YT630-CVA16(B1b)	China	2012	throat swab
KX586338.1	2012-R140-YT0628-CVA16(B1b)	China	2012	throat swab
KX586340.1	2011-YT45-VP1-CVA16-B1b	China	2011	throat swab
KX586341.1	2011-YT52-VP1-CVA16-B1b	China	2011	throat swab
KX586342.1	2013-YT54F-CVA16	China	2013	throat swab
KX586343.1	2013-YT57F-CVA16	China	2013	throat swab
KX586346.1	2013-YT62F-VP1-CVA16-B1b_(1)	China	2013	throat swab
KX586347.1	2013-YT62F-VP1-CVA16-B1b_(2)	China	2013	throat swab
KX586348.1	2014-YT060-CV-A16(B1b)	China	2014	throat swab
KX586349.1	2014-YT148-CV-A16(B1b)	China	2014	throat swab
KX586350.1	2014-YT154-CV-A16(B1b)	China	2014	throat swab
KX586351.1	2014-YT301-CV-A16(B1b)	China	2014	throat swab
KX586352.1	2014-YT309-CV-A16(B1a)	China	2014	throat swab
KX595291.1	CVA16/Shenzhen36/CHN/2014	China	2014	feces
KX595292.1	CVA16/Shenzhen73/CHN/2014	China	2014	feces
KX595293.1	CVA16/Shenzhen74/CHN/2014	China	2014	feces
KX595294.1	CVA16/Shenzhen79/CHN/2014	China	2014	feces
KX595295.1	CVA16/Shenzhen179/CHN/2014	China	2014	feces
KX601689.1	2011-YT52-VP1-CVA16-B1b	China	2011	throat swab
KY014077.1	393	China	2008	vesicle swab
KY088084.1	K168/8	China	2010	throat swab
KY425528.1	K11/YN/CHN/2011	China	2011	feces
KY425529.1	V1/YN/CHN/2015	China	2015	feces

KY425530.1	R5/YN/CHN/2011	China	2011	feces
KY425531.1	K34/YN/CHN/2011	China	2011	feces
KY425532.1	R68/YN/CHN/2009	China	2009	feces
KY425533.1	R35/YN/CHN/2012	China	2012	feces
KY425534.1	R34/YN/CHN/2012	China	2012	feces
KY425535.1	R37/YN/CHN/2013	China	2013	feces
KY425536.1	V37/YN/CHN/2014	China	2014	feces
KY425537.1	R141/YN/CHN/2009	China	2009	feces
KY425538.1	R254/YN/CHN/2010	China	2010	feces
KY425539.1	R255/YN/CHN/2010	China	2010	feces
KY425540.1	V86/YN/CHN/2015	China	2015	feces
KY497222.1	km15-23G	China	2015	anal swab
KY497223.1	km15-27Y	China	2015	throat swab
KY497224.1	km15-31Y	China	2015	throat swab
KY497225.1	km15-32Y	China	2015	throat swab
KY497226.1	km15-32G	China	2015	anal swab
KY497227.1	km15-43Y	China	2015	throat swab
KY497228.1	km15-43G	China	2015	anal swab
KY497229.1	km15-73Y	China	2015	throat swab
KY497230.1	km15-73G	China	2015	anal swab
KY792576.1	CV-A16-A01-BLR-IN	India	2012	feces
KY792577.1	CV-A16-A02-BLR-IN	India	2012	feces
KY792578.1	CV-A16-A06-BLR-IN	India	2013	feces
KY792579.1	CV-A16-A10-BLR-IN	India	2013	feces
KY792580.1	CV-A16-A13-BLR-IN	India	2013	feces
KY792581.1	CV-A16-A122-BLR-IN	India	2015	feces
KY792582.1	CV-A16-A128-BLR-IN	India	2015	feces
KY792583.1	CV-A16-M02-BLR-IN	India	2013	feces
KY792584.1	CV-A16-M69-BLR-IN	India	2015	feces
KY796106.1	NIV1322502/CVA-16/2013/Pune/India	India	2013	vesicle swab
KY796107.1	NIV1321823/CVA-16/2013/Pune/India	India	2013	anal swab
KY796108.1	NIV1322107/CVA-16/2013/Pune/India	India	2013	throat swab
KY796109.1	NIV1322374/CVA-16/2013/Pune/India	India	2013	vesicle swab
KY796110.1	NIV1322102/CVA-16/2013/Pune/India	India	2013	anal swab
KY796111.1	NIV1321420/CVA-16/2013/Pune/India	India	2013	vesicle swab
KY796112.1	NIV1321825/CVA-16/2013/Pune/India	India	2013	throat swab
KY796113.1	NIV1322365/CVA-16/2013/Pune/India	India	2013	vesicle swab
KY796114.1	NIV1321819/CVA-16/2013/Pune/India	India	2013	feces
KY796115.1	NIV1321433/CVA-16/2013/Pune/India	India	2013	anal swab
KY796116.1	NIV1320584/CVA-16/2013/Pune/India	India	2013	anal swab
KY796117.1	NIV1320588/CVA-16/2013/Pune/India	India	2013	throat swab
KY796118.1	NIV1320913/CVA-16/2013/Pune/India	India	2013	throat swab
KY796119.1	NIV1321824/CVA-16/2013/Pune/India	India	2013	vesicle swab

KY796120.1	NIV1322368/CVA-16/2013/Pune/India	India	2013	vesicle swab
KY796121.1	NIV1323503/CVA-16/2013/Pune/India	India	2013	vesicle swab
KY796122.1	NIV1323507/CVA-16/2013/Pune/India	India	2013	throat swab
KY796123.1	NIV1323513/CVA-16/2013/Pune/India	India	2013	vesicle swab
KY796124.1	NIV1324322/CVA-16/2013/Pune/India	India	2013	vesicle swab
KY796125.1	NIV1321821/CVA-16/2013/Pune/India	India	2013	vesicle swab
KY796126.1	NIV1322369/CVA-16/2013/Pune/India	India	2013	throat swab
KY796127.1	NIV1323499/CVA-16/2013/Pune/India	India	2013	vesicle swab
KY796128.1	NIV1322716/CVA-16/2013/Pune/India	India	2013	throat swab
KY796129.1	NIV1321648/CVA-16/2013/Pune/India	India	2013	vesicle swab
KY796130.1	NIV1321649/CVA-16/2013/Pune/India	India	2013	throat swab
KY796132.1	NIV1321466/CVA-16/2013/Pune/India	India	2013	anal swab
KY796133.1	NIV1321460/CVA-16/2013/Pune/India	India	2013	vesicle swab
KY796134.1	NIV1321436/CVA-16/2013/Pune/India	India	2013	anal swab
KY796135.1	NIV1321437/CVA-16/2013/Pune/India	India	2013	throat swab
KY796136.1	NIV1211484/CVA-16/2012/Ahmadabad/India	India	2012	vesicle swab
KY796137.1	NIV1211481/CVA-16/2012/Ahmadabad/India	India	2012	vesicle swab
KY796140.1	NIV1211438/CVA-16/2012/Ahmadabad/India	India	2012	vesicle swab
KY796141.1	NIV1211445/CVA-16/2012/Ahmadabad/India	India	2012	feces
KY796142.1	NIV0916024/CVA-16/2009/West Bengal/India	India	2009	feces
KY796143.1	NIV0924309/CVA-16/2009/West Bengal/India	India	2009	vesicle swab
KY796144.1	NIV0916022/CVA-16/2009/West Bengal/India	India	2009	vesicle swab
KY796145.1	NIV0924312/CVA-16/2009/West Bengal/India	India	2009	throat swab
KY796146.1	NIV0924311/CVA-16/2009/West Bengal/India	India	2009	vesicle swab
KY796148.1	NIV0915920/CVA-16/2009/West Bengal/India	India	2009	vesicle swab
KY796149.1	NIV0104441/CVA-16/2010/Tamil Nadu/India	India	2010	vesicle swab
KY796150.1	NIV104433/CVA-16/2010/Tamil Nadu/India	India	2010	vesicle swab
KY796151.1	NIV104435/CVA-16/2010/Tamil Nadu/India	India	2010	vesicle swab
KY796154.1	NIV1050768/CVA-16/2010/Kerala/India	India	2010	feces
KY796155.1	NIV1050767/CVA-16/2010/Kerala/India	India	2010	feces
LC012359.1	3-C4-YN-CHN-2012	China	2012	feces

LC012360.1	7-C9-YN-CHN-2012	China	2012	feces
LC012361.1	10-C18-YN-CHN-2012	China	2012	feces
LC012362.1	11-C22-YN-CHN-2012	China	2012	feces
LC012363.1	13-C26-YN-CHN-2012	China	2012	feces
LC012364.1	14-C32-YN-CHN-2012	China	2012	feces
LC012365.1	15-C33-YN-CHN-2012	China	2012	feces
LC012366.1	16-C34-YN-CHN-2012	China	2012	feces
LC012367.1	17-C35-YN-CHN-2012	China	2012	feces
LC012368.1	18-C37-YN-CHN-2012	China	2012	feces
LC012369.1	19-C41-YN-CHN-2012	China	2012	feces
LC012370.1	23-C47-YN-CHN-2012	China	2012	feces
LC012374.1	27-C66-YN-CHN-2012	China	2012	feces
LC012375.1	28-C67-YN-CHN-2012	China	2012	feces
LC012376.1	29-C68-YN-CHN-2012	China	2012	feces
LC012377.1	30-C70-YN-CHN-2012	China	2012	feces
LC012378.1	32-C76-YN-CHN-2012	China	2012	feces
LC012379.1	33-C79-YN-CHN-2012	China	2012	feces
LC012380.1	34-C81-YN-CHN-2012	China	2012	feces
LC012382.1	37-C85-YN-CHN-2012	China	2012	feces
LC012384.1	41-C90-YN-CHN-2012	China	2012	feces
LC012385.1	42-C92-YN-CHN-2012	China	2012	feces
LC012386.1	43-C94-YN-CHN-2012	China	2012	feces
LC012387.1	44-C95-YN-CHN-2012	China	2012	feces
LC012390.1	47-C99-YN-CHN-2012	China	2012	feces
LC012391.1	48-C100-YN-CHN-2012	China	2012	feces
LC012393.1	51-C105-YN-CHN-2012	China	2012	feces
LC012394.1	52-C106-YN-CHN-2012	China	2012	feces
LC012395.1	53-C107-YN-CHN-2012	China	2012	feces
LC012397.1	55-C112-YN-CHN-2012	China	2012	feces
LC012398.1	57-C114-YN-CHN-2012	China	2012	feces
LC012399.1	58-C116-YN-CHN-2012	China	2012	feces
LC012400.1	59-C117-YN-CHN-2012	China	2012	feces
LC012401.1	60-C118-YN-CHN-2012	China	2012	feces
LC012402.1	61-C119-YN-CHN-2012	China	2012	feces
LC012403.1	62-C122-YN-CHN-2012	China	2012	feces
LC012404.1	63-C123-YN-CHN-2012	China	2012	feces
LC012405.1	64-C124-YN-CHN-2012	China	2012	feces
LC012407.1	68-C134-YN-CHN-2012	China	2012	feces
LC012408.1	69-D10-YN-CHN-2012	China	2012	feces
LC012409.1	70-D13-YN-CHN-2012	China	2012	feces
LC012410.1	71-D14-YN-CHN-2012	China	2012	feces
LC012412.1	73-D17-YN-CHN-2012	China	2012	feces
LC012415.1	76-D21-YN-CHN-2012	China	2012	feces

LC012417.1	79-D24-YN-CHN-2012	China	2012	feces
LC012418.1	80-D25-YN-CHN-2012	China	2012	feces
LC012419.1	81-D26-YN-CHN-2012	China	2012	feces
LC012420.1	82-D31-YN-CHN-2012	China	2012	feces
LC012421.1	83-D32-YN-CHN-2012	China	2012	feces
LC012422.1	84-D33-YN-CHN-2012	China	2012	feces
LC012427.1	89-D40-YN-CHN-2012	China	2012	feces
LC012429.1	91-D42-YN-CHN-2012	China	2012	feces
LC012430.1	92-D44-YN-CHN-2012	China	2012	feces
LC012431.1	93-D45-YN-CHN-2012	China	2012	feces
LC012437.1	99-D59-YN-CHN-2012	China	2012	feces
LC012439.1	101-A1-YN-CHN-2012	China	2012	feces
LC012440.1	106-A10-YN-CHN-2012	China	2012	feces
LC012441.1	113-A20-YN-CHN-2012	China	2012	feces
LC012442.1	114-A22-YN-CHN-2012	China	2012	feces
LC012443.1	115-A23-YN-CHN-2012	China	2012	feces
LC012444.1	117-A26-YN-CHN-2012	China	2012	feces
LC012445.1	120-A32-YN-CHN-2012	China	2012	feces
LC012446.1	122-A34-YN-CHN-2012	China	2012	feces
LC012447.1	126-A40-YN-CHN-2012	China	2012	feces
LC012449.1	128-A42-YN-CHN-2012	China	2012	feces
LC012450.1	130-A46-YN-CHN-2012	China	2012	feces
LC012451.1	135-A54-YN-CHN-2012	China	2012	feces
LC012452.1	136-A55-YN-CHN-2012	China	2012	feces
LC012453.1	137-A56-YN-CHN-2012	China	2012	feces
LC012455.1	144-A68-YN-CHN-2012	China	2012	feces
LC012456.1	145-A69-YN-CHN-2012	China	2012	feces
LC012457.1	146-A70-YN-CHN-2012	China	2012	feces
LC012458.1	148-A75-YN-CHN-2012	China	2012	feces
LC012459.1	150-A77-YN-CHN-2012	China	2012	feces
LC012460.1	152-A82-YN-CHN-2012	China	2012	feces
LC012462.1	158-A93-YN-CHN-2012	China	2012	feces
LC012464.1	162-A97-YN-CHN-2012	China	2012	feces
LC012466.1	168-R8-YN-CHN-2012	China	2012	feces
LC012467.1	169-R10-YN-CHN-2012	China	2012	feces
LC012468.1	172-R20-YN-CHN-2012	China	2012	feces
LC012958.1	4-YN-CHN-2013	China	2013	feces
LC012959.1	5-YN-CHN-2013	China	2013	feces
LC012960.1	6-YN-CHN-2013	China	2013	feces
LC012961.1	9-YN-CHN-2013	China	2013	feces
LC012962.1	16-YN-CHN-2013	China	2013	feces
LC012963.1	17-YN-CHN-2013	China	2013	feces
LC012964.1	18-YN-CHN-2013	China	2013	feces

LC012965.1	19-YN-CHN-2013	China	2013	feces
LC012966.1	20-YN-CHN-2013	China	2013	feces
LC012967.1	33-YN-CHN-2013	China	2013	feces
LC012968.1	34-YN-CHN-2013	China	2013	feces
LC012969.1	35-YN-CHN-2013	China	2013	feces
LC012970.1	C6-YN-CHN-2013	China	2013	feces
LC012971.1	N23-YN-CHN-2013	China	2013	feces
LC013391.1	A19-YN-CHN-2014	China	2014	feces
LC013392.1	A22-YN-CHN-2014	China	2014	feces
LC013393.1	C14-YN-CHN-2014	China	2014	feces
LC013394.1	C37-YN-CHN-2014	China	2014	feces
LC013395.1	C46-YN-CHN-2014	China	2014	feces
LC013396.1	C48-YN-CHN-2014	China	2014	feces
LC013397.1	C50-YN-CHN-2014	China	2014	feces
LC013398.1	C52-YN-CHN-2014	China	2014	feces
LC013399.1	C55-YN-CHN-2014	China	2014	feces
LC013400.1	C67-YN-CHN-2014	China	2014	feces
LC013401.1	J14-YN-CHN-2014	China	2014	feces
LC013402.1	J31-YN-CHN-2014	China	2014	feces
LC013403.1	J36-YN-CHN-2014	China	2014	feces
LC013404.1	J46-YN-CHN-2014	China	2014	feces
LC013406.1	J82-YN-CHN-2014	China	2014	feces
LC013407.1	J85-YN-CHN-2014	China	2014	feces
LC013408.1	M18-YN-CHN-2014	China	2014	feces
LC013409.1	M89-YN-CHN-2014	China	2014	feces
LC013410.1	R8-YN-CHN-2014	China	2014	feces
LC013411.1	R16-YN-CHN-2014	China	2014	feces
LC603075.1	164-QJ-YN-CHN-2019-CV-A16	China	2019	feces
LC603076.1	178-QJ-YN-CHN-2019-CV-A16	China	2019	feces
LC603080.1	189-QJ-YN-CHN-2019-CV-A16	China	2019	feces
LC603084.1	239-QJ-YN-CHN-2019-CV-A16	China	2019	feces
LC603085.1	254-QJ-YN-CHN-2019-CV-A16	China	2019	feces
LC603088.1	256-QJ-YN-CHN-2019-CV-A16	China	2019	feces
LC603091.1	294-QJ-YN-CHN-2019-CV-A16	China	2019	feces
LC603092.1	296-QJ-YN-CHN-2019-CV-A16	China	2019	feces
LC603095.1	310-QJ-YN-CHN-2019-CV-A16	China	2019	feces
LC603096.1	320-QJ-YN-CHN-2019-CV-A16	China	2019	feces
LC603099.1	330-QJ-YN-CHN-2019-CV-A16	China	2019	feces
LC603101.1	332-QJ-YN-CHN-2019-CV-A16	China	2019	feces
LC626212.1	70/QJ/YN/CHN/2020/CV-A16	China	2020	feces
LC626213.1	71/QJ/YN/CHN/2020/CV-A16	China	2020	feces
LC626214.1	73/QJ/YN/CHN/2020/CV-A16	China	2020	feces
LC626232.1	107/QJ/YN/CHN/2020/CV-A16	China	2020	feces

LC656490.1	14-WS-YN-CHN-2020	China	2020	feces
LC656498.1	62-WS-YN-CHN-2020	China	2020	feces
LC656502.1	74-WS-YN-CHN-2020	China	2020	feces
LC656503.1	76-WS-YN-CHN-2020	China	2020	feces
LC656513.1	138-WS-YN-CHN-2020	China	2020	feces
LC656515.1	168-WS-YN-CHN-2020	China	2020	feces
LC685235.1	39-WS-YN-CHN-2021-CV-A16	China	2021	feces
LC685236.1	72-WS-YN-CHN-2021-CV-A16	China	2021	feces
LC685237.1	73-WS-YN-CHN-2021-CV-A16	China	2021	feces
LC685238.1	80-WS-YN-CHN-2021-CV-A16	China	2021	feces
LC685239.1	83-WS-YN-CHN-2021-CV-A16	China	2021	feces
LC685240.1	85-WS-YN-CHN-2021-CV-A16	China	2021	feces
LC685241.1	86-WS-YN-CHN-2021-CV-A16	China	2021	feces
LC685242.1	89-WS-YN-CHN-2021-CV-A16	China	2021	feces
LC685243.1	162-WS-YN-CHN-2021-CV-A16	China	2021	feces
LC685244.1	194-WS-YN-CHN-2021-CV-A16	China	2021	feces
LC685245.1	197-WS-YN-CHN-2021-CV-A16	China	2021	feces
LC685246.1	207-WS-YN-CHN-2021-CV-A16	China	2021	feces
LC707410.1	81-QJ-YN-CHN-2021-CVA16	China	2021	feces
LC707412.1	84-QJ-YN-CHN-2021-CVA16	China	2021	feces
LC707419.1	103-QJ-YN-CHN-2021-CVA16	China	2021	feces
LC707420.1	106-QJ-YN-CHN-2021-CVA16	China	2021	feces
LC707421.1	107-QJ-YN-CHN-2021-CVA16	China	2021	feces
LC707425.1	126-QJ-YN-CHN-2021-CVA16	China	2021	feces
LC707426.1	131-QJ-YN-CHN-2021-CVA16	China	2021	feces
LC707430.1	146-QJ-YN-CHN-2021-CVA16	China	2021	feces
LC707431.1	149-QJ-YN-CHN-2021-CVA16	China	2021	feces
LC707432.1	150-QJ-YN-CHN-2021-CVA16	China	2021	feces
LC707433.1	151-QJ-YN-CHN-2021-CVA16	China	2021	feces
LC707437.1	157-QJ-YN-CHN-2021-CVA16	China	2021	feces
LC707438.1	159-QJ-YN-CHN-2021-CVA16	China	2021	feces
LC707461.1	218-QJ-YN-CHN-2021-CVA16	China	2021	feces
LC707462.1	219-QJ-YN-CHN-2021-CVA16	China	2021	feces
LC707467.1	234-QJ-YN-CHN-2021-CVA16	China	2021	feces
LT577722.1	CF165039_FRA10	France	2010	throat swab
LT577723.1	CF168005_FRA10	France	2010	throat swab
LT577724.1	CF180036_FRA10	France	2010	throat swab
LT577725.1	CF204026_FRA10	France	2010	throat swab
LT577726.1	CF230018_FRA10	France	2010	throat swab
LT577729.1	CF301047_FRA10	France	2010	throat swab
LT577730.1	CF323006_FRA10	France	2010	throat swab
LT577732.1	CF335030_FRA10	France	2010	throat swab
LT577733.1	CF341014_FRA10	France	2010	throat swab

LT577736.1	CF348012_FRA10	France	2010	throat swab
LT577737.1	CF355014_FRA10	France	2010	throat swab
LT577738.1	CF355015_FRA10	France	2010	throat swab
LT577739.1	CF361090_FRA10	France	2010	throat swab
LT577740.1	CF071018_FRA11	France	2011	throat swab
LT577741.1	CF127005_FRA11	France	2011	throat swab
LT577742.1	CF139035_FRA11	France	2011	throat swab
LT577743.1	CF159001_FRA11	France	2011	throat swab
LT577744.1	CF166088_FRA11	France	2011	throat swab
LT577745.1	CF168018_FRA11	France	2011	throat swab
LT577746.1	CF168019_FRA11	France	2011	throat swab
LT577747.1	CF172023_FRA11	France	2011	throat swab
LT577749.1	CF221066_FRA11	France	2011	throat swab
LT577750.1	CF234014_FRA11	France	2011	throat swab
LT577751.1	CF298023_FRA11	France	2011	throat swab
LT577752.1	CF298024_FRA11	France	2011	throat swab
LT577753.1	CF301003_FRA11	France	2011	throat swab
LT577754.1	CF340005_FRA11	France	2011	throat swab
LT577755.1	CF354078_FRA11	France	2011	throat swab
LT577756.1	CF111011_FRA12	France	2012	throat swab
LT577757.1	CF164014_FRA12	France	2012	throat swab
LT577758.1	CF177030_FRA12	France	2012	throat swab
LT577759.1	CF178027_FRA12	France	2012	throat swab
LT577760.1	CF178028_FRA12	France	2012	throat swab
LT577761.1	CF178038_FRA12	France	2012	throat swab
LT577762.1	CF185003_FRA12	France	2012	throat swab
LT577763.1	CF188032_FRA12	France	2012	throat swab
LT577764.1	CF188079_FRA12	France	2012	throat swab
LT577765.1	CF198037_FRA12	France	2012	throat swab
LT577766.1	CF201018_FRA12	France	2012	throat swab
LT577767.1	CF205018_FRA12	France	2012	throat swab
LT577769.1	CF205023_FRA12	France	2012	throat swab
LT577770.1	CF216001_FRA12	France	2012	throat swab
LT577771.1	CF220007_FRA12	France	2012	throat swab
LT577772.1	CF271092_FRA12	France	2012	throat swab
LT577773.1	CF278010_FRA12	France	2012	throat swab
LT577774.1	CF342004_FRA12	France	2012	throat swab
LT577775.1	CF342037_FRA12	France	2012	throat swab
LT577776.1	CF065008_FRA13	France	2013	throat swab
LT577777.1	CF078003_FRA13	France	2013	throat swab
LT577779.1	CF147023_FRA13	France	2013	throat swab
LT577780.1	CF168064_FRA13	France	2013	throat swab
LT577781.1	CF176127_FRA13	France	2013	feces

LT577782.1	CF197024_FRA13	France	2013	throat swab
LT577784.1	CF204020_FRA13	France	2013	throat swab
LT577785.1	PMB100118_FRA14	France	2014	throat swab
LT577786.1	PMB104117_FRA14	France	2014	throat swab
LT577787.1	PMB108084_FRA14	France	2014	throat swab
LT577788.1	PMB108086_FRA14	France	2014	throat swab
LT577789.1	PMB108087_FRA14	France	2014	throat swab
LT577790.1	PMB112174_FRA14	France	2014	throat swab
LT577791.1	PMB113099_FRA14	France	2014	throat swab
LT577792.1	PMB113104_FRA14	France	2014	throat swab
LT577793.1	PMB113106_FRA14	France	2014	throat swab
LT577794.1	PMB115111_FRA14	France	2014	throat swab
LT577797.1	PMB142083_FRA14	France	2014	throat swab
LT577798.1	PMB143039_FRA14	France	2014	throat swab
LT577799.1	PMB143048_FRA14	France	2014	throat swab
LT577800.1	PMB153128_FRA14	France	2014	throat swab
LT577801.1	PMB154125_FRA14	France	2014	throat swab
LT577802.1	PMB157120_FRA14	France	2014	throat swab
LT577803.1	PMB161083_FRA14	France	2014	throat swab
LT577805.1	PMB162129_FRA14	France	2014	throat swab
LT577806.1	PMB164045_FRA14	France	2014	throat swab
LT577807.1	PMB171020_FRA14	France	2014	throat swab
LT577808.1	PMB171041_FRA14	France	2014	throat swab
LT577809.1	PMB171042_FRA14	France	2014	throat swab
LT577810.1	PMB174105_FRA14	France	2014	throat swab
LT577811.1	PMB174106_FRA14	France	2014	throat swab
LT577812.1	PMB174111_FRA14	France	2014	throat swab
LT577813.1	PMB176069_FRA14	France	2014	throat swab
LT577814.1	PMB176079_FRA14	France	2014	throat swab
LT577815.1	PMB177091_FRA14	France	2014	throat swab
LT577816.1	PMB177093_FRA14	France	2014	throat swab
LT577817.1	PMB181049_FRA14	France	2014	throat swab
LT577818.1	PMB181054_FRA14	France	2014	throat swab
LT577819.1	PMB181060_FRA14	France	2014	throat swab
LT577820.1	PMB181062_FRA14	France	2014	throat swab
LT577821.1	PMB182102_FRA14	France	2014	throat swab
LT577822.1	PMB184024_FRA14	France	2014	throat swab
LT577823.1	PMB184027_FRA14	France	2014	throat swab
LT577824.1	PMB185055_FRA14	France	2014	throat swab
LT577825.1	PMB188082_FRA14	France	2014	throat swab
LT577826.1	PMB188083_FRA14	France	2014	throat swab
LT577827.1	PMB188093_FRA14	France	2014	throat swab
LT577828.1	PMB191082_FRA14	France	2014	throat swab

LT577830.1	PMB192025_FRA14	France	2014	throat swab
LT577831.1	PMB197063_FRA14	France	2014	throat swab
LT577832.1	PMB199011_FRA14	France	2014	throat swab
LT577833.1	RP188_AUT00	Austria	2000	feces
LT577834.1	RP109_AUT04	Austria	2004	feces
LT577835.1	BER169_DEU03	Germany	2003	feces
LT577836.1	BER36_DEU08	Germany	2008	feces
LT577837.1	BER426_DEU08	Germany	2008	feces
LT577838.1	STU21_DEU09	Germany	2009	feces
LT617092.1	CVA16_B_BER53-2_DEU_2003	Germany	2003	feces
LT617095.1	STU54_DEU_2008	Germany	2008	feces
LT617096.1	CVA16_B_STU7_DEU_2009	Germany	2009	feces
LT617097.1	CVA16_B_STU4_DEU_2010	Germany	2010	feces
LT617098.1	CVA16_B_CF145057_FRA_2010	France	2010	throat swab
LT617099.1	CVA16_B_CF160074_FRA_2010	France	2010	throat swab
LT617100.1	CVA16_B_CF166109_FRA_2010	France	2010	throat swab
LT617101.1	CVA16_B_CF187036_FRA_2010	France	2010	throat swab
LT617102.1	CVA16_B_CF279014_FRA_2010	France	2010	throat swab
LT617104.1	CVA16_B_CF223065_FRA_2011	France	2011	throat swab
LT617105.1	CVA16_C_CF350028_FRA_2011	France	2011	throat swab
LT617106.1	CVA16_C_CF172083_FRA_2012	France	2012	throat swab
LT617107.1	CVA16_C_CF178025_FRA_2012	France	2012	throat swab
LT617108.1	CVA16_C_CF178036_FRA_2012	France	2012	throat swab
LT617109.1	CVA16_C_CF193053_FRA_2012	France	2012	throat swab
LT617110.1	CVA16_B_CF310002_FRA_2012	France	2012	throat swab
LT617111.1	CVA16_C_PAR155055_FRA_2014	France	2014	throat swab
LT617112.1	CVA16_C_MET171023_FRA_2014	France	2014	throat swab
LT617113.1	CVA16_C_LYO171046_FRA_2014	France	2014	throat swab
LT617114.1	CVA16_C_PAR181046_FRA_2014	France	2014	throat swab
LT617115.1	CVA16_C_PAR190033_FRA_2014	France	2014	throat swab
MF434051.1	ZJ6	China	2008	vesicle swab
MF678299.1	NSW-V10-2008-CVA16	Australia	2008	feces
MF678313.1	NSW-V24-2008-CVA16	Australia	2008	feces
MF678332.1	NSW-V43-2006-CVA16	Australia	2006	feces
MF990299.1	ETH_P11/A16_2016	Ethiopia	2016	feces
MF990300.1	ETH_P12/A16_2016	Ethiopia	2016	feces
MG571832.1	V2B	Venezuela	2015	feces
MG571837.1	V4B	Venezuela	2015	feces
MG957117.1	SH-HP-16-51	China	2016	throat swab
MH003908.1	CVA16/Shenzhen11/CHN/2013	China	2013	feces
MH003909.1	CVA16/Shenzhen45/CHN/2013	China	2013	feces
MH003912.1	CVA16/Shenzhen84/CHN/2013	China	2013	feces
MH003917.1	CVA16/Shenzhen1/CHN/2014	China	2014	anal swab

MH003918.1	CVA16/Shenzhen2/CHN/2014	China	2014	anal swab
MH003919.1	CVA16/Shenzhen3/CHN/2014	China	2014	anal swab
MH003920.1	CVA16/Shenzhen7/CHN/2014	China	2014	anal swab
MH003921.1	CVA16/Shenzhen11/CHN/2014	China	2014	anal swab
MH003922.1	CVA16/Shenzhen16/CHN/2014	China	2014	anal swab
MH003923.1	CVA16/Shenzhen47/CHN/2014	China	2014	feces
MH003924.1	CVA16/Shenzhen50/CHN/2014	China	2014	feces
MH003925.1	CVA16/Shenzhen52/CHN/2014	China	2014	anal swab
MH003927.1	CVA16/Shenzhen77/CHN/2014	China	2014	anal swab
MH003928.1	CVA16/Shenzhen84/CHN/2014	China	2014	anal swab
MH003930.1	CVA16/Shenzhen103/CHN/2014	China	2014	feces
MH003931.1	CVA16/Shenzhen118/CHN/2014	China	2014	anal swab
MH003932.1	CVA16/Shenzhen126/CHN/2014	China	2014	feces
MH003933.1	CVA16/Shenzhen143/CHN/2014	China	2014	anal swab
MH003934.1	CVA16/Shenzhen147/CHN/2014	China	2014	anal swab
MH003935.1	CVA16/Shenzhen153/CHN/2014	China	2014	throat swab
MH003936.1	CVA16/Shenzhen165/CHN/2014	China	2014	feces
MH003937.1	CVA16/Shenzhen172/CHN/2014	China	2014	feces
MH003938.1	CVA16/Shenzhen195/CHN/2014	China	2014	anal swab
MH003939.1	CVA16/Shenzhen214/CHN/2014	China	2014	feces
MH003940.1	CVA16/Shenzhen232/CHN/2014	China	2014	anal swab
MH003941.1	CVA16/Shenzhen241/CHN/2014	China	2014	anal swab
MH003942.1	CVA16/Shenzhen495/CHN/2014	China	2014	feces
MH003943.1	CVA16/Shenzhen502/CHN/2014	China	2014	anal swab
MH003944.1	CVA16/Shenzhen5/CHN/2015	China	2015	anal swab
MH003945.1	CVA16/Shenzhen28/CHN/2015	China	2015	anal swab
MH003946.1	CVA16/Shenzhen35/CHN/2015	China	2015	anal swab
MH003947.1	CVA16/Shenzhen45/CHN/2015	China	2015	anal swab
MH003948.1	CVA16/Shenzhen98/CHN/2015	China	2015	feces
MH003949.1	CVA16/Shenzhen99/CHN/2015	China	2015	anal swab
MH003950.1	CVA16/Shenzhen120/CHN/2015	China	2015	anal swab
MH003951.1	CVA16/Shenzhen132/CHN/2015	China	2015	feces
MH003952.1	CVA16/Shenzhen139/CHN/2015	China	2015	feces
MH003953.1	CVA16/Shenzhen157/CHN/2015	China	2015	feces
MH003954.1	CVA16/Shenzhen184/CHN/2015	China	2015	feces
MH003955.1	CVA16/Shenzhen255/CHN/2015	China	2015	feces
MH003956.1	CVA16/Shenzhen343/CHN/2015	China	2015	anal swab
MH003957.1	CVA16/Shenzhen499/CHN/2015	China	2015	feces
MH003958.1	CVA16/Shenzhen560/CHN/2015	China	2015	anal swab
MH003959.1	CVA16/Shenzhen569/CHN/2015	China	2015	feces
MH003960.1	CVA16/Shenzhen603/CHN/2015	China	2015	anal swab
MH003961.1	CVA16/Shenzhen608/CHN/2015	China	2015	anal swab
MH003962.1	CVA16/Shenzhen611/CHN/2015	China	2015	anal swab

MH003963.1	CVA16/Shenzhen618/CHN/2015	China	2015	anal swab
MH003964.1	CVA16/Shenzhen619/CHN/2015	China	2015	anal swab
MH003966.1	CVA16/Shenzhen632/CHN/2015	China	2015	anal swab
MH003967.1	CVA16/Shenzhen54/CHN/2016	China	2016	anal swab
MH003968.1	CVA16/Shenzhen74/CHN/2016	China	2016	feces
MH003969.1	CVA16/Shenzhen84/CHN/2016	China	2016	anal swab
MH003970.1	CVA16/Shenzhen97/CHN/2016	China	2016	anal swab
MH003971.1	CVA16/Shenzhen102/CHN/2016	China	2016	anal swab
MH003972.1	CVA16/Shenzhen113/CHN/2016	China	2016	anal swab
MH003973.1	CVA16/Shenzhen143/CHN/2016	China	2016	anal swab
MH003974.1	CVA16/Shenzhen152/CHN/2016	China	2016	feces
MH003975.1	CVA16/Shenzhen182/CHN/2016	China	2016	anal swab
MH003976.1	CVA16/Shenzhen183/CHN/2016	China	2016	anal swab
MH003977.1	CVA16/Shenzhen215/CHN/2016	China	2016	feces
MH003978.1	CVA16/Shenzhen216/CHN/2016	China	2016	feces
MH003979.1	CVA16/Shenzhen230/CHN/2016	China	2016	anal swab
MH003980.1	CVA16/Shenzhen255/CHN/2016	China	2016	anal swab
MH003981.1	CVA16/Shenzhen258/CHN/2016	China	2016	anal swab
MH003982.1	CVA16/Shenzhen264/CHN/2016	China	2016	feces
MH003983.1	CVA16/Shenzhen272/CHN/2016	China	2016	anal swab
MH003984.1	CVA16/Shenzhen291/CHN/2016	China	2016	feces
MH003985.1	CVA16/Shenzhen328/CHN/2016	China	2016	anal swab
MH003986.1	CVA16/Shenzhen337/CHN/2016	China	2016	anal swab
MH003987.1	CVA16/Shenzhen345/CHN/2016	China	2016	feces
MH003988.1	CVA16/Shenzhen355/CHN/2016	China	2016	feces
MH003989.1	CVA16/Shenzhen369/CHN/2016	China	2016	feces
MH003990.1	CVA16/Shenzhen371/CHN/2016	China	2016	anal swab
MH003991.1	CVA16/Shenzhen373/CHN/2016	China	2016	feces
MH003992.1	CVA16/Shenzhen375/CHN/2016	China	2016	feces
MH003993.1	CVA16/Shenzhen383/CHN/2016	China	2016	feces
MH003994.1	CVA16/Shenzhen385/CHN/2016	China	2016	feces
MH003995.1	CVA16/Shenzhen387/CHN/2016	China	2016	anal swab
MH003996.1	CVA16/Shenzhen393/CHN/2016	China	2016	anal swab
MH003997.1	CVA16/Shenzhen398/CHN/2016	China	2016	feces
MH003998.1	CVA16/Shenzhen399/CHN/2016	China	2016	feces
MH004000.1	CVA16/Shenzhen424/CHN/2016	China	2016	feces
MH004001.1	CVA16/Shenzhen434/CHN/2016	China	2016	feces
MH004002.1	CVA16/Shenzhen446/CHN/2016	China	2016	anal swab
MH004003.1	CVA16/Shenzhen450/CHN/2016	China	2016	feces
MH004004.1	CVA16/Shenzhen475/CHN/2016	China	2016	anal swab
MH004007.1	CVA16/Shenzhen494/CHN/2016	China	2016	feces
MH004008.1	CVA16/Shenzhen534/CHN/2016	China	2016	feces
MH004009.1	CVA16/Shenzhen536/CHN/2016	China	2016	anal swab

MH004011.1	CVA16/Shenzhen570/CHN/2016	China	2016	feces
MH004013.1	CVA16/Shenzhen581/CHN/2016	China	2016	feces
MH004014.1	CVA16/Shenzhen596/CHN/2016	China	2016	feces
MH004015.1	CVA16/Shenzhen599/CHN/2016	China	2016	anal swab
MH004016.1	CVA16/Shenzhen609/CHN/2016	China	2016	feces
MH004017.1	CVA16/Shenzhen618/CHN/2016	China	2016	anal swab
MH004018.1	CVA16/Shenzhen74/CHN/2017	China	2017	anal swab
MH004019.1	CVA16/Shenzhen90/CHN/2017	China	2017	anal swab
MH004020.1	CVA16/Shenzhen128/CHN/2017	China	2017	anal swab
MH004021.1	CVA16/Shenzhen130/CHN/2017	China	2017	anal swab
MH004022.1	CVA16/Shenzhen133/CHN/2017	China	2017	feces
MH004023.1	CVA16/Shenzhen173/CHN/2017	China	2017	anal swab
MH004024.1	CVA16/Shenzhen194/CHN/2017	China	2017	feces
MH004025.1	CVA16/Shenzhen204/CHN/2017	China	2017	anal swab
MH004026.1	CVA16/Shenzhen206/CHN/2017	China	2017	anal swab
MH004027.1	CVA16/Shenzhen208/CHN/2017	China	2017	anal swab
MH004028.1	CVA16/Shenzhen231/CHN/2017	China	2017	feces
MH004029.1	CVA16/Shenzhen259/CHN/2017	China	2017	feces
MH004030.1	CVA16/Shenzhen277/CHN/2017	China	2017	feces
MH004031.1	CVA16/Shenzhen284/CHN/2017	China	2017	feces
MH004032.1	CVA16/Shenzhen292/CHN/2017	China	2017	feces
MH004033.1	CVA16/Shenzhen299/CHN/2017	China	2017	feces
MH004034.1	CVA16/Shenzhen301/CHN/2017	China	2017	feces
MH004035.1	CVA16/Shenzhen324/CHN/2017	China	2017	anal swab
MH004036.1	CVA16/Shenzhen335/CHN/2017	China	2017	feces
MH004037.1	CVA16/Shenzhen375/CHN/2017	China	2017	feces
MH004038.1	CVA16/Shenzhen425/CHN/2017	China	2017	feces
MH004039.1	CVA16/Shenzhen458/CHN/2017	China	2017	anal swab
MH004040.1	CVA16/Shenzhen459/CHN/2017	China	2017	feces
MH004041.1	CVA16/Shenzhen460/CHN/2017	China	2017	feces
MH004042.1	CVA16/Shenzhen464/CHN/2017	China	2017	feces
MH004043.1	CVA16/Shenzhen482/CHN/2017	China	2017	feces
MH004044.1	CVA16/Shenzhen541/CHN/2017	China	2017	feces
MH004045.1	CVA16/Shenzhen670/CHN/2017	China	2017	feces
MH004046.1	CVA16/Shenzhen709/CHN/2017	China	2017	anal swab
MH004047.1	CVA16/Shenzhen710/CHN/2017	China	2017	anal swab
MH004048.1	CVA16/Shenzhen744/CHN/2017	China	2017	feces
MH010198.1	CVA16/Shenzhen500/CHN/2014	China	2014	anal swab
MH010199.1	CVA16/Shenzhen469/CHN/2015	China	2015	feces
MH010200.1	CVA16/Shenzhen87/CHN/2016	China	2016	feces
MH010201.1	CVA16/Shenzhen289/CHN/2016	China	2016	feces
MH010202.1	CVA16/Shenzhen76/CHN/2017	China	2017	feces
MH010203.1	CVA16/Shenzhen169/CHN/2017	China	2017	feces

MH010204.1	CVA16/Shenzhen174/CHN/2017	China	2017	anal swab
MH010205.1	CVA16/Shenzhen189/CHN/2017	China	2017	feces
MH084318.1	Env-15-022-A16a	United Kingdom	2015	feces
MH084319.1	Env-15-022-A16b	United Kingdom	2015	feces
MH084325.1	Env-16-015-A16	United Kingdom	2016	feces
MH160022.1	CVA16/SDJN410/CHN/2017	China	2017	feces
MH160023.1	CVA16/SDJN164/CHN/2017	China	2017	feces
MH160024.1	CVA16/SDJN144/CHN/2017	China	2017	feces
MH160025.1	CVA16/SDJN142/CHN/2017	China	2017	feces
MH160026.1	CVA16/SDJN129/CHN/2017	China	2017	feces
MH160027.1	CVA16/SDJN101/CHN/2017	China	2017	feces
MH160028.1	CVA16/SDJN100/CHN/2017	China	2017	feces
MH160030.1	CVA16/SDJN058/CHN/2017	China	2017	feces
MH160031.1	CVA16/SDJN046/CHN/2017	China	2017	feces
MH167447.1	LS-2017-06	China	2017	throat swab
MH167448.1	LS-2017-07	China	2017	throat swab
MH167449.1	LS-2017-08	China	2017	throat swab
MH361019.1	SCO_SWG_2015_Coxsackievirus_A16.1	United Kingdom	2015	feces
MH361023.1	SCO_SWG_2015_Coxsackievirus_A16.2	United Kingdom	2015	feces
MH491122.1	B0155(Wuxi/2014)	China	2014	feces
MH491123.1	B410(Wuxi/2011)	China	2011	feces
MH491125.1	E052(Suzhou/2012)	China	2012	feces
MH491126.1	E103(Suzhou/2012)	China	2012	feces
MH491127.1	E209(Suzhou/2012)	China	2012	feces
MH491128.1	E231(Suzhou/2012)	China	2012	feces
MH491129.1	E412(Suzhou/2011)	China	2011	feces
MH491130.1	E425(Suzhou/2012)	China	2012	feces
MH491132.1	E474(Suzhou/2012)	China	2012	feces
MH491133.1	E480(Suzhou/2012)	China	2012	feces
MH491134.1	E889(Suzhou/2012)	China	2012	feces
MH491135.1	G06(Lianyungang/2015)	China	2015	feces
MH491136.1	J914(Yancheng/2011)	China	2011	feces
MH491137.1	J9132(Yancheng/2011)	China	2011	feces
MH491138.1	J9152(Yancheng/2011)	China	2011	feces
MH491139.1	J9155(Yancheng/2011)	China	2011	feces
MH491140.1	NJ330(Nanjing/2011)	China	2011	feces
MH516915.1	C041(Xuzhou2017)	China	2017	feces
MH516916.1	E408(Suzhou2017)	China	2017	feces

MH516917.1	E451(Suzhou2017)	China	2017	feces
MH516918.1	HA109(Huai_an2017)	China	2017	feces
MH516919.1	HA283(Huai_an2017)	China	2017	feces
MH516920.1	J834(Yancheng2017)	China	2017	feces
MH516921.1	J9020(Yancheng2017)	China	2017	feces
MH516922.1	K17239(Yangzhou2017)	China	2017	feces
MH516927.1	C85(Xuzhou2016)	China	2016	feces
MH516928.1	C143C85(Xuzhou2016)	China	2016	feces
MH516929.1	GD03(Lianyungang2016)	China	2016	feces
MH516930.1	GD09(Lianyungang2016)	China	2016	feces
MH516931.1	HA172(Huai_an2016)	China	2016	feces
MH516932.1	HA289(Huai_an2016)	China	2016	feces
MH516935.1	B410(Wuxi2011)	China	2011	feces
MH516936.1	NJ330(Nanjing2011)	China	2011	feces
MH516937.1	GC0136(Lianyungang2011)	China	2011	feces
MH516938.1	GG0156(Lianyungang2011)	China	2011	feces
MH516939.1	B094(Wuxi2012)	China	2012	feces
MH516940.1	B130(Wuxi2012)	China	2012	feces
MH516941.1	B132(Wuxi2012)	China	2012	feces
MH516942.1	BX12-034(Nanjing2012)	China	2012	feces
MH516943.1	C23(Xuzhou2012)	China	2012	feces
MH516944.1	C179(Xuzhou2012)	China	2012	feces
MH516945.1	C205(Xuzhou2012)	China	2012	feces
MH516946.1	C213(Xuzhou2012)	China	2012	feces
MH516947.1	D004(Changzhou2012)	China	2012	feces
MH516949.1	FQD48(Nantong2012)	China	2012	feces
MH516950.1	FTZ027(Nantong2012)	China	2012	feces
MH516951.1	G011(Lianyungang2012)	China	2012	feces
MH516952.1	G018(Lianyungang2012)	China	2012	feces
MH516954.1	J149(Yancheng2012)	China	2012	feces
MH516955.1	J311(Yancheng2012)	China	2012	feces
MH516957.1	J848(Yancheng2012)	China	2012	feces
MH516959.1	B004(Wuxi2013)	China	2013	feces
MH516961.1	D187(Changzhou2013)	China	2013	feces
MH516965.1	J855(Yancheng2013)	China	2013	feces
MH516966.1	K13053(Yangzhou2013)	China	2013	feces
MH516967.1	K13150(Yangzhou2013)	China	2013	feces
MH516968.1	K13208(Yangzhou2013)	China	2013	feces
MH516970.1	SG007(Zhengjiang2014)	China	2014	feces
MH516973.1	B002(Wuxi2014)	China	2014	feces
MH516974.1	B008(Wuxi2014)	China	2014	feces
MH516975.1	B013Wuxi2014	China	2014	feces
MH516976.1	B014(Wuxi2014)	China	2014	feces

MH516978.1	E002(Suzhou2014)	China	2014	feces
MH516980.1	FHA061(Nantong2014)	China	2014	feces
MH516981.1	G03(Lianyungang2014)	China	2014	feces
MH516982.1	J222(Yancheng2014)	China	2014	feces
MH516984.1	J435(Yancheng2014)	China	2014	feces
MH516988.1	NJ-GL031(Nanjin2014)	China	2014	feces
MH516989.1	NJ-PK042(Nanjin2014)	China	2014	feces
MH516990.1	NJ-YH031(Nanjin2014)	China	2014	feces
MH516991.1	SA66(Zhengjiang2014)	China	2014	feces
MH516992.1	SC010(Zhengjiang2014)	China	2014	feces
MH516994.1	SE254(Zhengjiang2014)	China	2014	feces
MH516995.1	SF90(Zhengjiang2014)	China	2014	feces
MH516996.1	E138(Suzhou2015)	China	2015	feces
MH516997.1	E184(Suzhou2015)	China	2015	feces
MH516998.1	E287(Suzhou2015)	China	2015	feces
MH516999.1	E321(Suzhou2015)	China	2015	feces
MH517000.1	E389(Suzhou2015)	China	2015	feces
MH517001.1	E433(Suzhou2015)	China	2015	feces
MH517002.1	FHM410(Nantong2015)	China	2015	feces
MH517003.1	J609(Yancheng2015)	China	2015	feces
MH517004.1	J802(Yancheng2015)	China	2015	feces
MH517005.1	J818(Yancheng2015)	China	2015	feces
MH517006.1	J839(Yancheng2015)	China	2015	feces
MH517007.1	J3005(Yancheng2015)	China	2015	feces
MH517008.1	JT47(Yancheng2015)	China	2015	feces
MH517009.1	K15106(Yangzhou2015)	China	2015	feces
MH517010.1	K15321(Yangzhou2015)	China	2015	feces
MH517011.1	K15335(Yangzhou2015)	China	2015	feces
MH517015.1	SD14(Zhengjiang2015)	China	2015	feces
MH517016.1	SD15(Zhengjiang2015)	China	2015	feces
MH517017.1	SE281(Zhengjiang2015)	China	2015	feces
MH517018.1	tn056(Changzhou2015)	China	2015	feces
MH644614.1	CA16-3/SZLG/2017	China	2017	anal swab
MH644615.1	CA16-6/SZLG/2017	China	2017	anal swab
MH644616.1	CA16-7/SZLG/2017	China	2017	anal swab
MH644617.1	CA16-8/SZLG/2017	China	2017	anal swab
MH644618.1	CA16-10/SZLG/2017	China	2017	anal swab
MH644619.1	CA16-11/SZLG/2017	China	2017	anal swab
MH644620.1	CA16-12/SZLG/2017	China	2017	anal swab
MH644621.1	CA16-13/SZLG/2017	China	2017	anal swab
MH644622.1	CA16-16/SZLG/2017	China	2017	anal swab
MH644623.1	CA16-17/SZLG/2017	China	2017	anal swab
MH644624.1	CA16-19/SZLG/2017	China	2017	anal swab

MH644625.1	CA16-21/SZLG/2017	China	2017	anal swab
MH644626.1	CA16-22/SZLG/2017	China	2017	anal swab
MH644627.1	CA16-23/SZLG/2017	China	2017	anal swab
MH644628.1	CA16-24/SZLG/2017	China	2017	anal swab
MH644629.1	CA16-25/SZLG/2017	China	2017	anal swab
MH644630.1	CA16-26/SZLG/2017	China	2017	anal swab
MH644631.1	CA16-27/SZLG/2017	China	2017	anal swab
MH716172.1	CVA16/sHFMD03/Shenzhen/2016	China	2016	feces
MH780757.1	oV18-026	India	2018	feces
MH796400.1	V18-022	India	2018	feces
MK348673.1	FL14044T-Anhui	China	2014	throat swab
MK348674.1	FL14052T-Anhui	China	2014	throat swab
MK348675.1	FL14053T-Anhui	China	2014	throat swab
MK348678.1	FL14076T-Anhui	China	2014	throat swab
MK348679.1	FL14077T-Anhui	China	2014	throat swab
MK348680.1	FL14093T-Anhui	China	2014	throat swab
MK348681.1	FL14094T-Anhui	China	2014	throat swab
MK348682.1	FL14105T-Anhui	China	2014	throat swab
MK348683.1	FL14106T-Anhui	China	2014	throat swab
MK348684.1	FL14140T-Anhui	China	2014	throat swab
MK348685.1	FL14329T-Anhui	China	2014	throat swab
MK348686.1	FL14332T-Anhui	China	2014	throat swab
MK348687.1	FL14335T-Anhui	China	2014	throat swab
MK348688.1	FL14372T-Anhui	China	2014	throat swab
MK348689.1	FL14419T-Anhui	China	2014	throat swab
MK357093.1	CVA16/JN330/CHN/2018	China	2018	feces
MK357094.1	CVA16/JN255/CHN/2018	China	2018	feces
MK357095.1	CVA16/JN206/CHN/2018	China	2018	feces
MK357096.1	CVA16/JN191/CHN/2018	China	2018	feces
MK357097.1	CVA16/JN113/CHN/2018	China	2018	feces
MK357098.1	CVA16/JN085/CHN/2018	China	2018	feces
MK357099.1	CVA16/JN049/CHN/2018	China	2018	feces
MK357100.1	CVA16/JN023/CHN/2018	China	2018	feces
MK357101.1	CVA16/JN016/CHN/2018	China	2018	feces
MK357102.1	CVA16/JN428/CHN/2018	China	2018	feces
MK697682.1	Sydney48	Australia	2013	feces
MN046208.1	KM/M08	China	2015	throat swab
MN153798.1	USA/2015/CA-RGDS-1044	USA	2015	cerebrospinal fluid
MN205421.1	CVA16/Shenzhen49/CHN/2017	China	2017	feces
MN205422.1	CVA16/Shenzhen67/CHN/2017	China	2017	feces
MN205423.1	CVA16/Shenzhen73/CHN/2017	China	2017	feces
MN205424.1	CVA16/Shenzhen83/CHN/2017	China	2017	feces
MN205426.1	CVA16/Shenzhen312/CHN/2016	China	2016	feces

MN205427.1	CVA16/Shenzhen320/CHN/2016	China	2016	feces
MN205429.1	CVA16/Shenzhen364/CHN/2016	China	2016	feces
MN337592.1	S0295B	China	2013	feces
MN337593.1	S1082b	China	2013	feces
MN337595.1	S0548B	China	2013	feces
MN337596.1	S0988B	China	2013	feces
MN337597.1	S1091b	China	2013	feces
MN337598.1	S0960B	China	2013	feces
MN337599.1	S0266B	China	2013	feces
MN337603.1	S0511b/AH/2013	China	2013	anal swab
MN541006.1	CV-A16/SWG38/SD/CHN/2018	China	2018	feces
MN541018.1	CV-A16/SWG50/SD/CHN/2018	China	2018	feces
MN541025.1	CV-A16/SWG57/SD/CHN/2018	China	2018	feces
MN886521.1	S7628/BJ/CHN/2019	China	2019	throat swab
MN886522.1	S7624/BJ/CHN/2019	China	2019	throat swab
MT211988.1	AH17-18/AH/East/CHN/2017-02-12	China	2017	throat swab
MT211989.1	AH18-25/AH/East/CHN/2018-04-26	China	2018	throat swab
MT211990.1	BJ16-68/BJ/Central/CHN/2016-08-01	China	2016	throat swab
MT211991.1	BJ17-14/BJ/Central/CHN/2017-02-15	China	2017	throat swab
MT211992.1	BJ18-58/BJ/Central/CHN/2018-08-20	China	2018	throat swab
MT211993.1	CQ16-104/CQ/West/CHN/2016-01-13	China	2016	throat swab
MT211994.1	CQ17-38/CQ/West/CHN/2017-04-12	China	2017	throat swab
MT211995.1	CQ18-3/CQ/West/CHN/2018-06-28	China	2018	throat swab
MT211996.1	GD16-105/GD/South/CHN/2016-06-17	China	2016	feces
MT211997.1	GD17-81/GD/South/CHN/2017-06-01	China	2017	feces
MT211998.1	GD18-104/GD/South/CHN/2018-08-14	China	2018	feces
MT211999.1	GS16-110/GS/West/CHN/2016-05-30	China	2016	feces
MT212000.1	GS17-636/GS/West/CHN/2017-06-15	China	2017	throat swab
MT212001.1	GS17-658/GS/West/CHN/2017-06-15	China	2017	feces
MT212002.1	GS18-179/GS/West/CHN/2018-05-14	China	2018	feces
MT212003.1	GZ16-QN035/GZ/South/CHN/2016-06-30	China	2016	throat swab
MT212004.1	HAN17-66/HaN/South/CHN/2017-12-11	China	2017	throat swab
MT212005.1	HAN18-8/HaN/South/CHN/2018-02-28	China	2018	throat swab
MT212006.1	HB16-54015/HB/Central/CHN/2016-01-15	China	2016	throat swab
MT212007.1	HB17-54371/HB/Central/CHN/2017-07-07	China	2017	throat swab
MT212008.1	HB18-20/HB/Central/CHN/2018-05-16	China	2018	anal swab
MT212009.1	HEN17-108/HeN/Central/CHN/2017-06-30	China	2017	feces
MT212010.1	HeN18-400/HeN/Central/CHN/2018-07-11	China	2018	feces
MT212011.1	HLJ16-HH2016033/HLJ/North/CHN/2016-09-19	China	2016	throat swab
MT212012.1	HLJ18-16/HLJ/North/CHN/2018-08-05	China	2018	throat swab
MT212013.1	HuN16-75/HuN/South/CHN/2016-05-09	China	2016	feces
MT212014.1	HUN17-33/HuN/South/CHN/2017-08-07	China	2017	anal swab

MT212015.1	HuN18-5/HuN/South/CHN/2018-02-26	China	2018	throat swab
MT212016.1	JL18-97/JL/North/CHN/2018-08-05	China	2018	feces
MT212017.1	JX16-126/JX/East/CHN/2016-12-27	China	2016	throat swab
MT212018.1	JX18-30/JX/East/CHN/2018-03-26	China	2018	throat swab
MT212019.1	LN16-23-12/LN/North/CHN/2016-07-11	China	2016	throat swab
MT212020.1	QH16-8/QH/West/CHN/2016-07-07	China	2016	throat swab
MT212021.1	QH17-104/QH/West/CHN/2017-10-13	China	2017	throat swab
MT212022.1	QH18-5/QH/West/CHN/2018-05-12	China	2018	throat swab
MT212023.1	SAX17-50/SaX/Central/CHN/2017-09-16	China	2017	anal swab
MT212024.1	SD16-101/SD/East/CHN/2016-07-22	China	2016	feces
MT212025.1	SX17-286/SX/Central/CHN/2017-06-16	China	2017	feces
MT212026.1	TJ16-7/TJ/Central/CHN/2016-02-29	China	2016	feces
MT212027.1	TJ17-36/TJ/Central/CHN/2017-03-13	China	2017	feces
MT212028.1	TJ18-63/TJ/Central/CHN/2018-05-28	China	2018	feces
MT212029.1	XJ17-212/XJ/West/CHN/2017-10-04	China	2017	feces
MT212030.1	XJ18-019/XJ/West/CHN/2018-04-09	China	2018	feces
MT212031.1	YN16-253/YN/South/CHN/2016-05-17	China	2016	feces
MT212032.1	YN17-J29/YN/South/CHN/2017-02-07	China	2017	feces
MT212033.1	YN18-A67/YN/South/CHN/2018-03-14	China	2018	feces
MT212034.1	ZJ16-14/ZJ/East/CHN/2016-03-30	China	2016	throat swab
MT212035.1	ZJ17-91/ZJ/East/CHN/2017-07-02	China	2017	throat swab
MT212036.1	ZJ18-24/ZJ/East/CHN/2018-01-04	China	2018	throat swab
MT553115.1	S498/BJ/CHN/2010	China	2010	throat swab
MT553116.1	S517/BJ/CHN/2010	China	2010	throat swab
MT553117.1	S614/BJ/CHN/2010	China	2010	throat swab
MT553118.1	S668/BJ/CHN/2010	China	2010	throat swab
MT553119.1	S701/BJ/CHN/2010	China	2010	throat swab
MT553120.1	S721/BJ/CHN/2010	China	2010	throat swab
MT553121.1	S806/BJ/CHN/2010	China	2010	throat swab
MT553122.1	S854/BJ/CHN/2010	China	2010	throat swab
MT553123.1	S883/BJ/CHN/2011	China	2011	throat swab
MT553124.1	S908/BJ/CHN/2011	China	2011	throat swab
MT553125.1	S919/BJ/CHN/2011	China	2011	throat swab
MT553126.1	S928/BJ/CHN/2011	China	2011	throat swab
MT553127.1	S975/BJ/CHN/2011	China	2011	throat swab
MT553128.1	S995/BJ/CHN/2011	China	2011	throat swab
MT553129.1	S1002/BJ/CHN/2011	China	2011	throat swab
MT553130.1	S1021/BJ/CHN/2011	China	2011	throat swab
MT553131.1	S1035/BJ/CHN/2011	China	2011	throat swab
MT553132.1	S1074/BJ/CHN/2012	China	2012	throat swab
MT553133.1	S1099/BJ/CHN/2012	China	2012	throat swab
MT553134.1	S1103/BJ/CHN/2012	China	2012	throat swab
MT553135.1	S1104/BJ/CHN/2012	China	2012	throat swab

MT553136.1	S1108/BJ/CHN/2012	China	2012	throat swab
MT553137.1	S1135/BJ/CHN/2012	China	2012	throat swab
MT553138.1	S1149/BJ/CHN/2012	China	2012	throat swab
MT553139.1	S1168/BJ/CHN/2012	China	2012	throat swab
MT553140.1	S1817/BJ/CHN/2013	China	2013	throat swab
MT553141.1	S1841/BJ/CHN/2013	China	2013	throat swab
MT553142.1	S1849/BJ/CHN/2013	China	2013	throat swab
MT553143.1	S1851/BJ/CHN/2013	China	2013	throat swab
MT553144.1	S1870/BJ/CHN/2013	China	2013	throat swab
MT553145.1	S1874/BJ/CHN/2013	China	2013	throat swab
MT553146.1	S1877/BJ/CHN/2013	China	2013	throat swab
MT553147.1	S1905/BJ/CHN/2013	China	2013	throat swab
MT553148.1	S1922/BJ/CHN/2013	China	2013	throat swab
MT553149.1	S1927/BJ/CHN/2013	China	2013	throat swab
MT553150.1	S1939/BJ/CHN/2013	China	2013	throat swab
MT553151.1	S1945/BJ/CHN/2013	China	2013	throat swab
MT553152.1	S1960/BJ/CHN/2013	China	2013	throat swab
MT553153.1	S2046/BJ/CHN/2014	China	2014	throat swab
MT553154.1	S2262/BJ/CHN/2014	China	2014	throat swab
MT553155.1	S2297/BJ/CHN/2014	China	2014	throat swab
MT553156.1	S2343/BJ/CHN/2014	China	2014	throat swab
MT553157.1	S2349/BJ/CHN/2014	China	2014	throat swab
MT553158.1	S2559/BJ/CHN/2014	China	2014	throat swab
MT553159.1	S2574/BJ/CHN/2014	China	2014	throat swab
MT553160.1	S2575/BJ/CHN/2014	China	2014	throat swab
MT553161.1	S2587/BJ/CHN/2014	China	2014	throat swab
MT553162.1	S2847/BJ/CHN/2014	China	2014	throat swab
MT553163.1	S2880/BJ/CHN/2014	China	2014	throat swab
MT553164.1	S2911/BJ/CHN/2014	China	2014	throat swab
MT553165.1	S2999/BJ/CHN/2015	China	2015	throat swab
MT553166.1	S3126/BJ/CHN/2015	China	2015	throat swab
MT553167.1	S3160/BJ/CHN/2015	China	2015	throat swab
MT553168.1	S3300/BJ/CHN/2015	China	2015	throat swab
MT553169.1	S3347/BJ/CHN/2015	China	2015	throat swab
MT553170.1	S3370/BJ/CHN/2015	China	2015	throat swab
MT553171.1	S3407/BJ/CHN/2015	China	2015	throat swab
MT553172.1	S3446/BJ/CHN/2015	China	2015	throat swab
MT553173.1	S3450/BJ/CHN/2015	China	2015	throat swab
MT553174.1	S3473/BJ/CHN/2016	China	2016	throat swab
MT553175.1	S3482/BJ/CHN/2016	China	2016	throat swab
MT553176.1	S3484/BJ/CHN/2016	China	2016	throat swab
MT553177.1	S3514/BJ/CHN/2016	China	2016	throat swab
MT553178.1	S3540/BJ/CHN/2016	China	2016	throat swab

MT553179.1	S3582/BJ/CHN/2016	China	2016	throat swab
MT553180.1	S3613/BJ/CHN/2016	China	2016	throat swab
MT553181.1	S3657/BJ/CHN/2016	China	2016	throat swab
MT553182.1	S3666/BJ/CHN/2016	China	2016	throat swab
MT553183.1	S3720/BJ/CHN/2016	China	2016	throat swab
MT553184.1	S3727/BJ/CHN/2016	China	2016	throat swab
MT553185.1	S3741/BJ/CHN/2016	China	2016	throat swab
MT553186.1	S3746/BJ/CHN/2016	China	2016	throat swab
MT553187.1	S3770/BJ/CHN/2016	China	2016	throat swab
MT553188.1	S3812/BJ/CHN/2016	China	2016	throat swab
MT553189.1	S3866/BJ/CHN/2016	China	2016	throat swab
MT553190.1	S4130/BJ/CHN/2016	China	2016	throat swab
MT553191.1	S4131/BJ/CHN/2016	China	2016	throat swab
MT553192.1	S4184/BJ/CHN/2017	China	2017	throat swab
MT553193.1	S4223/BJ/CHN/2017	China	2017	throat swab
MT553194.1	S4618/BJ/CHN/2017	China	2017	throat swab
MT553195.1	S4739/BJ/CHN/2017	China	2017	throat swab
MT553196.1	S5066/BJ/CHN/2017	China	2017	throat swab
MT553197.1	S5153/BJ/CHN/2018	China	2018	throat swab
MT553198.1	S5343/BJ/CHN/2018	China	2018	throat swab
MT553199.1	S5485/BJ/CHN/2018	China	2018	throat swab
MT553200.1	S5515/BJ/CHN/2018	China	2018	throat swab
MT553201.1	S5558/BJ/CHN/2018	China	2018	throat swab
MT553202.1	S5563/BJ/CHN/2018	China	2018	throat swab
MT553203.1	S5592/BJ/CHN/2018	China	2018	throat swab
MT553204.1	S5607/BJ/CHN/2018	China	2018	throat swab
MT553205.1	S5616/BJ/CHN/2018	China	2018	throat swab
MT553206.1	S5619/BJ/CHN/2018	China	2018	throat swab
MT553207.1	S5626/BJ/CHN/2018	China	2018	throat swab
MT553208.1	S5667/BJ/CHN/2018	China	2018	throat swab
MT553209.1	S5687/BJ/CHN/2018	China	2018	throat swab
MT553210.1	S5734/BJ/CHN/2018	China	2018	throat swab
MT553211.1	S5803/BJ/CHN/2018	China	2018	throat swab
MT553212.1	S5822/BJ/CHN/2018	China	2018	throat swab
MT553213.1	S5825/BJ/CHN/2018	China	2018	throat swab
MT553214.1	S5855/BJ/CHN/2018	China	2018	throat swab
MT553215.1	S5862/BJ/CHN/2018	China	2018	throat swab
MT553216.1	S5876/BJ/CHN/2018	China	2018	throat swab
MT553217.1	S5916/BJ/CHN/2018	China	2018	throat swab
MT553218.1	S5940/BJ/CHN/2018	China	2018	throat swab
MT553219.1	S6011/BJ/CHN/2018	China	2018	throat swab
MT553220.1	S6020/BJ/CHN/2018	China	2018	throat swab
MT553221.1	S6756/BJ/CHN/2019	China	2019	throat swab

MT553222.1	S6821/BJ/CHN/2019	China	2019	throat swab
MT553223.1	S6836/BJ/CHN/2019	China	2019	throat swab
MT553224.1	S6853/BJ/CHN/2019	China	2019	throat swab
MT553225.1	S6854/BJ/CHN/2019	China	2019	throat swab
MT553226.1	S6859/BJ/CHN/2019	China	2019	throat swab
MT553227.1	S6879/BJ/CHN/2019	China	2019	throat swab
MT553228.1	S6880/BJ/CHN/2019	China	2019	throat swab
MT553229.1	S6904/BJ/CHN/2019	China	2019	throat swab
MT553230.1	S6908/BJ/CHN/2019	China	2019	throat swab
MT553231.1	S6926/BJ/CHN/2019	China	2019	throat swab
MT553232.1	S6932/BJ/CHN/2019	China	2019	throat swab
MT553233.1	S7094/BJ/CHN/2019	China	2019	throat swab
MT553234.1	S7147/BJ/CHN/2019	China	2019	throat swab
MT553235.1	S7367/BJ/CHN/2019	China	2019	throat swab
MT553236.1	S7374/BJ/CHN/2019	China	2019	throat swab
MT553237.1	S7438/BJ/CHN/2019	China	2019	throat swab
MT553238.1	S7443/BJ/CHN/2019	China	2019	throat swab
MT553239.1	S7469/BJ/CHN/2019	China	2019	throat swab
MT553240.1	S7484/BJ/CHN/2019	China	2019	throat swab
MT553241.1	S7566/BJ/CHN/2019	China	2019	throat swab
MT577654.1	NIV1850130/CVA-16/Pune/2018/India	India	2018	vesicle swab
MT577655.1	NIV1850166/CVA-16/Pune/2018/India	India	2018	vesicle swab
MT577656.1	NIV1850125/CVA-16/Pune/2018/India	India	2018	vesicle swab
MT577657.1	NIV1850158/CVA-16/Pune/2018/India	India	2018	vesicle swab
MT577658.1	NIV1850124/CVA-16/Pune/2018/India	India	2018	throat swab
MT577659.1	NIV1850123/CVA-16/Pune/2018/India	India	2018	vesicle swab
MT577660.1	NIV1850113/CVA-16/Pune/2018/India	India	2018	vesicle swab
MT577661.1	NIV1850112/CVA-16/Pune/2018/India	India	2018	throat swab
MT577662.1	NIV1850111/CVA-16/Pune/2018/India	India	2018	vesicle swab
MT577663.1	NIV1850164/CVA-16/Pune/2018/India	India	2018	vesicle swab
MT577664.1	NIV1850163/CVA-16/Pune/2018/India	India	2018	vesicle swab
MT577665.1	NIV1850153/CVA-16/Pune/2018/India	India	2018	vesicle swab
MT577666.1	NIV1850155/CVA-16/Pune/2018/India	India	2018	vesicle swab
MT577667.1	NIV1850154/CVA-16/Pune/2018/India	India	2018	throat swab
MT577668.1	NIV1850132/CVA-16/Pune/2018/India	India	2018	vesicle swab
MT577669.1	NIV1850140/CVA-16/Pune/2018/India	India	2018	vesicle swab
MT577670.1	NIV1850084/CVA-16/Pune/2018/India	India	2018	throat swab
MT577671.1	NIV1850085/CVA-16/Pune/2018/India	India	2018	vesicle swab
MT577672.1	NIV1850086/CVA-16/Pune/2018/India	India	2018	throat swab
MT577673.1	NIV1841810/CVA-16/Pune/2018/India	India	2018	vesicle swab
MT577674.1	NIV1841809/CVA-16/Pune/2018/India	India	2018	vesicle swab
MT577675.1	NIV1850087/CVA-16/Pune/2018/India	India	2018	vesicle swab
MT577676.1	NIV1850170/CVA-16/Pune/2018/India	India	2018	vesicle swab

MT577677.1	NIV1850173/CVA-16/Pune/2018/India	India	2018	vesicle swab
MT577678.1	NIV1850169/CVA-16/Pune/2018/India	India	2018	vesicle swab
MT577679.1	NIV1850167/CVA-16/Pune/2018/India	India	2018	vesicle swab
MT577680.1	NIV1850127/CVA-16/Pune/2018/India	India	2018	vesicle swab
MT577681.1	NIV1850142/CVA-16/Pune/2018/India	India	2018	vesicle swab
MT577682.1	NIV1850143/CVA-16/Pune/2018/India	India	2018	vesicle swab
MT577683.1	NIV1850114/CVA-16/Pune/2018/India	India	2018	throat swab
MT577684.1	NIV1841374/CVA-16/Pune/2018/India	India	2018	vesicle swab
MT577685.1	NIV1850180/CVA-16/Pune/2018/India	India	2018	vesicle swab
MT577686.1	NIV1850097/CVA-16/Pune/2018/India	India	2018	throat swab
MT577689.1	NIV1850148/CVA-16/Pune/2018/India	India	2018	vesicle swab
MT577690.1	NIV1850150/CVA-16/Pune/2018/India	India	2018	throat swab
MT577691.1	NIV1850151/CVA-16/Pune/2018/India	India	2018	vesicle swab
MT577692.1	NIV1841626/CVA-16/Pune/2018/India	India	2018	vesicle swab
MT577693.1	NIV1850177/CVA-16/Pune/2018/India	India	2018	vesicle swab
MT577694.1	NIV1850147/CVA-16/Pune/2018/India	India	2018	throat swab
MT641380.1	CLI-B1-29-CV-A16	United Kingdom	2017	throat swab
MT641412.1	CLI-B3-24-CV-A16	United Kingdom	2018	feces
MT641429.1	CLI-B3-51-CV-A16	United Kingdom	2018	throat swab
MW462132.1	S2540/BJ/CHN/2014	China	2014	throat swab
MW462133.1	S2814/BJ/CHN/2014	China	2014	throat swab
MW462134.1	S2592/BJ/CHN/2014	China	2014	throat swab
MW462135.1	S2395/BJ/CHN/2014	China	2014	throat swab
MW462136.1	S2366/BJ/CHN/2014	China	2014	throat swab
MW462137.1	S2248/BJ/CHN/2014	China	2014	throat swab
MW462138.1	S2241/BJ/CHN/2014	China	2014	throat swab
MW462139.1	S2192/BJ/CHN/2014	China	2014	throat swab
MW462140.1	S2165/BJ/CHN/2014	China	2014	throat swab
MW462141.1	S2157/BJ/CHN/2014	China	2014	throat swab
MW462142.1	S2032/BJ/CHN/2014	China	2014	throat swab
MW462143.1	S1131/BJ/CHN/2012	China	2012	throat swab
MW462144.1	S1125/BJ/CHN/2012	China	2012	throat swab
MW462145.1	S1060/BJ/CHN/2012	China	2012	throat swab
MW462146.1	S1058/BJ/CHN/2012	China	2012	throat swab
MW462147.1	S2497/BJ/CHN/2014	China	2014	throat swab
MW462148.1	S2472/BJ/CHN/2014	China	2014	throat swab
MW462149.1	S2419/BJ/CHN/2014	China	2014	throat swab
MW462150.1	S530/BJ/CHN/2010	China	2010	throat swab
MW462151.1	S503/BJ/CHN/2010	China	2010	throat swab
MW462152.1	S478/BJ/CHN/2010	China	2010	throat swab

MW462153.1	S980/BJ/CHN/2011	China	2011	throat swab
MW462154.1	S970/BJ/CHN/2011	China	2011	throat swab
MW462155.1	S965/BJ/CHN/2011	China	2011	throat swab
MW462156.1	S951/BJ/CHN/2011	China	2011	throat swab
MW462157.1	S712/BJ/CHN/2010	China	2010	throat swab
MW462158.1	S705/BJ/CHN/2010	China	2010	throat swab
MW462159.1	S686/BJ/CHN/2010	China	2010	throat swab
MW462160.1	S670/BJ/CHN/2010	China	2010	throat swab
MW462161.1	S603/BJ/CHN/2010	China	2010	throat swab
MW462162.1	S591/BJ/CHN/2010	China	2010	throat swab
MW462163.1	S565/BJ/CHN/2010	China	2010	throat swab
MW462164.1	S540/BJ/CHN/2010	China	2010	throat swab
MW462165.1	S4923/BJ/CHN/2017	China	2017	throat swab
MW462166.1	S4453/BJ/CHN/2017	China	2017	throat swab
MW462167.1	S4285/BJ/CHN/2017	China	2017	throat swab
MW462168.1	S4220/BJ/CHN/2017	China	2017	throat swab
OL470922.1	CV-A16/HA19108/2019/Beijing/Tongzhou	China	2019	throat swab
OL470923.1	CV-A16/HA19090/2019/Beijing/Tongzhou	China	2019	throat swab
OL470924.1	CV-A16/HA19091/2019/Beijing/Tongzhou	China	2019	throat swab
OL470925.1	CV-A16/HA19027/2019/Beijing/Tongzhou	China	2019	throat swab
OL470926.1	CV-A16/HA19068/2019/Beijing/Tongzhou	China	2019	throat swab
OL470927.1	CV-A16/HA19105/2019/Beijing/Tongzhou	China	2019	throat swab
OL470928.1	CV-A16/HA19053/2019/Beijing/Tongzhou	China	2019	throat swab
OL470929.1	CV-A16/HA19082/2019/Beijing/Tongzhou	China	2019	throat swab
OL470930.1	CV-A16/HA20005/2020/Beijing/Tongzhou	China	2020	throat swab
ON646272.1	Y-22/CQ/CHN/2018	China	2018	feces
U05876.1	G-10	South Africa	1951	feces
